# The role of FOXK2–FBXO32 in breast cancer tumorigenesis: Insights into ribosome‐associated pathways

**DOI:** 10.1111/1759-7714.15482

**Published:** 2024-11-18

**Authors:** Fuben Liao, Jinjin Zhu, Junju He, Zheming Liu, Yi Yao, Qibin Song

**Affiliations:** ^1^ Cancer Center Renmin Hospital of Wuhan University Wuhan China; ^2^ Department of Dermatology, Union Hospital, Tongji Medical College Huazhong University of Science and Technology (HUST) Wuhan China

**Keywords:** breast cancers, FOXK2, FBXO32, pan‐cancer analysis, prognostic analysis

## Abstract

**Objective:**

To search for a new biomarker that can predict the efficacy and prognosis of tumor immunotherapy.

**Method:**

FOXK2 genes were analyzed using single‐cell sequencing in pan‐cancer bulk RNA‐seq from the TCGA database. We used algorithms to predict their immune infiltration. Functional enrichment and ChIP‐seq identified potential downstream gene, FBXO32. FBXO32's role in cancer immune response was explored through analysis.

**Results:**

Significant up‐regulation of FOXK2 was observed in prostate adenocarcinoma (PRAD), uterine corpus endometrial carcinoma (UCEC), bladder urothelial carcinoma (BLCA), colorectal cancer (CRC), pancreatic ductal adenocarcinoma (PDAC), and stomach adenocarcinoma (STAD), while no such increase was found in lung cancer (lung adenocarcinoma [LUAD], lung squamous cell carcinoma [LUSC]) or thyroid carcinoma (THCA) tumor and adjacent tissues. FOXK2 expression correlated with patient prognosis, with lower expression associated with better immune response and survival and higher expression of its downstream gene FBXO32 linked to worse overall survival (OS) and immune infiltration. FOXK2 has the potential to be used as a prognostic indicator and target for treatment in individuals with cancer.

**Conclusion:**

Our research provides insights into the significance of FOXK2 in cancer and indicates its potential as both a prognostic indicator and target for treatment. The ribosome‐associated pathways involving FOXK2 and FBXO32 could be pivotal in the advancement of tumors, offering possible avenues for targeted and individualized immunotherapy approaches. Additional research is required to completely understand the mechanisms that are responsible for the participation of FOXK2 and its subsequent gene FBXO32 in cancer, as well as to explore the possible advantages of focusing on FOXK2 for cancer treatment.

## Graphical abstract

FOXK2 regulates the downstream gene FBXO32 in breast cancer and then activates ribosome‐associated pathways to promote tumor cell proliferation.

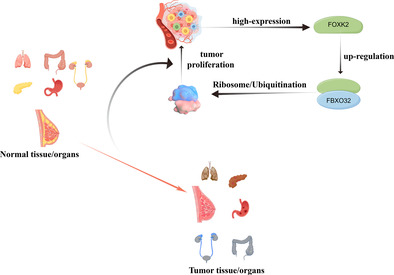

## INTRODUCTION

The tumor microenvironment (TME) is an intricate network consisting of tumor cells and various non‐cancerous elements, such as immune cells, fibroblasts, endothelial cells, and extracellular matrix,^1^, ^2^ all of which are crucial in controlling tumor activity. Immunotherapy is a hopeful strategy for cancer treatment that involves stimulating the immune system to fight cancer, however, only a minority of patients see positive results,^3^ highlighting the necessity for new predictive biomarkers. FOXK2, a member of the FOX family of transcription factors, plays a role in cancer progression, although its specific function in cancer is not fully understood.^4^, ^5^ Our objective in this research was to explore if FOXK2 could function as a prognostic indicator for the effectiveness of immunotherapy in different types of cancer. Due to the small batch effect of the data, complete grouping, and close association with clinical samples, we selected single‐cell data from eight cancer types including prostate adenocarcinoma (PRAD), thyroid carcinoma (THCA), bladder urothelial carcinoma (BLCA), lung cancer, uterine corpus endometrial carcinoma (UCEC), stomach adenocarcinoma (STAD), colorectal cancer (CRC), and pancreatic ductal adenocarcinoma (PDAC) in the GEO database. Examining data from eight cancer types, we discovered that FOXK2 expression levels were notably elevated in tumor cells compared to non‐tumor cells.^6^ In addition, we performed enrichment analysis (including Gene Ontology [GO] and Kyoto Encyclopedia of Genes and Genomes [KEGG]) on genes with differential expression and analyzed the expression of nine checkpoints including vascular endothelial growth factor A (VEGFA), cluster of differentiation 27 (CD27), interleukin‐2 receptor alpha (IL2RA), etc. discovering a significant association between FOXK2 expression and immune cell infiltration, activation of anti‐cancer immunity, and the survival rate of patients undergoing immunotherapy. We also identified the downstream gene FBXO32 of FOXK2 and, for the first time, found through differential expression gene enrichment analysis that it may be related to cellular ribosome‐associated pathways, thereby affecting tumor cell ribosome. This may provide new ideas for tumor treatment. Our study results indicate that FOXK2 probably act as a significant immunotherapy marker that regulates the FOXK2–FBXO32–ribosome‐associated pathways, ultimately participating in tumor initiation, progression, and outlook. This study may provide new insights for personalized cancer treatment.

## MATERIALS AND METHODS

### Information

#### Bulk‐seq data analysis

The pan‐cancer dataset was acquired using the Tumor Immune Estimation Resource version 2.0 (TIMER2.0) web server. We retrieved FOXK2 differential expression data from 33 tumor tissue types and their corresponding normal tissues in the TCGA database,^7^ with a distribution of 57.55% white, 5.32% African, 3.65% Asian, 9.28% other ethnicities, and 24.20% unrecorded. For several types of tumors without paired normal tissues, we use GEPIA2 (http://GEPIA2.cancer-pku.cn/) Network server,^8^ supplementing normal samples from GTEx database. The merged transcripts per million (TPM) data will be analyzed by the R survival 3.5.7 package and then create the overall survival (OS) plots. In order to ensure the authenticity and comprehensiveness of the data, we also collected the CGGA database (http://www.cgga.org.cn/). The sequencing data of 2000 Chinese patients with glioma were combined with the TCGA database data, and the survival prognosis was analyzed after the batch effect was eliminated with the R limma package. The FOXK2 protein's proteomic expression profile can be found on the UALCAN portal at http://UALCAN.path.uab.edu. This data is sourced from the Clinical Proteomic Tumor Analysis Consortium (CPTAC) dataset.^9^ RNA sequencing data obtained for the TCGA pan‐cancer (PANCAN) cohort from the UCSC Xena browser at https://xenabrowser.net/.Protein coding genes are encoded by Ensembl (http://www.Ensembl.org) Annotations. Next, standardize gene expression levels to TPM and then transform them to log2 (TPM + 1); for further analysis, please refer to Supplemental 2 for detailed quality control parameters.

#### ChIP‐seq data analysis

Quality control and trimming of the reads were done using FastQC (v0.11.9) using default parameters and Trimmomatic (v0.39) with parameters (ILLUMINACLIP:TruSeq3‐PE‐2.fa:2:30:10:8 LEADING:3 TRAILING:3 SLIDINGWINDOW:4:15 MINLEN:8) to trim any remaining adapters or bases with low‐quality scores and remove reads shorter than 8nt.^10^ Clean reads were aligned to the human GRCh38 genome using Bowtie2 (v2.4.5)^11^ and the following options “‐X” and “‐‐very‐sensitive.” PCR duplicates were removed using Picard MarkDuplicates. Peak calling was performed using Macs2(v2.1.1)^12^ with parameters “‐f BAMPE ‐B ‐‐SPMR ‐‐keep‐dup all.” Peaks were filtered at a *q*‐value threshold <0.05. Peak calls from each replicate^13^ across both controls and treatments were merged into a union set using bedtools merge.^14^ Peaks were annotated to the nearest feature using ChIPseeker (v1.32.0). GO and KEGG analyses were performed using clusterProfiler (v4.0.0). A consensus motif sequence analysis was performed using HOMER. Normalized ChIP‐seq signals were derived from bigwig files using bedGraphToBigWig and visualized with Integrative Genomics Viewer (v2.8.0).^15^

#### Single‐cell data processing

Sample data was initially read, with cells meeting certain criteria included for analysis. Quality control steps involved filtering out cells with high mitochondrial gene ratios (>10%), red blood cells, cells with nFeature‐RNA <300 or >8000, and doublets. The DoubletFinder R package v2.0.3 was utilized for doublet removal. Following these quality controls, a total of 128 985 cells suitable for further analysis were retained.

Single‐cell data in this article were obtained from the open‐source dataset GSE210347 (https://www.ncbi.nlm.nih.gov/geo/query/acc.cgi?acc=GSE210347). For more detailed information, please refer to supplemental 1.

#### Cell grouping

Cell/group recognition utilized the Seurat R package v4.4.0, following the tutorial from the Seurat website. It was initially attempted with the SingleR R package v1.10.0, but manual annotation was used due to suboptimal clustering results. Top 10 marker genes for 11 cell communities were identified and validated using the Cell Marker2.0 website for clustering determination.

#### The expression study of FOXK2 in pan‐cancer included 33 cancer species and 60 499 cancer patients

The ESTIMATE algorithm can be used to predict the proportion of immune and stromal cells as well as tumor purity in malignant tumor tissue by analyzing expression data,^16^ which can compute immune and stromal scores, and indicate the level of cell infiltration in tumor tissue. The ESTIMATE score has an inverse relationship with tumor purity, which is the percentage of tumor cells in the sample. To further investigate this correlation, we analyzed the connection between FOXK2 expression and immune cells that have infiltrated the tumor using TIMER, xCell,^17^ and quanTIseq^18^, ^19^ algorithms. Using multiple algorithms for analysis, a more comprehensive understanding of infiltration can be obtained, which can reflect the types and proportions of various infiltrating cells more comprehensively.

#### The mutation analysis of FOXK2

Mutations of FOXK2 in pan‐cancer from cBioPortal (http://www.cBioPortal.org/) obtain the mutation frequency, mutation type, and copy number changes (CNA) of TCGA tumor types, as well as the survival curve of patients with FOXK2 gene changes. An analysis was performed to enrich genes and proteins related to FOXK2. Genes showing a strong correlation with FOXK2 (Spearman correlation coefficient absolute value >0.4, *p* < 0.05) were selected for gene annotation enrichment analysis in patients with various types of cancer. Significant differences in GO and KEGG pathways were determined by false discovery rate (FDR) <0.05. Gene lists for each cancer type were sorted based on correlation coefficients and used for genomic enrichment analysis (GSEA) in GO and KEGG databases. A notable finding was characterized by an absolute *p* value less than 0.05, FDR *q* value less than 0.05, and normalized enrichment score (NES) greater than 1.5. The protein–protein interaction network of FOXK2 utilized the Interaction Gene Retrieval Tool (STRING, https://www.STRING-DB.org/).^19^ A confidence level >0.4 is considered significant.

#### Analysis of FOXK2 expression levels and immunotherapy

There are nine immune checkpoints, which include PD‐1 (PDCD1), PD‐L1 (also called CD274), CTLA4, LAG3, TIGIT, TIM‐3 (HAVCR2), VISTA (C10orf54), and B7‐H3 (CD276), including B and T cell lymphocyte attenuators, known as BTLA. Tumor mutational burden (TMB) is determined by the sum of non‐synonymous mutations per million genome bases and is computed using the R software tool “TCGA mutations.”^20^

Therapeutic response prediction in individuals with varying FOXK2 expression levels is determined by the Tumor Immune Dysfunction and Exclusion (TIDE) score and Immune Phenotype Score (IPS).^21^ The TIDE score of pan‐cancer patients is obtained from the TIDE network platform (http://TIDE.dfci.harvard.edu/). A TIDE score threshold of 0 was established, classifying patients with negative scores as responders. Generally speaking, patients with lower TIDE scores and higher IPS have a better response to immunotherapy.

#### Analysis of FOXK2 expression level and drug sensitivity test

For drug sensitivity test analysis, we used pRRopic R package v 0.5 for calculation. Please refer to details for reference (https://github.com/satijalab/seurat/pull/5392).

#### Enrichment analysis of FBXO32

Patients were categorized into groups with high or low expression levels of FBXO32 in the TCGA database. DESeq2^13^ was utilized for comparative analysis to detect genes with differential expression, subsequently conducting GO and KEGG enrichment analyses to investigate underlying pathways. The implementation of enrichment analysis relies on the R clusterProfiler package for analysis, and the selection criteria for differentially expressed genes (DEGs) are set to a parameter threshold *p*‐value of 0.05. The top 100 genes with upregulated and downregulated log2fold changes were selected, totaling 200 genes, for enrichment analysis.

## RESULTS

### Expression of FOXK2 in pan‐cancer

**FIGURE 1:**
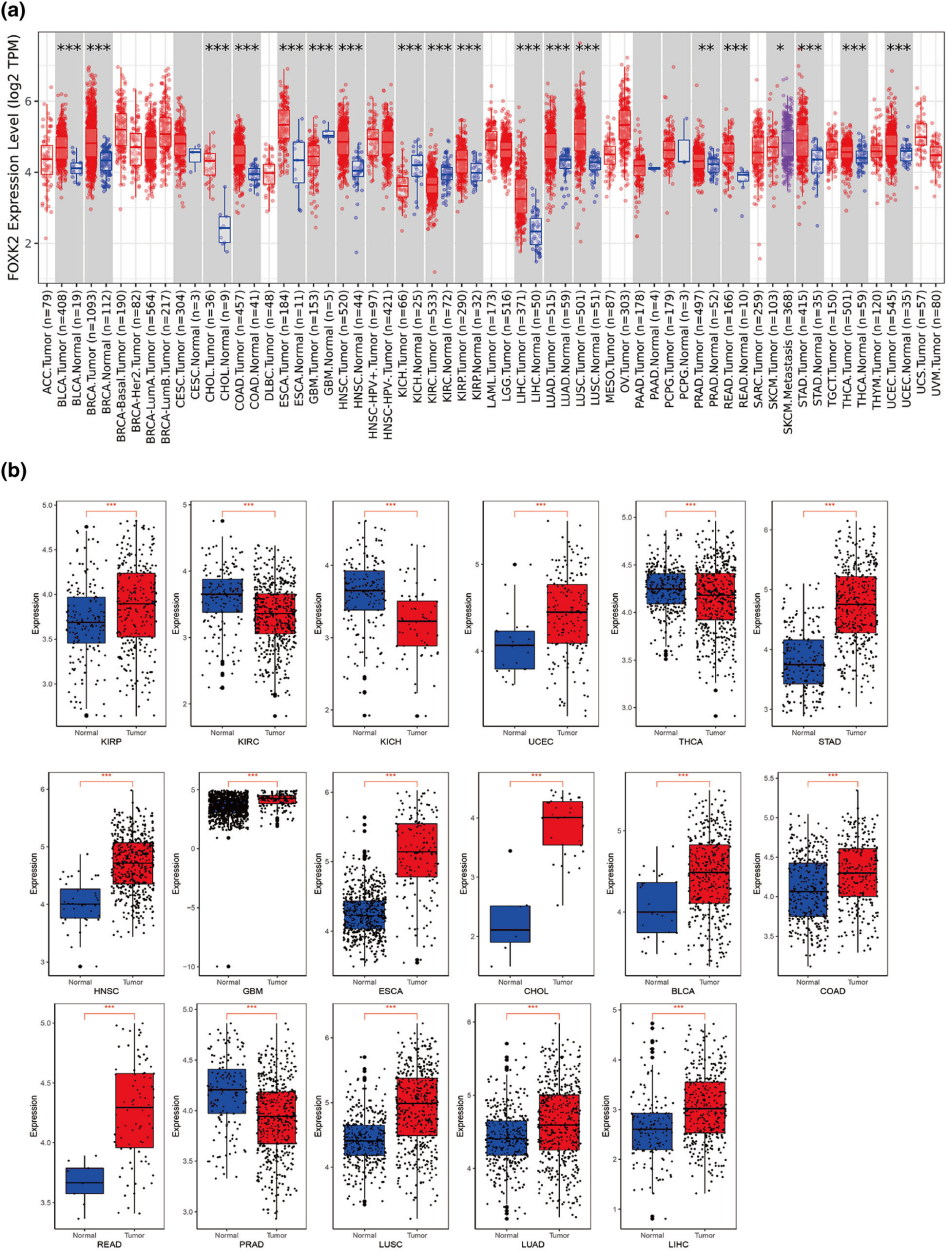
Expression levels and correlation of FOXK2 gene and protein in different tumor tissues. (a) The expression levels of FOXK2 in normal tissues and 33 tumor types or specific subtypes of tumors obtained from TIMER2.0 in the TCGA database (*p < 0.05; **p < 0.01; ***p < 0.001). (b) Compared with normal samples in the TCGA database, KIRP, UCEC, STAD, HNSC, GBM, ESCA, CHOL, BLCA, COAD, READ, LUSC, LUAD, LIHC, KICH, KIRC, THCA, and PRAD cancer type samples in TCGA and GTEx. BLCA, bladder urothelial carcinoma; CHOL, cholangiocarcinoma; COAD, colon adenocarcinoma; ESCA, esophageal carcinoma; GBM, glioblastoma multiforme; HNSC, head and neck squamous cell carcinoma; KICH, renal chromophobe; KIRC, renal clear cell carcinoma; KIRP, kidney renal papillary; LIHC, liver hepatocellular carcinoma; LUAD, lung adenocarcinoma; LUSC, lung squamous cell carcinoma; PRAD, prostate adenocarcinoma; READ, rectum adenocarcinoma; STAD, stomach adenocarcinoma; THCA, thyroid carcinoma; UCEC, uterine corpus endometrial carcinoma.

In the TCGA database, FOXK2 showed decreased expression in tumor samples of glioblastoma multiforme (GBM), renal chromophobe (KICH), and renal clear cell carcinoma (KIRC) compared to the normal control group (*p* < 0.05) among the 33 cancer types. In contrast, FOXK2 is linked to BLCA (*p* < 0.05), breast invasive carcinoma (BRCA) (*p* < 0.05), cholangiocarcinoma (CHOL) (*p* < 0.05), colon adenocarcinoma (COAD) (*p* < 0.05), esophageal carcinoma (ESCA) (*p* < 0.05), and head and neck squamous cell carcinoma (head and neck squamous cell carcinoma). Tumor samples exhibited elevated expression in head and neck squamous cell carcinoma (HNSC) (*p* < 0.05), kidney renal papillary cell carcinoma (KIRP) (*p* < 0.05), liver hepatocellular carcinoma (LIHC) (*p* < 0.05), lung adenocarcinoma (LUAD) (*p* < 0.05), lung squamous cell carcinoma (LUSC) (*p* < 0.05), prostate adenocarcinoma (PRAD) (*p* < 0.05), rectum adenocarcinoma (READ) (*p* < 0.05), STAD (*p* < 0.05), thyroid carcinoma (THCA) (*p* < 0.05), and uterine corpus endometrial carcinoma (UCEC) (*p* < 0.05). In addition, compared with the primary tumor sample, the expression of FOXK2 was upregulated in the metastatic sample of skin melanoma (SKCM) (*p* < 0.05) (Figure 1a). Next, we matched the cancer types with significant differences in FOXK2 expression to normal organizations in the TCGA and GTEx databases. The study findings indicated a highly notable variance in FOXK2 expression levels between tumor and normal tissues across 17 types of cancer (*p* < 0.05) (Figure 1b).

### Expression of FOX family genes in pan‐cancer single‐cell sequencing samples

**FIGURE 2:**
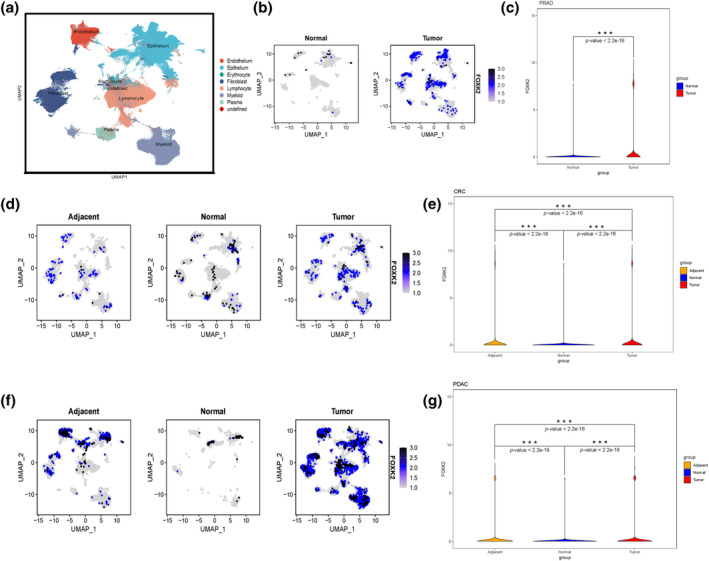
Single‐cell expression of FOXK2 in different tumors. (a) UMAP method displays single‐cell profiles from 10 cancer species, with cell types in order: endothelial cells, epithelial cells, red blood cells, fibroblasts, lymphocytes, myeloid cells, plasma (including B cells, T cells, etc.), and undefined cells. (b) The expression of FOXK2 in prostate adenocarcinoma (PRAD) tissue and normal tissue. (c) Differential analysis of FOXK2 between PRAD tissue and normal tissue using *t*‐test method. (d) The expression of FOXK2 in colorectal tumor tissue, adjacent cancerous tissue, and normal tissue. (e) Differential analysis of FOXK2 in colorectal tumor tissue, adjacent cancer tissue, and normal tissue using *t*‐test method. (f) The expression of FOXK2 in pancreatic tumor tissue, adjacent cancerous tissue, and normal tissue. (g) Differential expression analysis of FOXK2 in pancreatic tumor tissue, adjacent cancer tissue, and normal tissue using *t*‐test method.

After quality control processing and dimensionality reduction clustering of the data, eight cell clusters were identified: endothelial cells, epithelial cells, red blood cells, fibroblasts, lymphocytes, myeloid cells, plasma cells, and undefined (Figure 2a). The analysis of integrated single‐cell data by cancer type revealed significant upregulation of FOXK2 in all cancer types compared to normal tissue (Figures 2b–g and S1). Specifically, FOXK2 was found to be significantly overexpressed in tumor tissues of prostate adenocarcinoma (PRAD), endometrial cancer (UCEC), BLCA, CRC, pancreatic duct adenocarcinoma (PDAC), STAD, lung cancer, and thyroid cancer. These findings suggest that FOXK2 is highly expressed in pan‐cancer tumor tissues, particularly in fibroblasts and epithelial cells.

### Survival analysis of FOXK2

In pan‐cancer, the high expression of FOXK2 indicates that patients have poor prognosis (Figure S2a,b) in terms of OS (*p* < 0.001) and disease‐free survival (DFS) (*p* < 0.001). Furthermore, we chose various types of tumors with a high frequency of occurrence to investigate the relationship between FOXK2 levels and overall survival. Patients with elevated levels of FOXK2 in adrenocortical carcinoma (ACC), KICH, BLCA, and uveal melanoma (UVM) have a worse prognosis than those with lower levels, as shown by both OS (*p* < 0.001) and DFS (*p* < 0.001) (Figure S2c–g), suggesting a negative outlook for tumor patients with high FOXK2 expression.

### The presence of FOXK2 is linked to the infiltration of immune cells in the tumor micro‐environment

**FIGURE 3:**
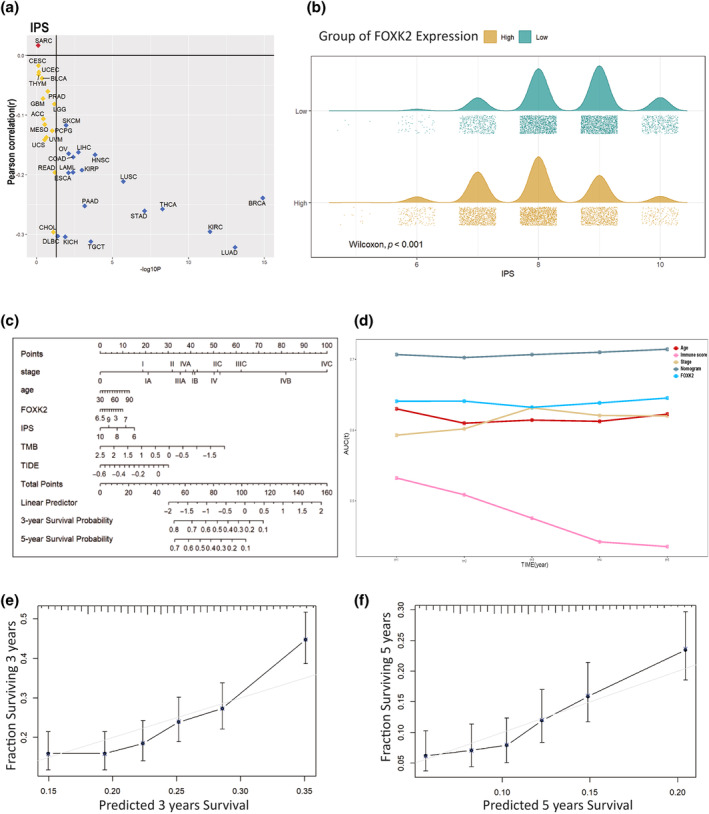
Predicting the immunotherapy efficacy of patients with different FOXK2 expressions. (a) Correlation between FOXK2 and Immune Phenotype Score (IPS) score in 33 cancer patients. (b) Distribution of IPS scores in high and low FOXK2 expression groups. (c) Nomogram plot for predicting immunotherapy efficacy based on age, stage, FOXK2, TIDE score, IPS score, and TMB. (d) AUC evaluation includes the predictive performance of five models: nomogram, FOXK2, age, staging, and immune score. (e) Predicted 3‐year survival rate and actual survival rate. (f) Predicted 5‐year survival rate versus actual survival rate.

In our study, we used ESTIMATE, TIMER, xCell, and quanTIseq algorithms to evaluate the infiltration level of immune cell and stromal cell in tumor tissues (Figure S3a–d). The current mainstream view is that quantTIseq is the only method that provides an “absolute fraction” representing cell fractions, and xCell has an advantage in calculating the presence or absence of cell types. TIMER and cibersort are used together as supplements to the prediction results. Among them, in the xCell algorithm, the phenomenon of increased FOXK2 expression leading to reduced immune infiltration is more prominent (Figure S3a). Compared with the low group, the high FOXK2 expression group showed varying degrees of reduction in the infiltration of all immune cells except CD4 + Th2 cells and cancer associated fibroblasts (CAFs). The quanTIseq algorithm also calculated that in cancer, the T cell infiltration in the FOXK2 high expression group was significantly lower than that in the low expression group. In our study (Figure S3c), we utilized ESTIMATE, TIMER, xCell, and quanTIseq algorithms to evaluate immune cell infiltration, stromal cell levels, and tumor purity in tumor tissues. In most types of cancer, we found a correlation between increased FOXK2 expression and decreased immune score, indicating reduced immune cell infiltration (Figure 3a–c). In addition, tumors with up‐regulated FOXK2 expression often have lower tumor purity, which may be due to an increase in CAFs in the tumor microenvironment (Figure S3d).

### Changes in FOXK2 in pan‐cancer

According to the analysis of TCGA data set, the frequency of FOXK2 changes in melanoma patients is the highest (>6%), and the mutation rate of breast cancer also ranks in the top six (>4%) (Figure S4a). Among all mutations in FOXK2, missense is the most frequent mutation mode, appearing a total of 79 times (Figure S4b). The OS (*t*‐test, *p* = 0.0336) and PFS (*t*‐test, *p* = 0.0117) of invasive cancer patients with FOXK2 mutations were not significantly different from those without FOXK2 gene mutations, which may be due to the relatively small number of mutated samples compared to non‐mutated samples (Figure S4c,d).

### Analysis of gene and protein enrichment related to FOXK2

We divided the TCGA dataset into high and low expression groups based on the median TPM expression of FOXK2 and screened 200 FOXK2‐related DEGs to investigate their potential functions in pan‐cancer patients. Our GO and KEGG pathway analysis results showed that there are numerous histone modifications, mRNA processing, and protein processing in the endoplasmic reticulum (Figure S5a). KEGG pathway analysis also showed that FOXK2‐related genes are involved in protein processing in the endoplasmic reticulum (Figure S5b). Further exploration through GSEA enrichment analysis showed that FOXK2 was significantly enriched in the “BRCA2 TARGET GENES” gene set (Figure S5c). In addition, the protein interaction network of FOXK2 includes key proteins involved in pathways related to tumor occurrence and development, indicating its involvement in affecting endoplasmic reticulum protein processing (Figure S5d).

### The correlation between the expression of FOXK2 and immune checkpoint gene expression

To determine the potential role of FOXK2 expression in tumor immunotherapy, we investigated the correlation between FOXK2 expression and some biomarkers in 33 cancer types. Among the nine immune checkpoint related genes, excluding cancer types that did not have statistical significance (*p* > 0.05), the expression of CD276, HMGB1, and BTN3A1 in tumors was positively correlated with FOXK2 expression (Figure S6c,d,h); VTCN1, IL2RA, ICOSLG, ICAM1, BTN3A1, CD27, and FOXK2 expressions were positively correlated in some cancer types and negatively correlated in others (Figure S6a,b,e–g,i). Among them, only CD27 was negatively correlated with FOXK2 expression in BRCA, while the other immune checkpoint genes were positively correlated with FOXK2 expression (Figure S6f).

### Predicting the response to immunotherapy in patients with varying levels of FOXK2 expression

To predict the effectiveness of immunotherapy, we evaluated the IPS scores of patients with different types of cancer. In all types of cancer with a significance level of *p* < 0.05, FOXK2 expression was negatively correlated with IPS score (Figure 3a). It can be clearly seen from the mountain chart that the average and peak IPS scores of samples with high FOXK2 expression are significantly lower than those of samples with low FOXK2 expression (Figure 3b). Subsequently, we considered widely accepted tumor immunotherapy predictive indicators such as staging, age, IPS score, TIDE score, and TMB, and here we added FOXK2 expression levels. We selected 2500 pan‐cancer samples from the TCGA database and integrated their clinical information and expression levels for screening, leaving 1500 cases. Subsequently, 200 samples were selected using random sampling method, and a model was established through multiple regression analysis to create a column chart (Figure 3c). Next, we performed area under curve (AUC) on the model and other independent variables, and it is not difficult to find that the nomogram model we established has the best predictive performance (Figure 3d). We selected the remaining 1300 samples to evaluate the accuracy and consistency of the previously constructed model. The results showed that the model exhibited good predictive performance for patient survival curves over a 3‐ and 5‐year time span (Figure 3e,f).

### Verification of predictive ability of FOXK2 expression in drug sensitivity testing and immunotherapy

**FIGURE 4:**
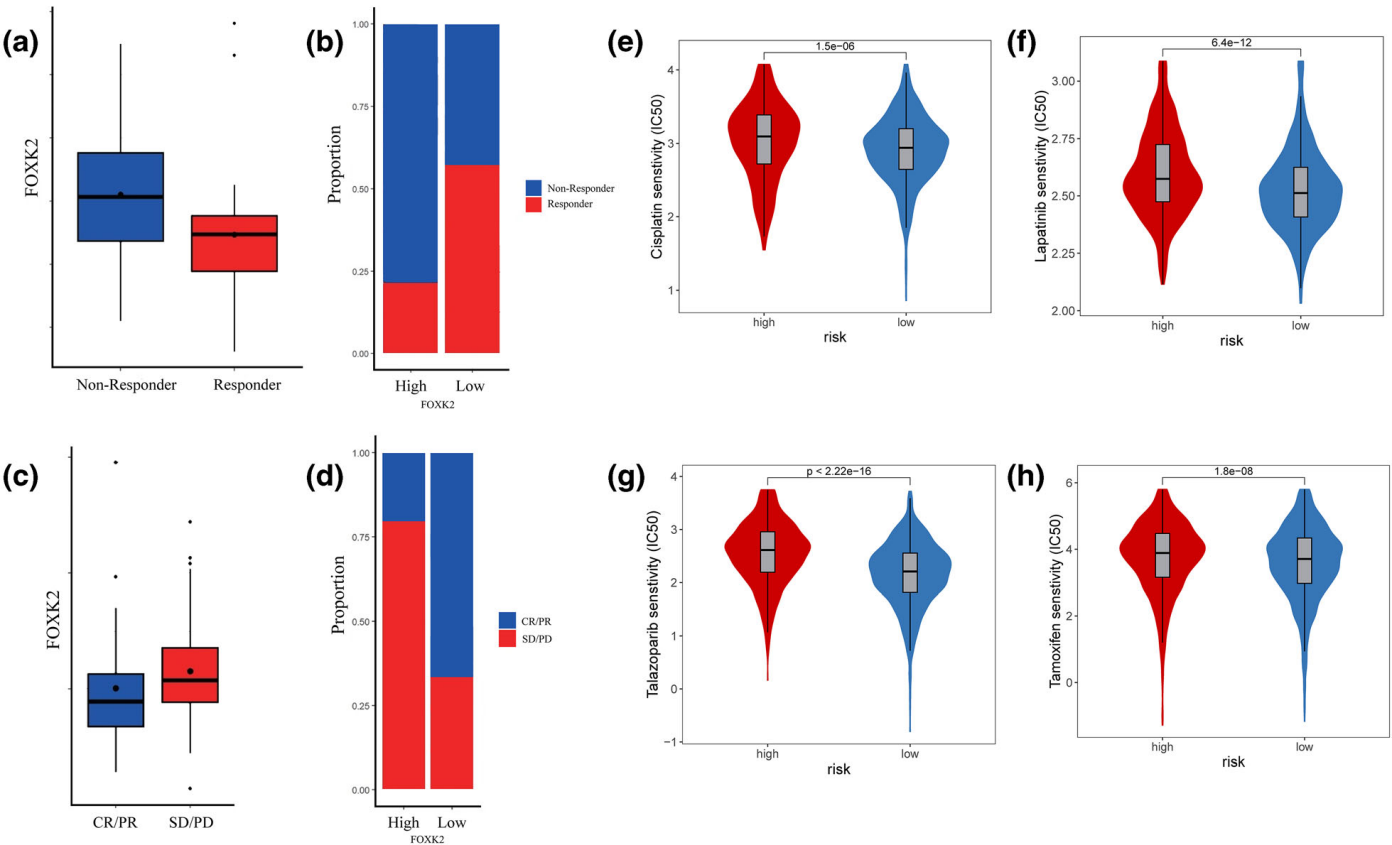
Validation of FOXK2 expression, prediction of immunotherapy, and drug sensitivity testing the *x*‐axis represents the high and low groups of TPM median scores obtained by dividing them into high and low groups using FOXK2 expression among 33 cancer types in the TCGA database. The *y*‐axis represents the calculated immune scores, and the violin box plot shows the average score of this group. (a) Expression of FOXK2 in different immunotherapy response groups in the Ulloa Montoya cohort data. (b) Immunotherapy efficacy (response rate) of different FOXK2 expression groups in the Ulloa Montoya cohort data. (c) Expression of FOXK2 in different immunotherapy response groups in Riaz queue data. (d) Immunotherapy efficacy (response rate) of different FOXK2 expression groups in Riaz queue data. (e–h) Sensitivity testing of FOXK2 in drugs such as cisplatin, lapatinib, talazoparib, and tamoxifen.

In the metastatic melanoma immune therapy cohort study by Ulloa‐Montoya et al.^22^ (GSE35640), patients with higher levels of FOXK2 expression showed poorer immunotherapy response compared to those with lower expression levels (Wilcoxon test, *p* < 0.05) (Figure 4a,b). Similar findings were observed by Riaz et al. (GSE91061)^23^ in melanoma and non‐small cell lung cancer cohorts, where patients with complete or partial remission showed lower FOXK2 expression compared to those with disease stabilization or progression after immunotherapy (Figure 4c,d). Moreover, upon scrutinizing the TCGA database, it was discerned that individuals manifesting elevated levels of FOXK2 expression displayed enhanced responsiveness to conventional breast cancer therapeutics, including cisplatin, lapatinib, talazoparib, and tamoxifen (Figure 4e–h). Additionally, heightened sensitivities to pharmaceutical agents such as dabrafenib, dasatinib, erlotinib, foretinib, saracatinib, and trametinib were observed in this cohort (Figure S7), as evidenced by statistically significant findings (*p* < 0.001, *t*‐test).

### Exploration of downstream genes of transcription factor FOXK2

**FIGURE 5:**
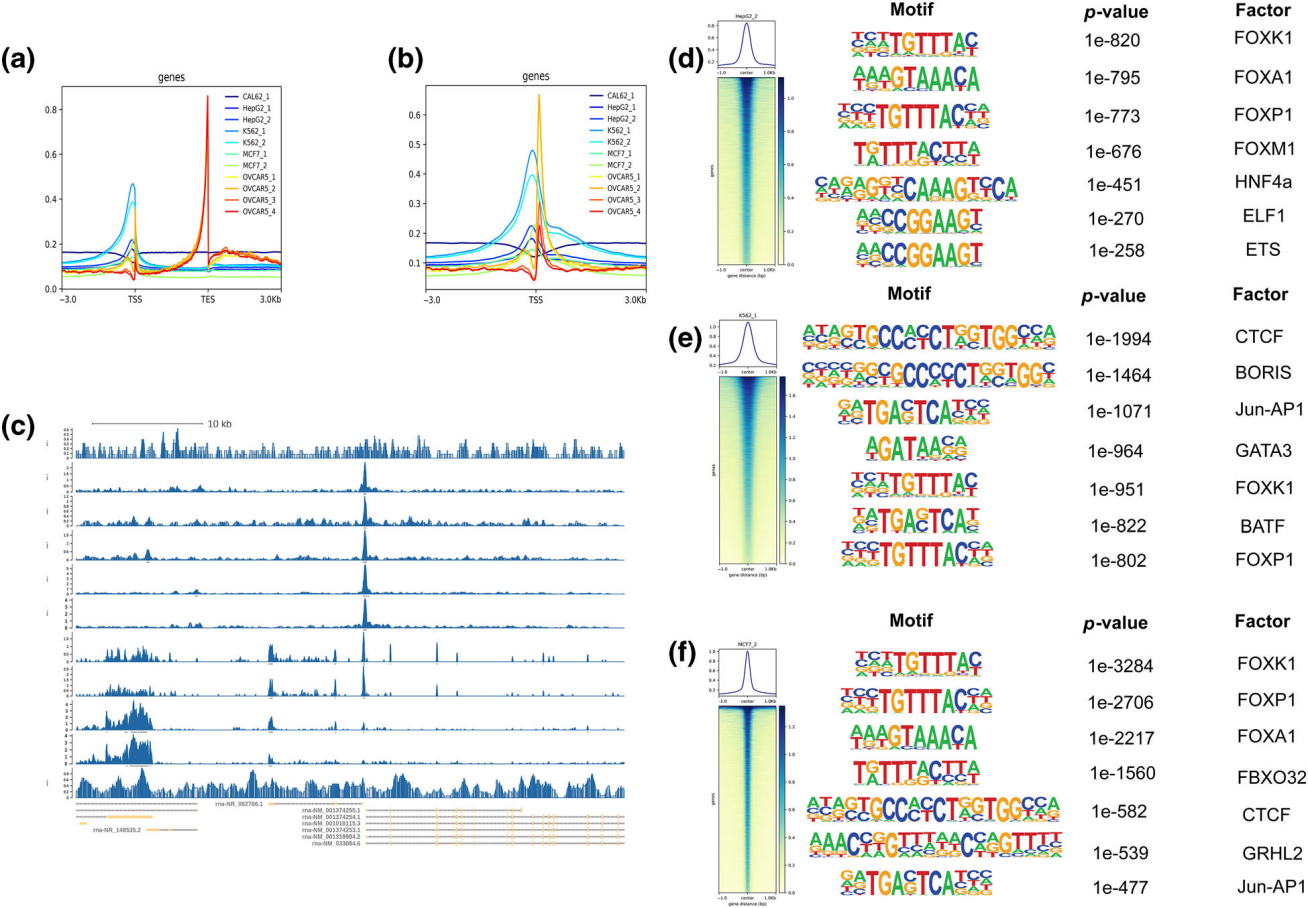
Searching for downstream genes of FOXK2 ChIP‐seq. (a) TSS (transition start site) peak plots for five types of cancer cell line data. (b) TES (transition end site) peak plots for five types of cancer data. (c) Prediction of ChIP‐seq binding sites in five cancer cell line samples. (d) Potential downstream gene motif prediction in HepG2. (e) Potential downstream gene motif prediction in K562. (f) Potential downstream gene motif prediction in MCF‐7.

We obtained ChIP‐seq data of FOXK2 in different cancer species from four datasets: GSE84241(BRCA, MCF‐7), GSE91547(LIHC, HepG2), GSE91647(CML, K562), GSE173780(OV, OVCAR5), and GSE165777(thyroid cancer, CAL‐62). After analysis, it was found that almost all cancer types had peaks in the TSS (Transition Start Site) segment, but only OV had peaks in the TES (Transition End Site) segment (Figure 5a–c). Next, we selected cell lines with high prediction accuracy (HepG2, K562, MCF7) and listed the first seven prediction genes (Figure 5d–f). We found that FBXO32 is the most likely downstream gene in the breast cancer cell line MCF‐7, except for the FOX family (Figure 5f).

### Enrichment of FOXK2 downstream genes among different cell lines

**FIGURE 6:**
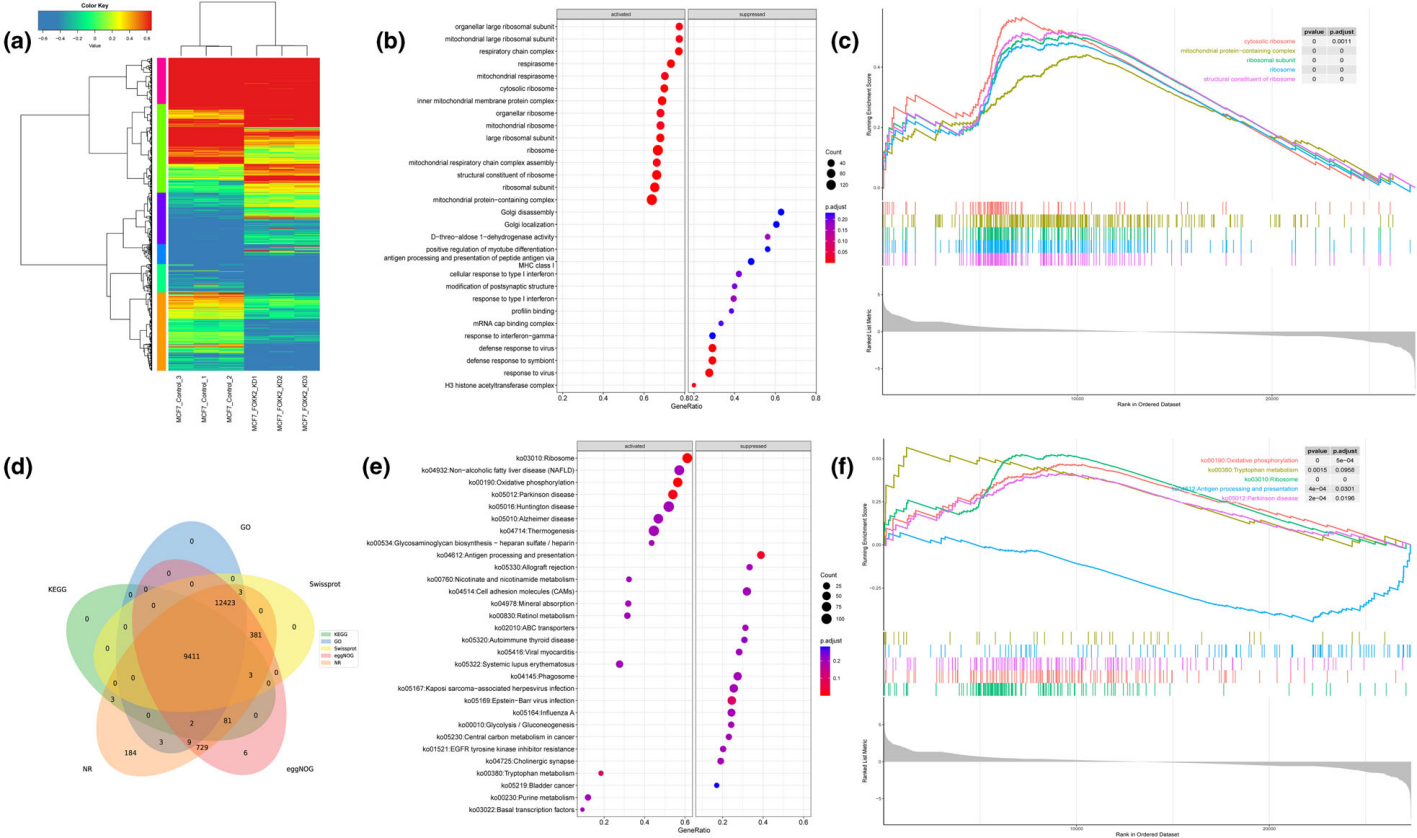
Enrichment analysis of downstream genes of FOXK2 in MCF‐7 cell lines. (a) Gene expression heatmap clustering analysis of normal and FOXK2 knockout groups in MCF7 cell lines. (b) Perform GO enrichment analysis on differentially expressed genes. (c) Perform GSEA analysis on GO enrichment analysis. (d) Venn analysis showed upregulation in MCF7 cell lines. (e) Perform KEGG enrichment analysis on differentially expressed genes. (f) Perform GSEA analysis on KEGG enrichment analysis.

We continued to explore MCF7 cell lines with better expression and conducted expression clustering analysis on three experimental and control groups of MCF7 cell lines. We found that there was a significant expression difference between FOXK2 knockout cells and the control group cells (Figure 6a). Following identification of genes with differential expression in two cell groups, we conducted GO and GSEA enrichment analyses (Figure 6b,c) and observed that the absence of FOXK2 notably activated pathways related to ribosome‐associated pathways, a finding supported by KEGG and GSEA enrichment analyses (Figure 6e,f). Of course, we also repeated the above operation in the K562 cell line (Figure S8). Enriched pathways in the K562 cell line that knock out FOXK2 included mitotic pathways, in contrast to the control group.

### Screening for possible downstream target genes of FOXK2

We screened based on the enrichment results of downstream genes of FOXK2 and selected genes that intersected with GO enrichment analysis and KEGG enrichment analysis pathways (Figures 6d and S8d). We validated the genes with *p* < 0.05 and log2FoldChange >2 in the TCGA database (Figure S9). Finally, it was found that FBXO32 was the gene with the strongest correlation and most significant difference with tumor OS (Figure S10e).

### Exploration of FBXO32 regulatory pathway

**FIGURE 7:**
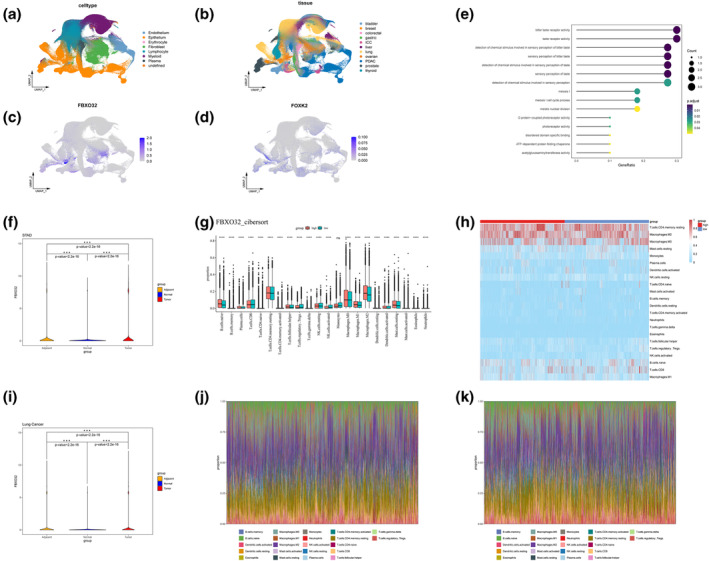
Expression, enrichment analysis, and immune infiltration of FBXO32 in pan‐cancer. (a) UMAP method displays single‐cell profiles from eight cell sources. (b) Display single‐cell atlases from 10 cancer species and normal tissues using UMAP method. (c) Characteristic map of FBXO32 expression in single cells. (d) Characteristic map of FOXK2 expression in single cells. (e) The GO enrichment analysis results of dividing patients in the TCGA database into high and low groups based on FBXO32 expression levels for differential analysis. (f) Differential analysis of FBXO32 in gastric tumor tissue, adjacent cancer tissue, and normal tissue using t‐test method. Immune infiltration analysis of FBXO32 (g, h) in TCGA database pan‐cancer using cibersort method. (i) Differential analysis of FBXO32 in lung tumor tissue, adjacent cancer tissue, and normal tissue using *t*‐test method. (j) Immune infiltration stacking map of patients with high expression of FBXO32, using cibersort method. (k) Immune infiltration stacking map of patients with low FBXO32 expression, CIBERSORT method.

To confirm the co‐expression of FBXO32 and FOXK2, we clustered and grouped the single‐cell data from GSE210347 to generate a new UMAP visualization (Figure 7a,b). Next, we conducted feature plots of FOXK2 and FBXO32 in single‐cell samples to explore their expression distribution in cells (Figure 7c,d). The results showed that FBXO32 and FOXK2 were significantly co‐expressed in epithelial cells of lung cancer STAD, and BLCA (Figure S10f).

Subsequently, we used the median expression level of bulk RNA seq as a threshold to divide patients in the TCGA database into FBXO32 high expression group and low expression group. DESeq2 package was used to conduct differential analysis on these groups to screen genes for GO enrichment analysis (Figure 7b–f). We utilized cibersort, ESTIMATE, and xCell techniques to assess immune infiltration in the high and low groups (Figures 7g,h–k and S10a–d); all technical parameters and version information are consistent with the aforementioned method. According to the above analysis, except for monocytes, the score and proportion of FBXO32 high expression group have changed (*p* < 0.05, *t*‐test) (Figure 7g).

## DISCUSSION

The exact role of FOXK2 in biology is not yet clear, but increasing research suggests that FOXK2 is crucial for cancer prognosis, possibly because it can regulate cancer cells^24^ and CAFs,^25^ affecting the tumor immune microenvironment. The specific functional connections may be the regulation of aerobic glycolysis^26^ and the regulation of cellular autophagy pathways,^27^ as well as the ribosome‐associated pathways discovered in previous studies. Nevertheless, there is a scarcity of comprehensive studies examining how FOXK2 affects the effectiveness of treatment across various types of cancer and alterations in the immune environment within tumors. This research involved a thorough examination of the FOXK2 expression levels across 33 different types of cancer in the TCGA database, suggesting FOXK2 as a potential biomarker for predicting response to immunotherapy.

Pan‐cancer analysis indicates that FOXK2 is differentially expressed between tumor and normal tissues. The high expression of FOXK2 in ACC, BLCA, KICH, and UVM is associated with poor prognosis. Previous studies have indicated that FOXK2 is significant in the proliferation and metastasis of tumors, as well as in the development of drug resistance through epithelial mesenchymal transition (EMT).^28^, ^29^ FOXK2 may promote cancer cell proliferation by activating Wnt‐signaling through interaction with DVL (DVL is a stable activator of β‐catenin).^30^ The analysis of patient samples shows that high FOXK2 expression is related to tumor proliferation of breast cancer and ACC.^31^ The presence of FOXK2 is linked to the metastasis of colorectal cancer, and the drug cetuximab can disrupt the feedback loop involving EGF NF‐κB–FOXK2–EGFR, thus preventing metastasis in CRC.^32^ Of course, there are also different opinions; some people believe that high FOXK2 expression can inhibit tumor EMT and proliferation,^29^ but this may due to the heterogeneity between tumors.

For example, this article starts with pan‐cancer and finally focuses on FOXK2 and breast cancer related prognosis. It is found that FOXK2 may play a vital role as a transcription factor and ultimately regulate downstream genes and pathways, leading to poor prognosis of tumors. In addition, high FOXK2 expression activates VEGFA, leading to anaplastic thyroid cancer via the VEGFA/VEGFR1 pathway. It is not difficult to find that the role of FOXK2 may not be completely the same among different tumors.

Based on our prior findings from immune infiltration analysis, a plausible explanation for this phenomenon could be attributed to alterations in tumor microenvironment components (e.g., CAFs), influencing the expression microenvironment of FOXK2 across distinct tumor types. This study reveals FOXK2's correlation with the tumor microenvironment in pan‐cancer. Tumors with high FOXK2 show lower purity and increased stromal and immune cell infiltration. FOXK2 is highly expressed in chronic lymphocytic leukemia (CLL), metastatic breast cancer (MBC), and osteosarcoma clusters and corresponds to poor outcomes in melanoma and non‐small cell lung cancer (NSCLC) cohorts. In the context of BRCA, elevated levels of FOXK2 correlate with increased numbers of cancer‐associated fibroblasts (CAFs) and heightened infiltration of CD4 + TH2 cells, thereby indicating a poorer prognosis. This association could potentially contribute to tumor advancement and the development of drug resistance.^31^, ^33^, ^34^, ^35^ Overall, low expression of FOXK2 may indicate better prognosis and better immunotherapy efficacy. Based on the analysis of two immunotherapy cohorts and the negative correlation between FOXK2 and the expression of nine immune checkpoint genes in most cancer species, our hypothesis is further validated that low expression of FOXK2 indicates better immunotherapy efficacy.

In terms of clinical treatment, FOXK2 may mediate apatinib resistance^36^ or serve as a mediator of paclitaxel resistance in human ovarian cancer cells^33^ and breast cancer cells.^37^ The correlation between the expression level of FOXK2 and the chemoradiotherapy resistance and prognosis of locally advanced cancer patients after neoadjuvant chemotherapy has been validated.^34^ It is noteworthy to highlight that our previous drug sensitivity analysis revealed the absence of several small molecule inhibitors, such as dabrafenib, in the clinical practice guidelines for BRCA. Nevertheless, the predictive outcomes suggest that individuals exhibiting elevated FOXK2 expression levels may demonstrate heightened sensitivity to these drugs.

As a potential downstream target gene for FOXK2, FBXO32 has been found to exhibit both cancer‐causing and anti‐cancer properties in different types of cancer. Evidence suggests that FBXO32 is significantly involved in the progression of different types of cancers, such as pancreatic cancer and lung adenocarcinoma.^38^, ^39^ Its specific mechanisms include promoting ubiquitination to promote tumor growth and migration, as well as regulating the cell cycle to promote tumor growth and metastasis.

This study emphasizes the predictive value of FOXK2 expression in immunotherapy and investigates the FBXO32 gene and its related ribosome‐associated pathways regulated by FOXK2. However, distinguishing between single and combination immunotherapy in cohort validation is challenging. In future research, it is necessary to further investigate the functions of FOXK2 and FBXO32 in cancer cells and the tumor environment. Overall, our pan‐cancer analysis showed that FOXK2 expression was negatively correlated with prognosis in some different cancer types during standard radiotherapy, chemotherapy, and immunotherapy. Among these cancer types, we specifically focused on BRCA, which exhibited the most significant differences in overall survival (OS), and further exploration revealed a potential association with the FOXK2–FBXO32 ribosomal axis. FOXK2 holds promise as a biomarker for predicting immunotherapy responses in BRCA, offering the potential to assist in patient selection and refinement of cancer treatment strategies.

## AUTHOR CONTRIBUTIONS

Fuben Liao contributed to the original draft and review of the writing, as well as the conceptualization. Jinjin Zhu contributed to data curation, investigation, resources. Junju He: validation, investigation, supervision. Qibin Song contributed to writing—review and editing, supervision, funding acquisition. Yi Yao contributed to writing—review and editing, supervision, funding acquisition, project administration. Zheming Liu contributed to reviewing and editing.

## CONFLICT OF INTEREST STATEMENT

The authors declare no conflicts of interest.

## Supporting information


**Data S1.** Supporting Information.

**Figure S1.** Single‐cell expression of FOXK2 in other types of cancer. (a) Distribution in gastric cancer; (b) differential analysis of expression in gastric cancer; (c) distribution in thyroid cancer; (d) differential analysis of expression in thyroid cancer; (e) the distribution in bladder cancer; (f) differential analysis of expression in bladder cancer; (g) distribution in lung cancer; (h) differential analysis of expression in lung cancer; (i) distribution in endometrial cancer; (j) differential analysis of expression in endometrial cancer.

**Figure S2.** OS (overall survival) and DFS (disease‐free survival) analysis of FOXK2. (A) OS of FOXK2 in pan‐cancer (33 types of cancer in the TCGA database); (B) DFS of FOXK2 in pan‐cancer; (C) OS of FOXK2 in ACC; (D) DFS of FOXK2 in ACC; (E) OS of FOXK2 in KICH; (F) DFS of FOXK2 in BLCA; (G) DFS of FOXK2 in UVM.

**Figure S3.** Correlation between FOXK2 expression and immune cell infiltration. (A) Correlation between FOXK2 expression and ESTIMATE Score; (B) the correlation between FOXK2 expression and Stromal Score; (C) the correlation between FOXK2 expression and Immune Score; (D) heat maps of FOXK2 expression and immune infiltration levels were performed using quanTIseq, TIMER, and xCell algorithms, respectively.

**Figure S4.** Mutations of FOXK2 in different tumor tissues. (A) Frequency and range of FOXK2 mutations; (B) mutation site of FOXK2; (C) the location of the most frequent mutation site (X304 splice) on the 3D structure; (D) Kaplan–Meier curves of OS and PFS in UCEC patients with and without FOXK2 mutations.

**Figure S5.** Enrichment analysis of FOXK2‐related genes and proteins. (A) The top 10 GO enrichment significance terms of FOXK2‐related genes in the three functional groups of biological process (BP), cell composition (CC), and molecular function (MF); (B) KEGG pathway analysis of FOXK2‐related genes; (C) GSEA enrichment analysis of FOXK2‐related genes; (D) Foxk2 PPI network created using string tools. Each node represents all proteins generated by a single protein coding gene locus, and each edge represents the predicted functional association.

**Figure S6.** Correlation between FOXK2 and immune checkpoint expression levels. Among 33 types of cancer, FOXK2 is correlated with nine immune checkpoints, with Pearson correlation coefficient on the vertical axis and − log10P. (A) VITCN1 on the horizontal axis; (B) ICOSLG; (C) CD276; (D) VEGFA; (E) ICAM1; (F) CD27; (G) IL2RA; (H) HMGB1; and (I) BTN3A1.

**Figure S7.** Validation of FOXK2 expression, prediction of immunotherapy, and drug sensitivity testing. (A) Sensitivity testing of FOXK2 in dabrafenib; (B) sensitivity testing of FOXK2 in dasatinib; (C) sensitivity testing of FOXK2 in erlotinib; (D) sensitivity testing of FOXK2 in foretinib; (E) sensitivity testing of FOXK2 in saracatinib; (F) sensitivity testing of FOXK2 in trametinib.

**Figure S8.** Enrichment analysis of downstream genes of FOXK2 in K562 cell line. (A) Gene expression heatmap clustering analysis of normal and FOXK2 knockout groups in K562 cell line; (B) perform GO enrichment analysis on differentially expressed genes; (C) perform GSEA analysis on GO enrichment analysis; (D) Venn analysis showed upregulation in K562 cell lines; (E) perform KEGG enrichment analysis on differentially expressed genes; (F) perform GSEA analysis on KEGG enrichment analysis.

**Figure S9.** OS of downstream genes of FOXK2. (A) The OS of BCAS3 in pan‐cancer; (B) the OS of ETV5 in pan‐cancer; (C) the OS of NKAP in pan‐cancer; (D) the OS of FBXO32 in pan‐cancer; (E) the OS of RAI2 in pan‐cancer; (F) the OS of SAMD9 in pan‐cancer; (G) the OS of TBX15 in pan‐cancer; (H) the OS of DNAH9 in pan‐cancer; (I) the OS of CDK15 in pan‐cancer; (J) the OS of CRISP3 in pan‐cancer; (K) the OS of CXCL1 in pan‐cancer; (L) the OS of FER1L6 in pan‐cancer.

**Figure S10.** Expression, enrichment analysis, and immune infiltration of FBXO32 in pan‐cancer. (A, D) Immune infiltration analysis of FBXO32 in pan‐cancer using xCell method; (B, C) immune infiltration analysis of FBXO32 in pan‐cancer using estimate method; (E) the OS of FBXO32, NKAP, and BCAS3 in pan‐cancer; (F) differential analysis of FBXO32 in bladder cancer, endometrial cancer, and thyroid cancer.

## Data Availability

All the single cell expression data can be obtained from the Gene Expression Omnibus (GSE210347), and bulk RNA‐seq data can be obtained from the TCGA database (https://www.cancer.gov/ccg/research/genome-sequencing/tcga). All processed data can be obtained through corresponding authors within reasonable requirements.
