# Supplementary material for: The role of FOXK2–FBXO32 in breast cancer tumorigenesis: Insights into ribosome‐associated pathways
Source: Thorac Cancer. 2024 Nov 18;16(1):e15482. doi: 10.1111/1759-7714.15482 (PMC11729401; doi:10.1111/1759-7714.15482)
Supplement: Supplementary file 1 — Data S1. Supporting Information. [file TCA-16-e15482-s001.docx]

**Supplemental 1.** In total, we included 232 single cell transcriptome samples (normal = 31; adjacent = 54; tumor = 148) from 12 articles for pan-cancer's analysis. Of these, 31 GSM samples were derived from GSE134355; 11 GSM samples were related to GSE141445; 49 samples were derived from the ArrayExpress database at EMBL-EBI (www.ebi.ac.uk/arrayexpress) under accession number E-MTAB-8107; 2 GSM samples were associated with GSE157703; 22 GSM samples were derived from GSE131907; 7 GSM samples were derived from GSE138709; 31 samples were derived from the ArrayExpress database at EMBL-EBI (www.ebi.ac.uk/arrayexpress) under accession number E-MTAB-6149 and E-MTAB-6653; 32 CRR samples were related to GSA database under accession number CRA001160; 10 GSM samples were related to GSE154778; 7 HRR samples were available in GSA-Human under the accession code HRA000212; 10 thyroid samples were derived from GSA-Human database under accession number HRA000686; 20 gastric samples were download from <http://dna-discovery.stanford.edu/download/1401/>

**Supplemental 2.** The immune phenotype score (IPS) was used to calculate the scores of four different immune phenotypes (antigen presentation, effector cells, suppressor cells, checkpoint). The IPS z-score is the integration of the four, and the higher the IPS z-score, the stronger the sample immunogenicity.

**Supplemental3.**
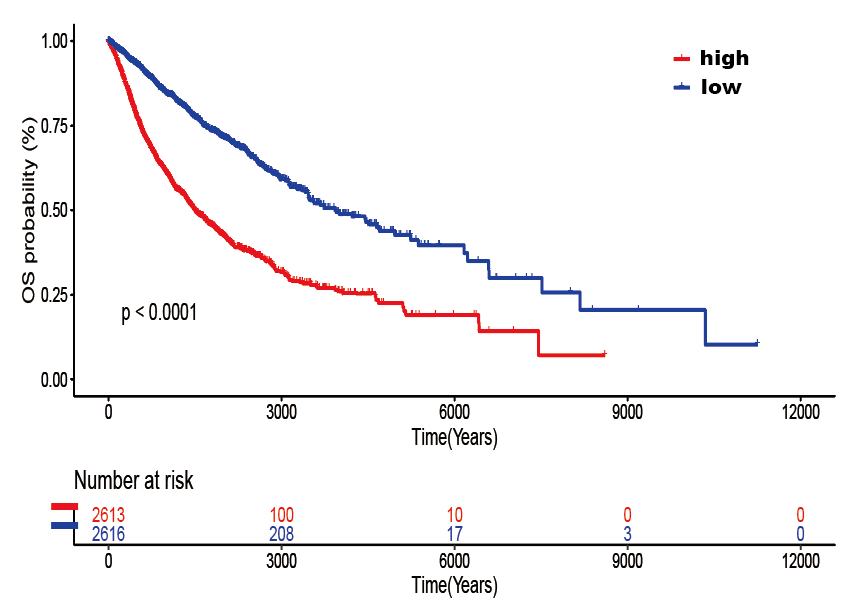


High and low expression of pan-cancer FOXK2 OS in GTEx database


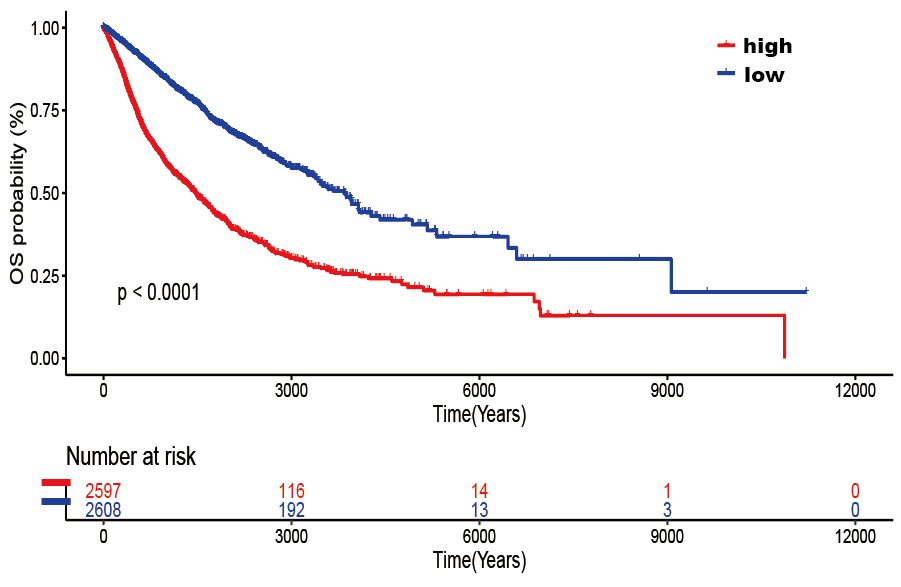


High and low expression of pan-cancer FOXK2 OS in IGCG database


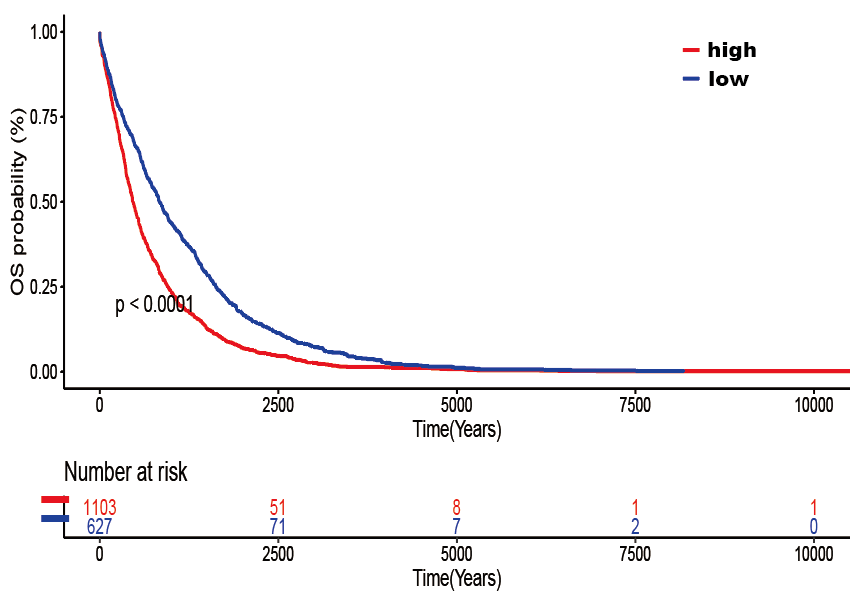


High and low expression of pan-cancer FOXK2 OS in CGGA database

**Supplemental 4.** GEPIA2 is a web server for analyzing the RNA sequencing expression data of 9,736 tumors and 8,587 normal samples from the TCGA and the GTEx projects, using a standard processing pipeline. The data matrix information has been converted to log2 (TPM+0.001).
