# Supplementary figures and images for: The role of FOXK2–FBXO32 in breast cancer tumorigenesis: Insights into ribosome‐associated pathways
Source: Thorac Cancer. 2024 Nov 18;16(1):e15482. doi: 10.1111/1759-7714.15482 (PMC11729401; doi:10.1111/1759-7714.15482)

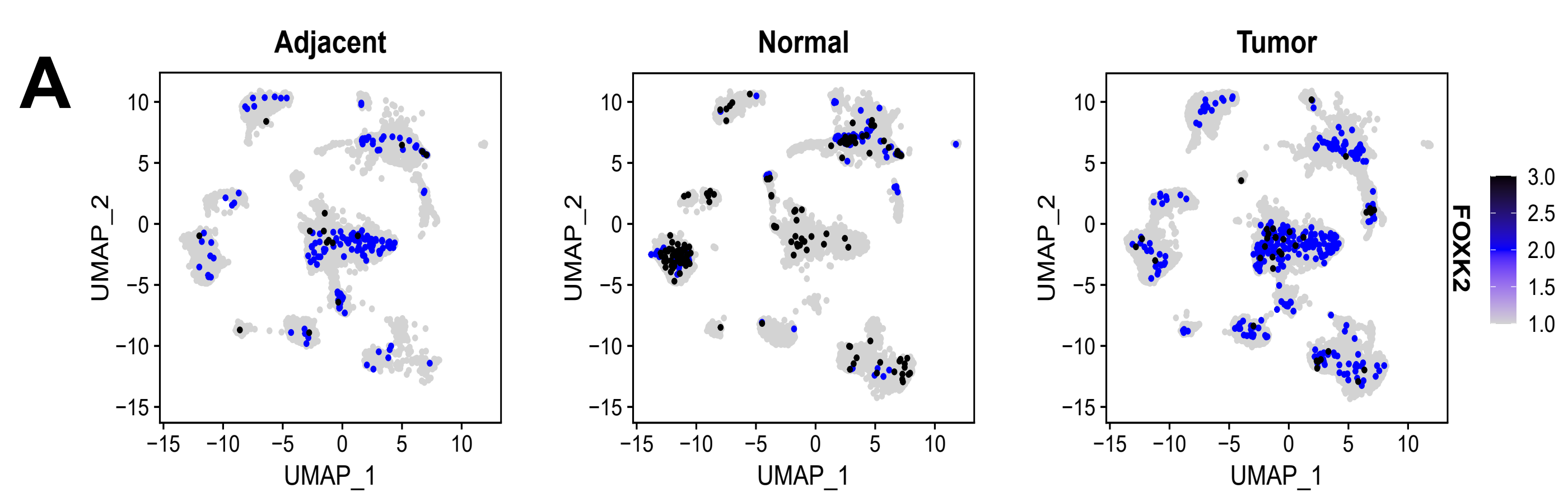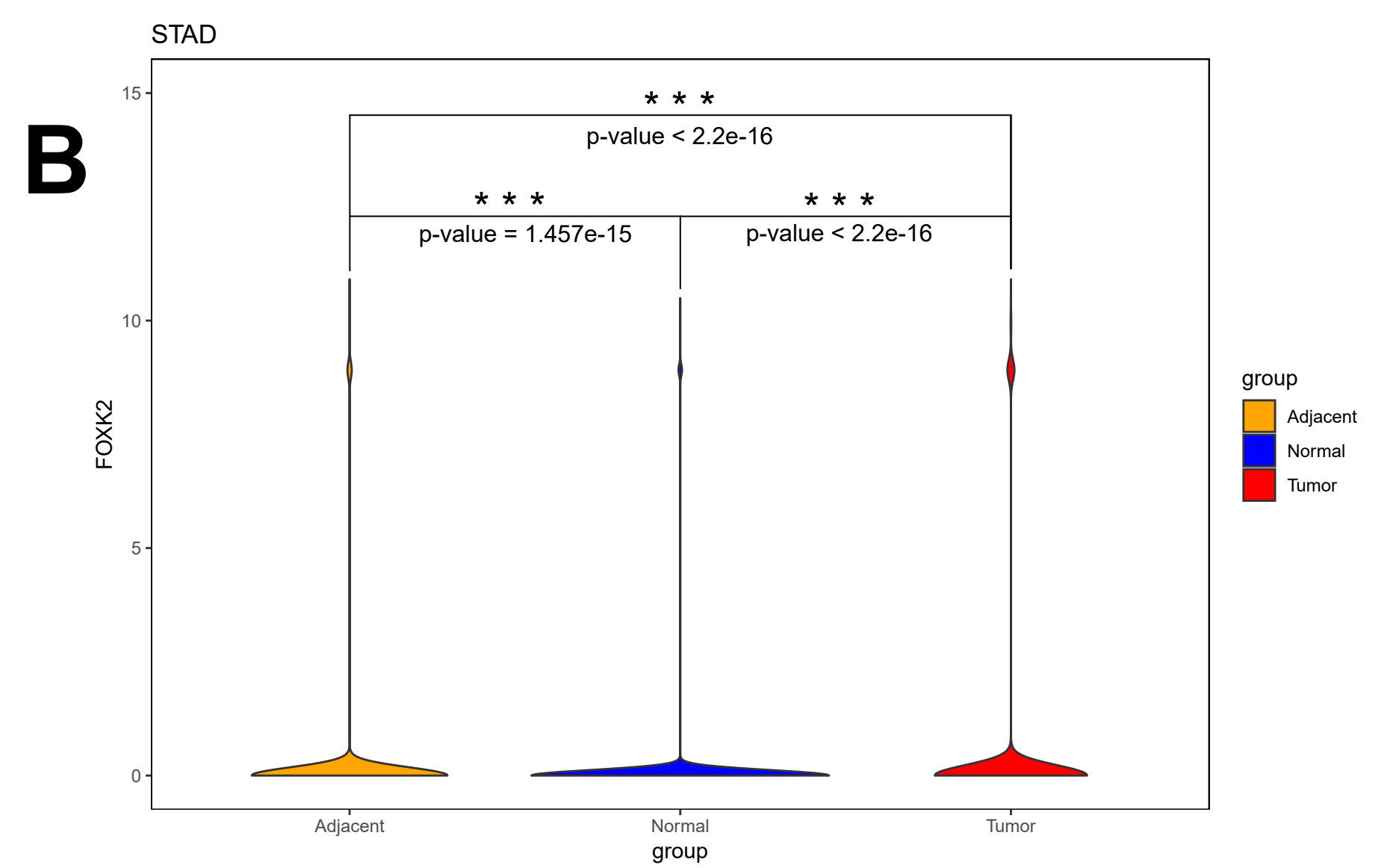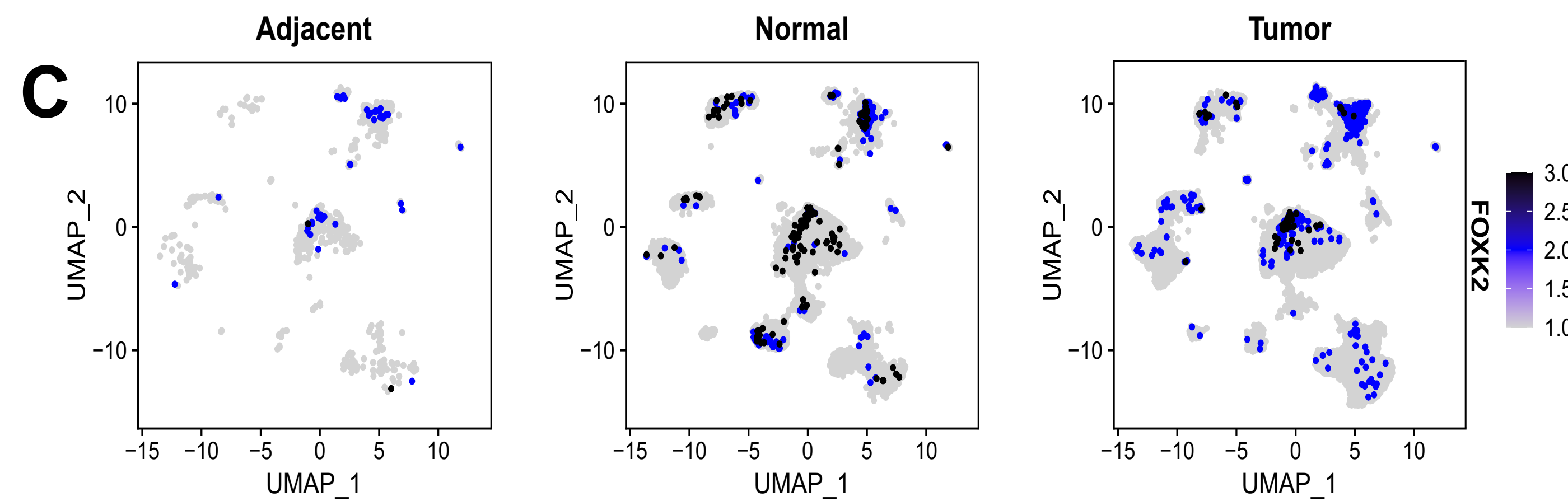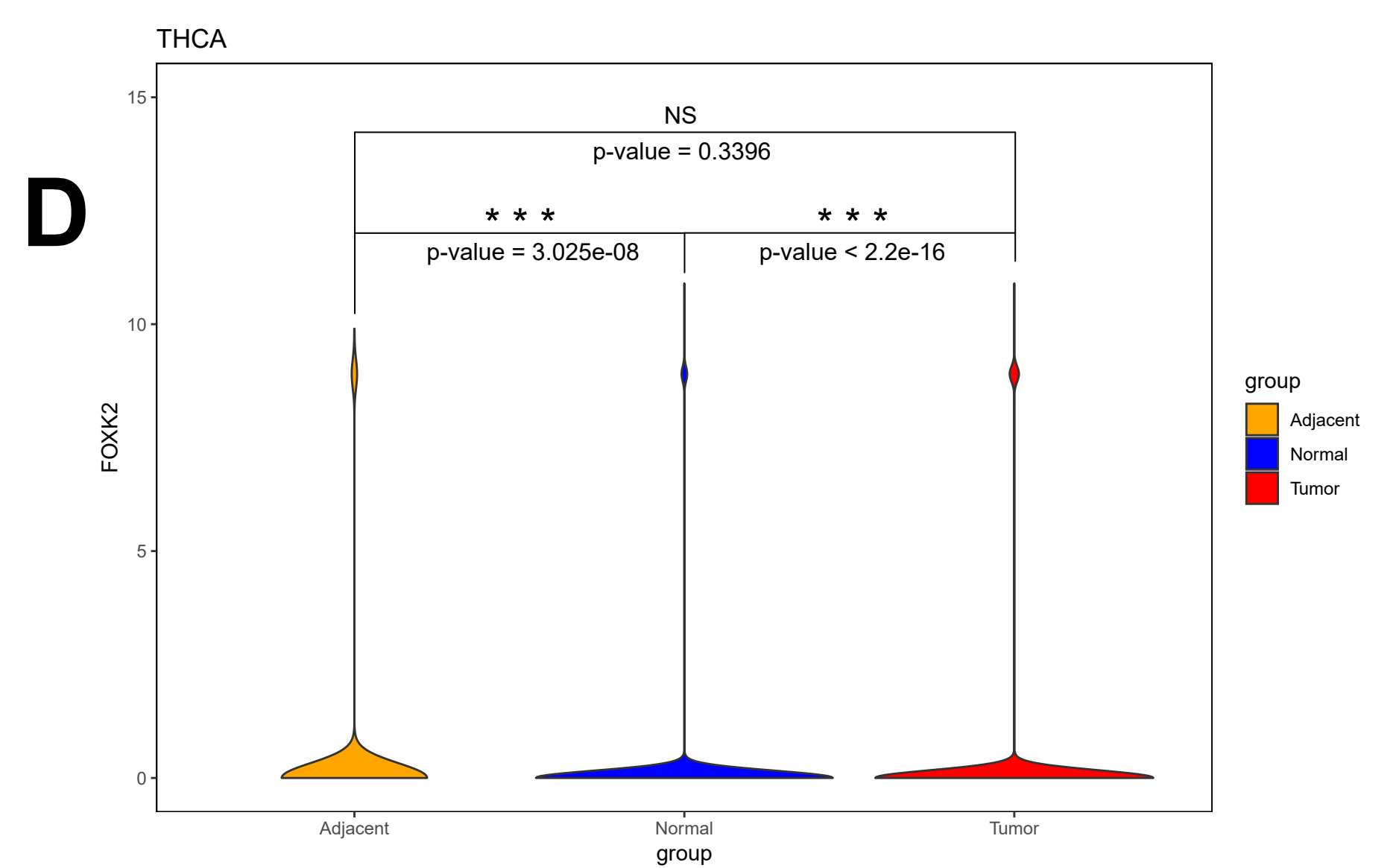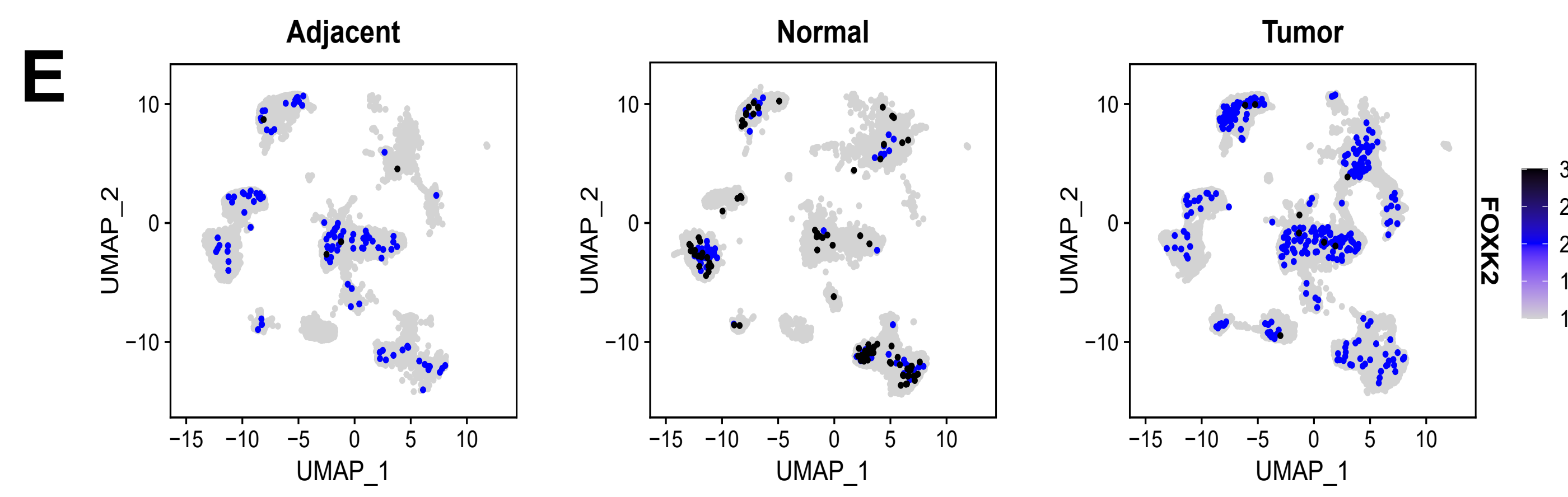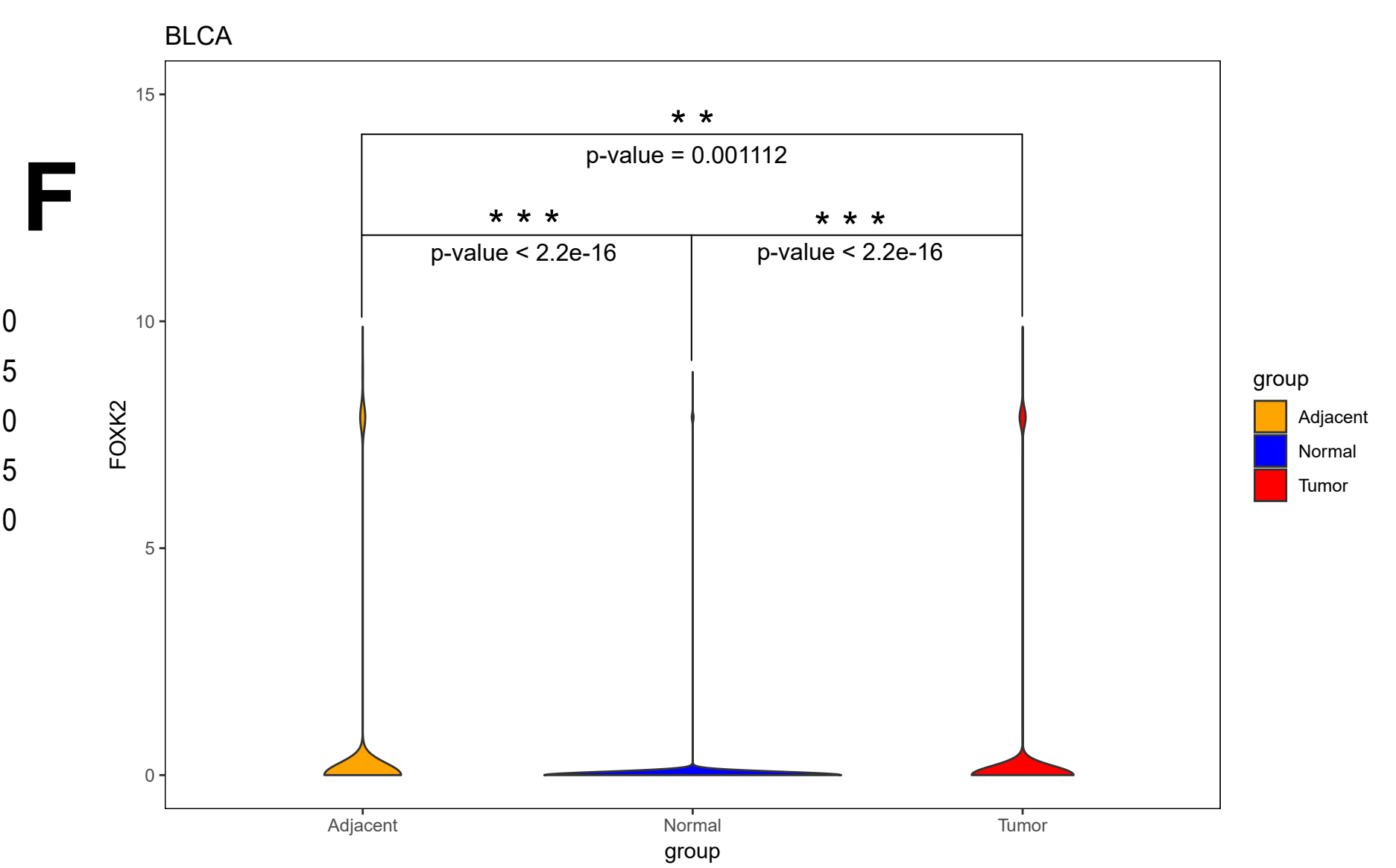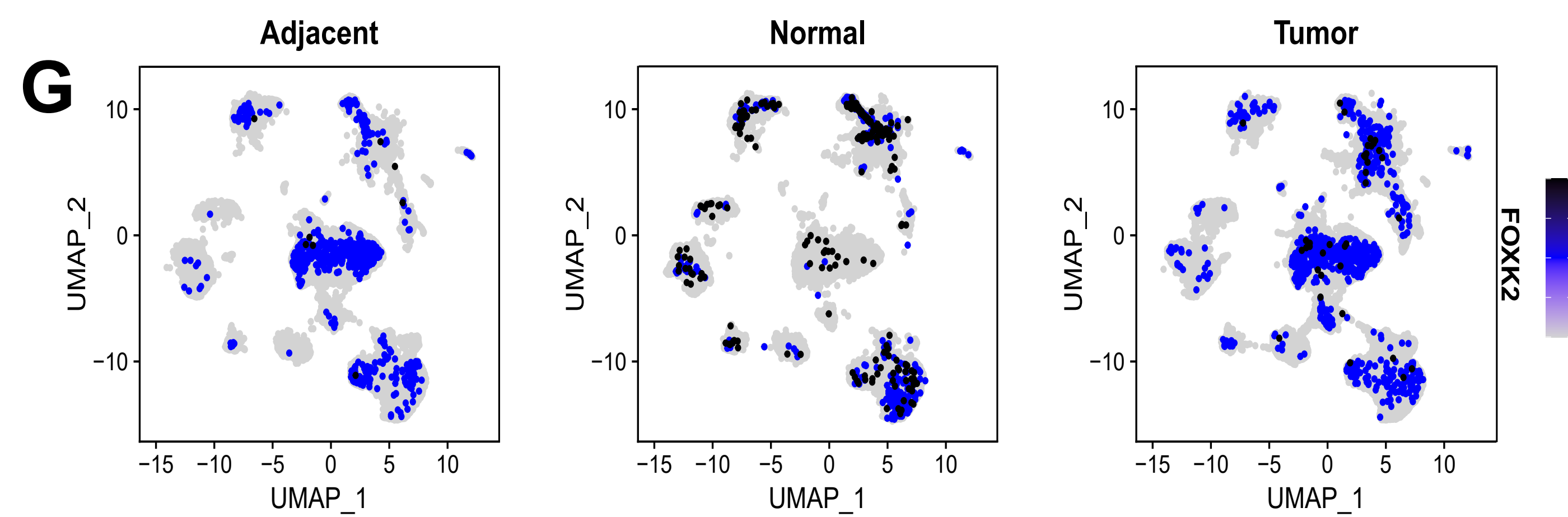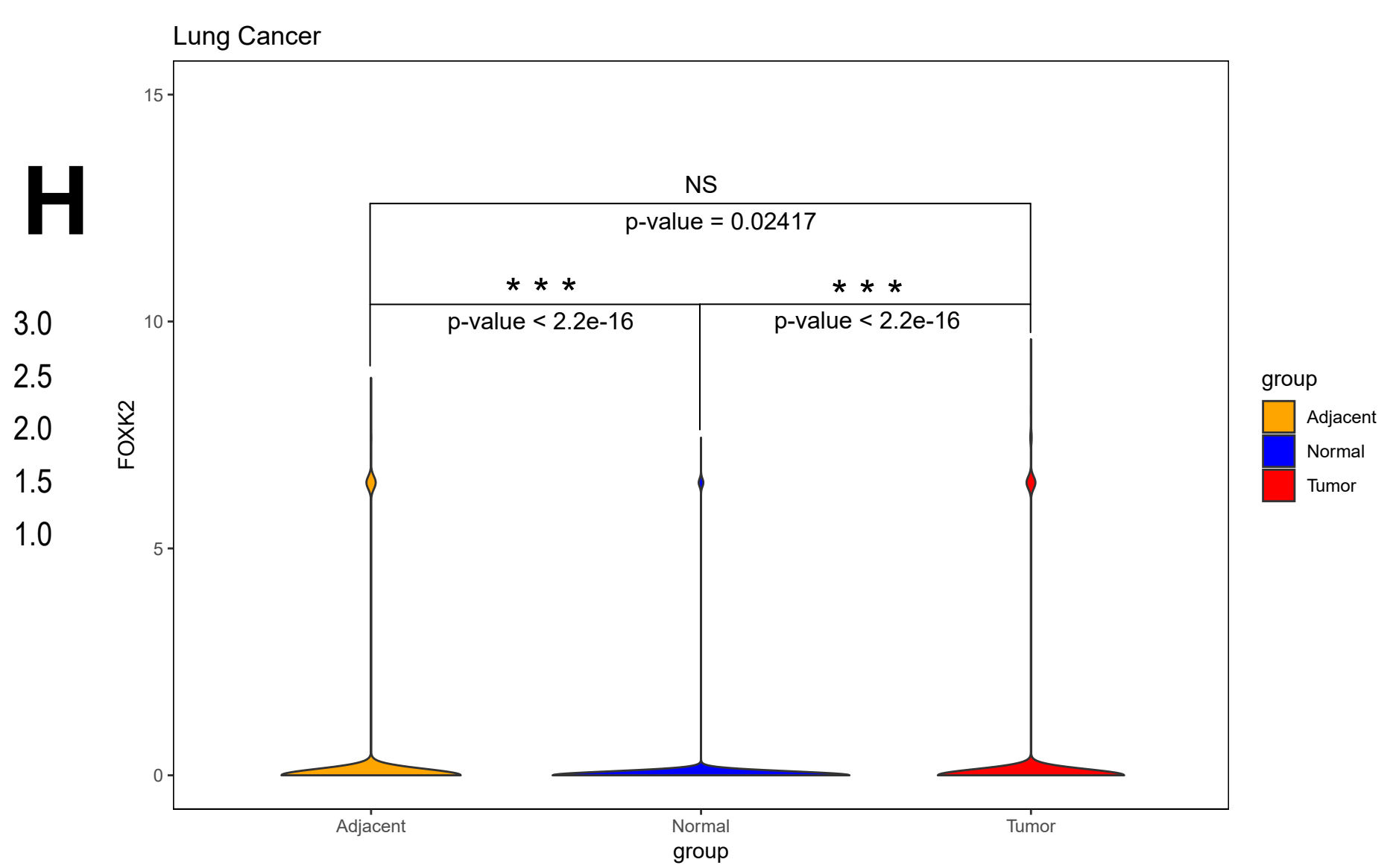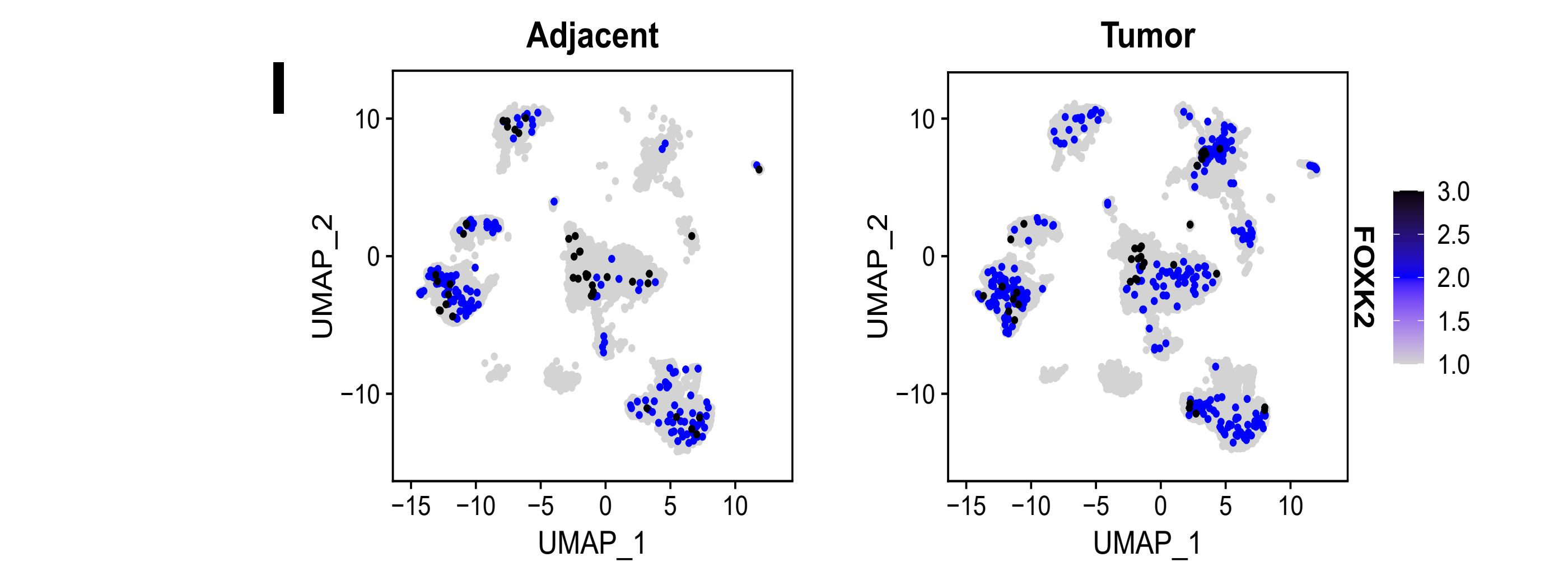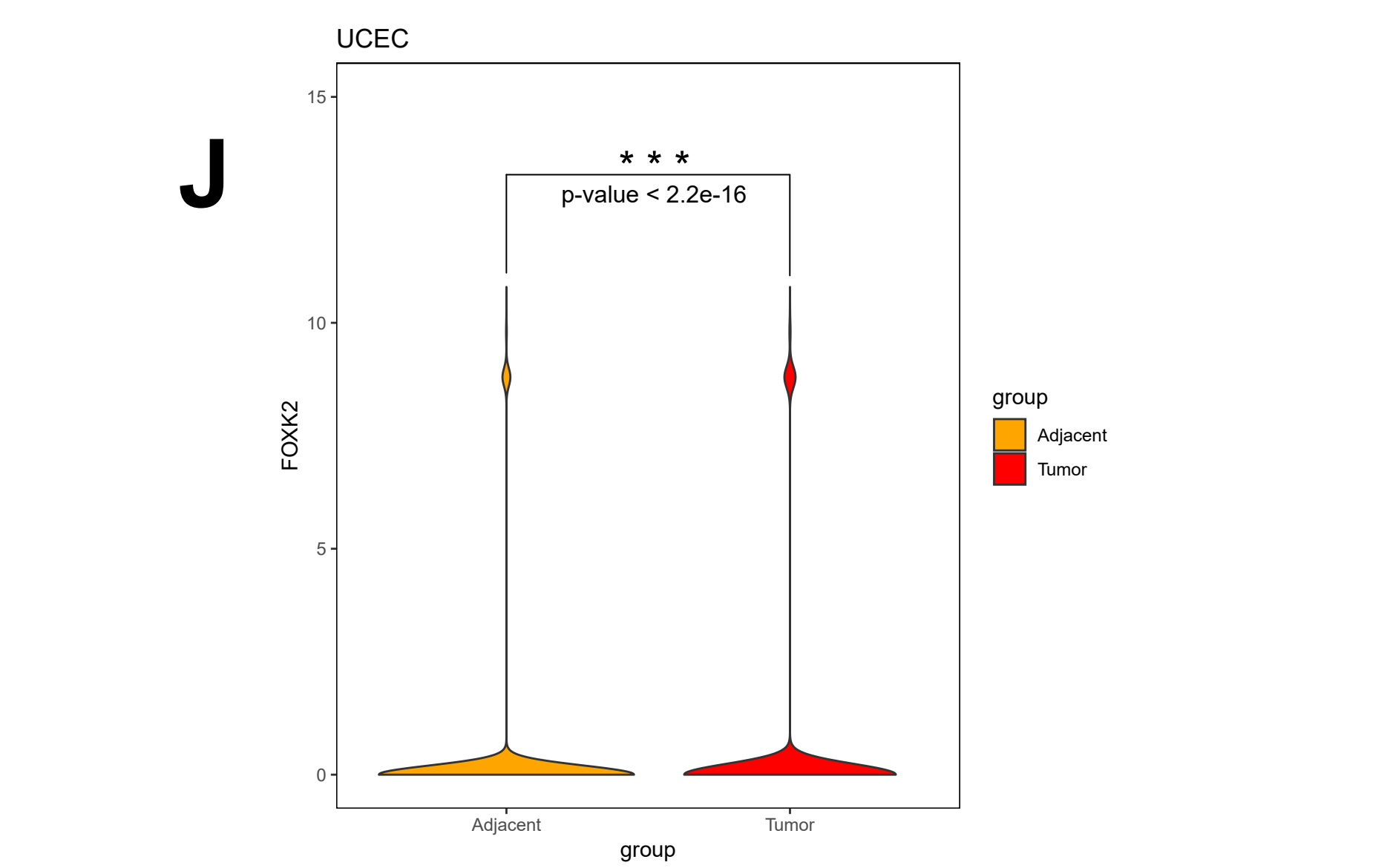

Supplement: Supplementary file 2 — Figure S1. Single‐cell expression of FOXK2 in other types of cancer. (a) Distribution in gastric cancer; (b) differential analysis of expression in gastric cancer; (c) distribution in thyroid cancer; (d) differential analysis of expression in thyroid cancer; (e) the distribution in bladder cancer; (f) differential analysis of expression in bladder cancer; (g) distribution in lung cancer; (h) differential analysis of expression in lung cancer; (i) distribution in endometrial cancer; (j) differential analysis of expression in endometrial cancer. [file TCA-16-e15482-s008.pdf]

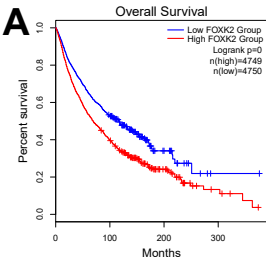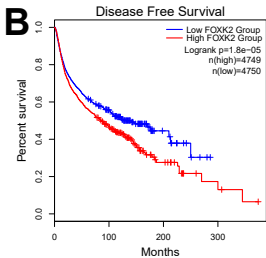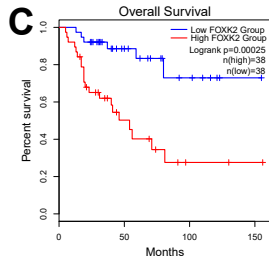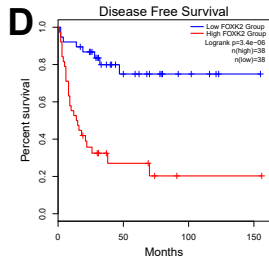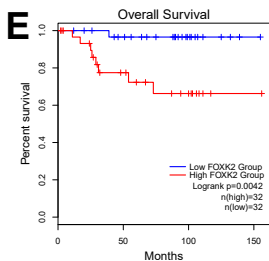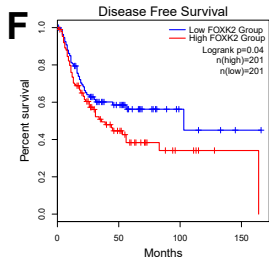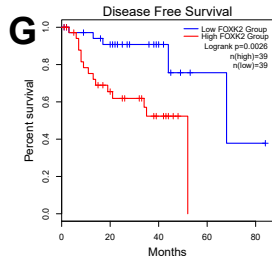

Supplement: Supplementary file 3 — Figure S2. OS (overall survival) and DFS (disease‐free survival) analysis of FOXK2. (A) OS of FOXK2 in pan‐cancer (33 types of cancer in the TCGA database); (B) DFS of FOXK2 in pan‐cancer; (C) OS of FOXK2 in ACC; (D) DFS of FOXK2 in ACC; (E) OS of FOXK2 in KICH; (F) DFS of FOXK2 in BLCA; (G) DFS of FOXK2 in UVM. [file TCA-16-e15482-s003.pdf]

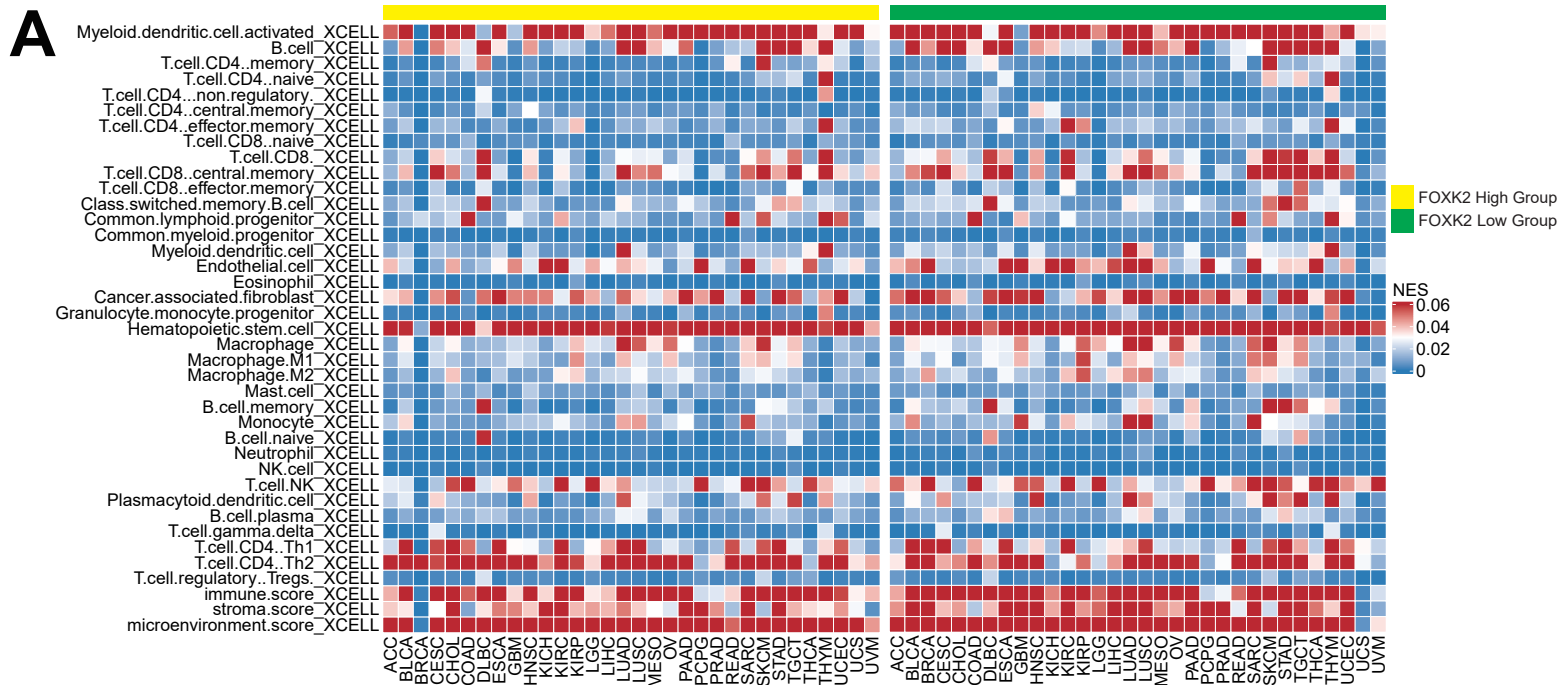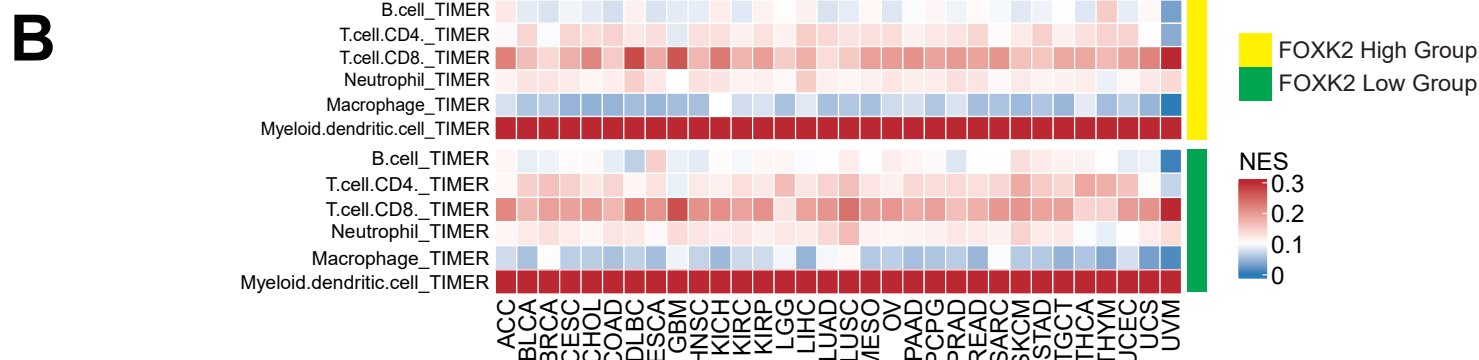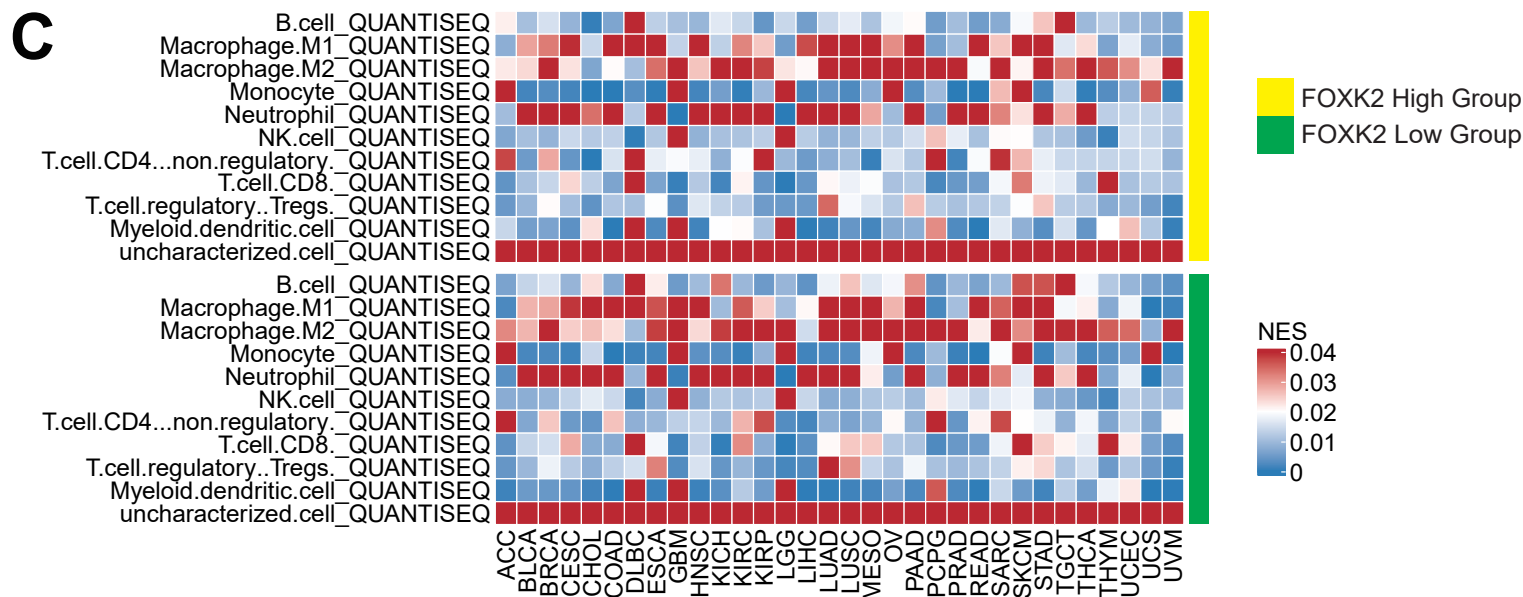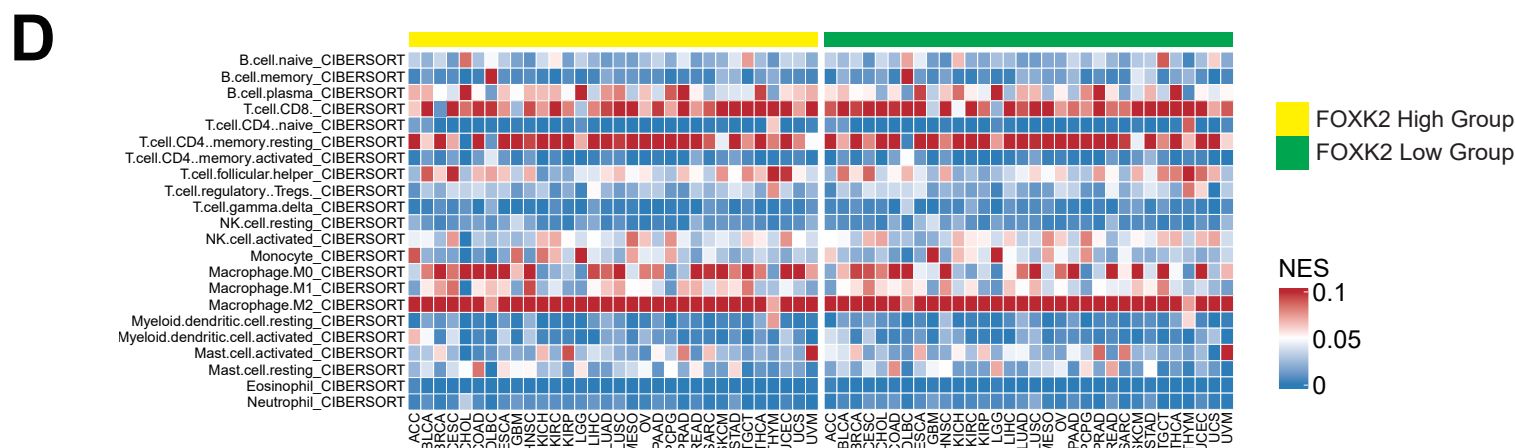

Supplement: Supplementary file 4 — Figure S3. Correlation between FOXK2 expression and immune cell infiltration. (A) Correlation between FOXK2 expression and ESTIMATE Score; (B) the correlation between FOXK2 expression and Stromal Score; (C) the correlation between FOXK2 expression and Immune Score; (D) heat maps of FOXK2 expression and immune infiltration levels were performed using quanTIseq, TIMER, and xCell algorithms, respectively. [file TCA-16-e15482-s002.pdf]

A

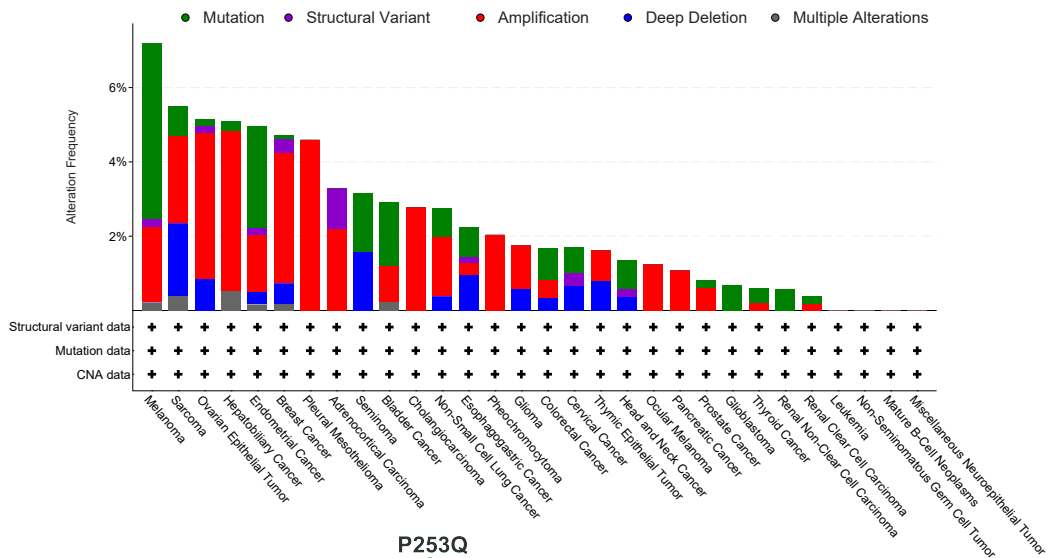

B

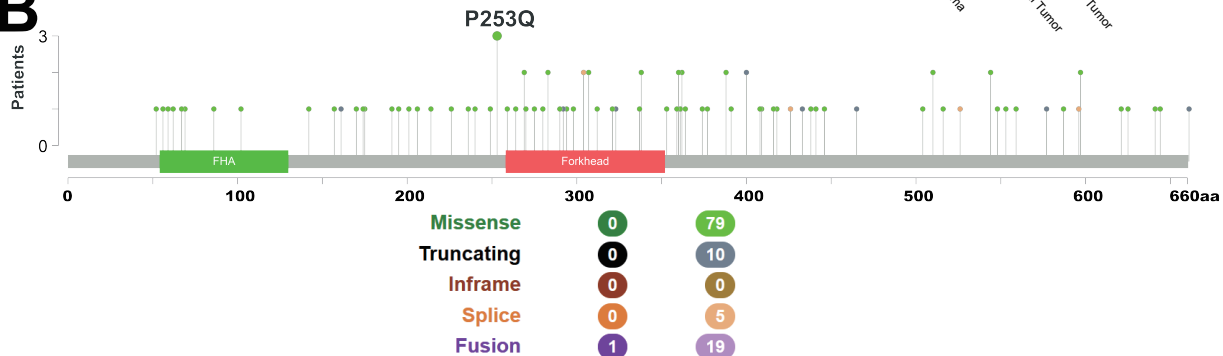

C

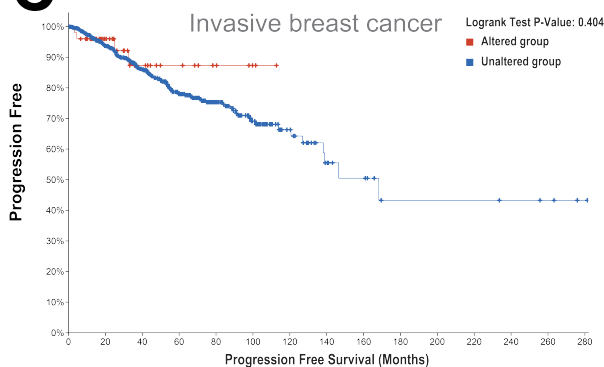

D

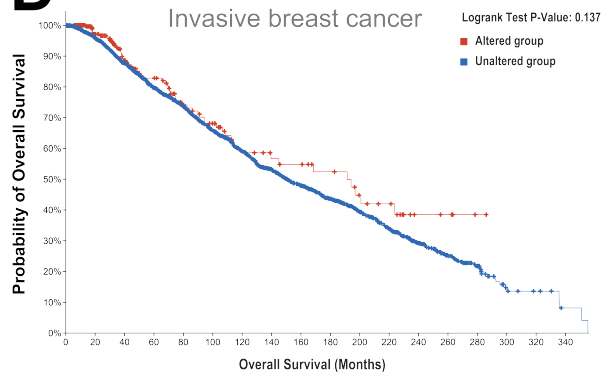

Supplement: Supplementary file 5 — Figure S4. Mutations of FOXK2 in different tumor tissues. (A) Frequency and range of FOXK2 mutations; (B) mutation site of FOXK2; (C) the location of the most frequent mutation site (X304 splice) on the 3D structure; (D) Kaplan–Meier curves of OS and PFS in UCEC patients with and without FOXK2 mutations. [file TCA-16-e15482-s005.pdf]

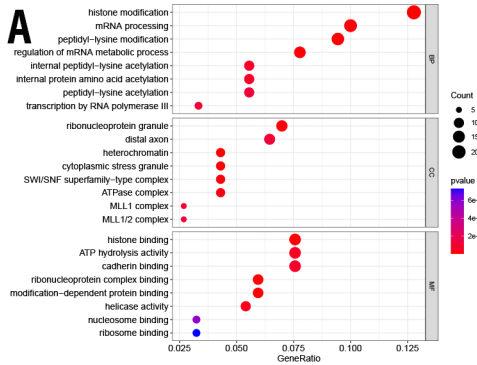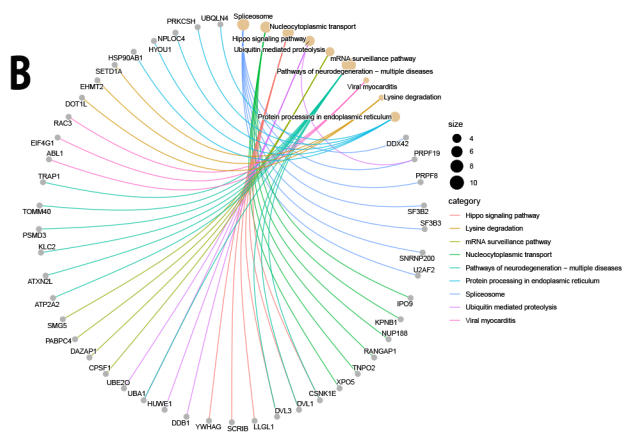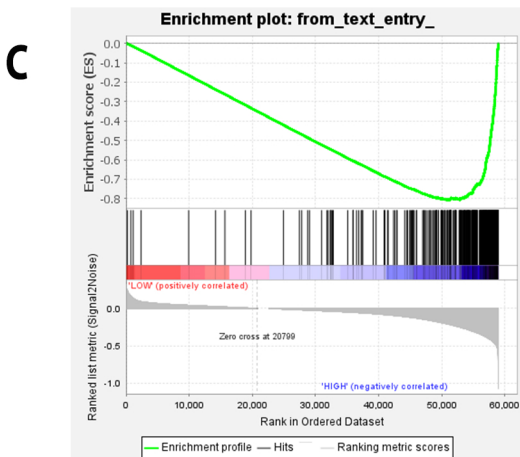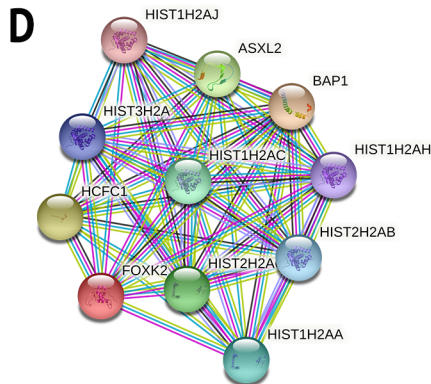

Supplement: Supplementary file 6 — Figure S5. Enrichment analysis of FOXK2‐related genes and proteins. (A) The top 10 GO enrichment significance terms of FOXK2‐related genes in the three functional groups of biological process (BP), cell composition (CC), and molecular function (MF); (B) KEGG pathway analysis of FOXK2‐related genes; (C) GSEA enrichment analysis of FOXK2‐related genes; (D) Foxk2 PPI network created using string tools. Each node represents all proteins generated by a single protein coding gene locus, and each edge represents the predicted functional association. [file TCA-16-e15482-s004.pdf]

**A**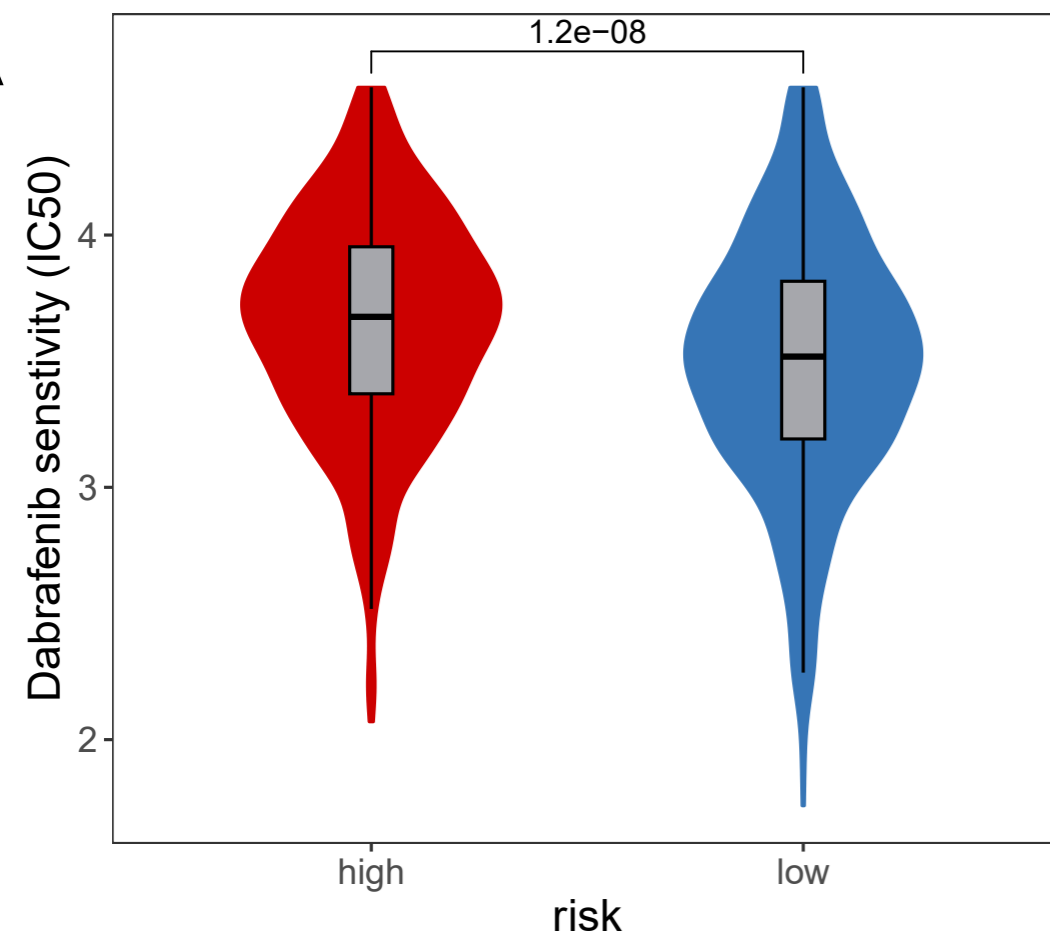**B**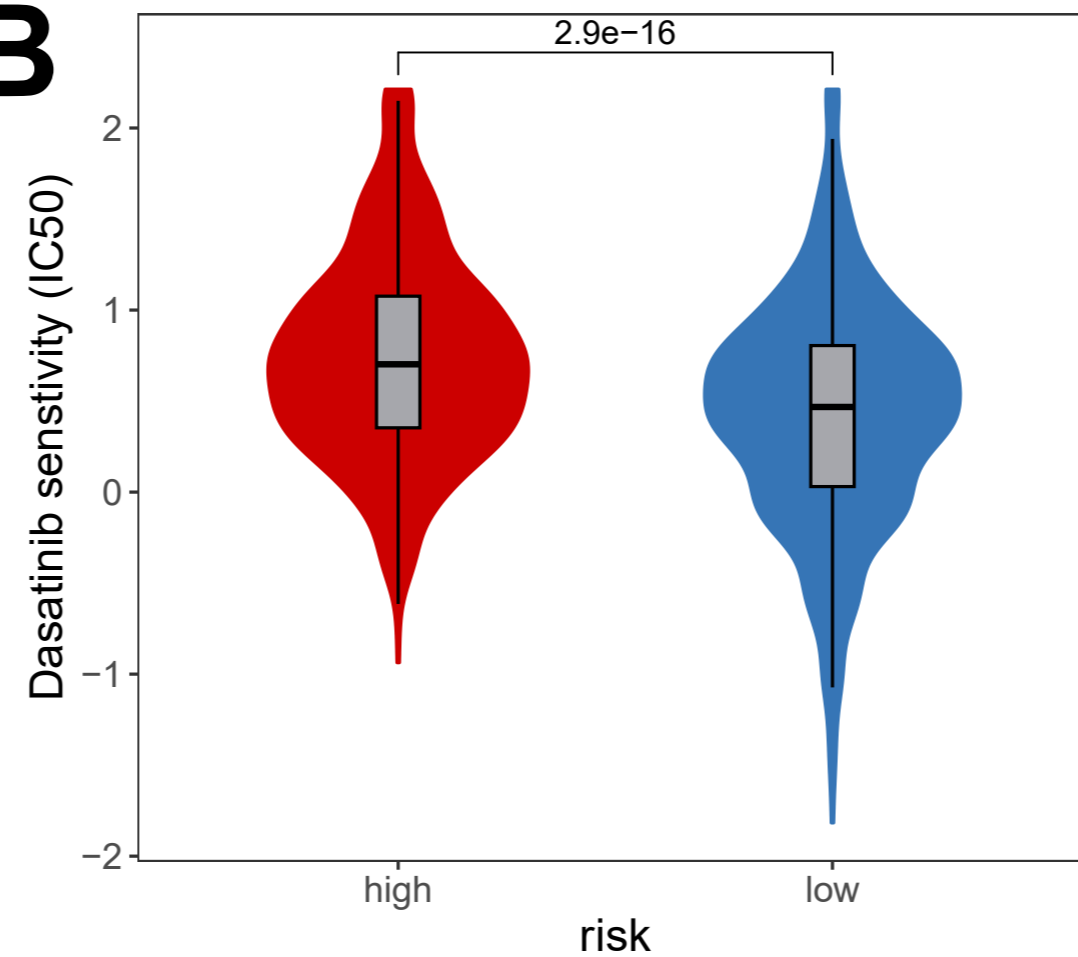**C**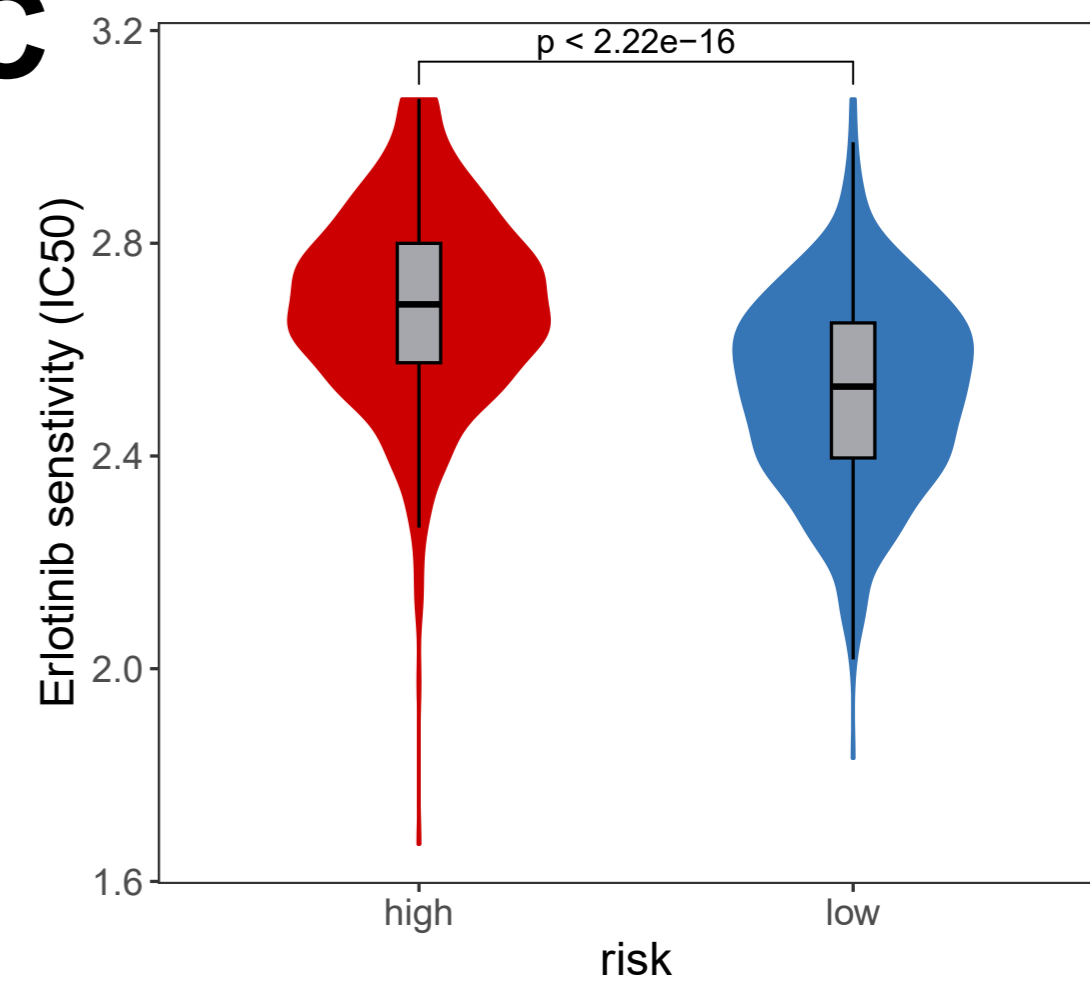**D**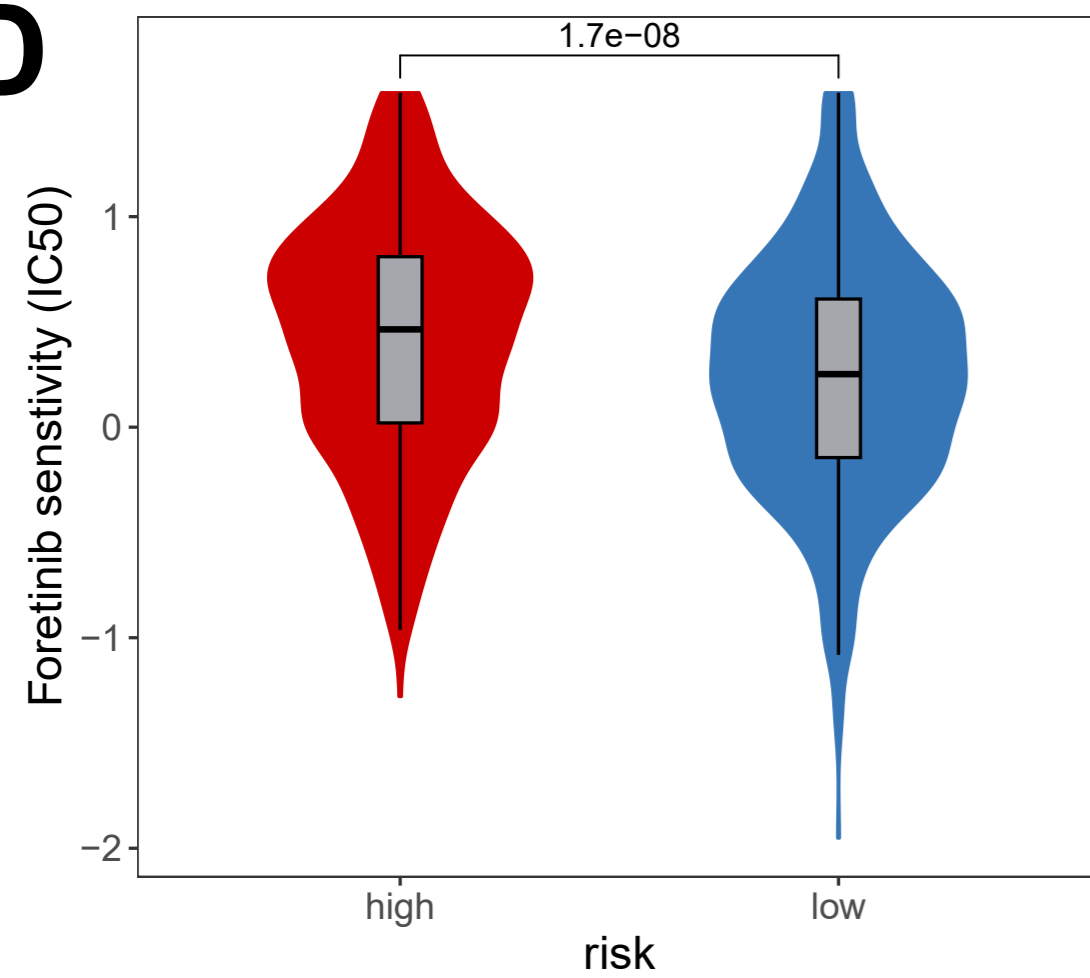**E**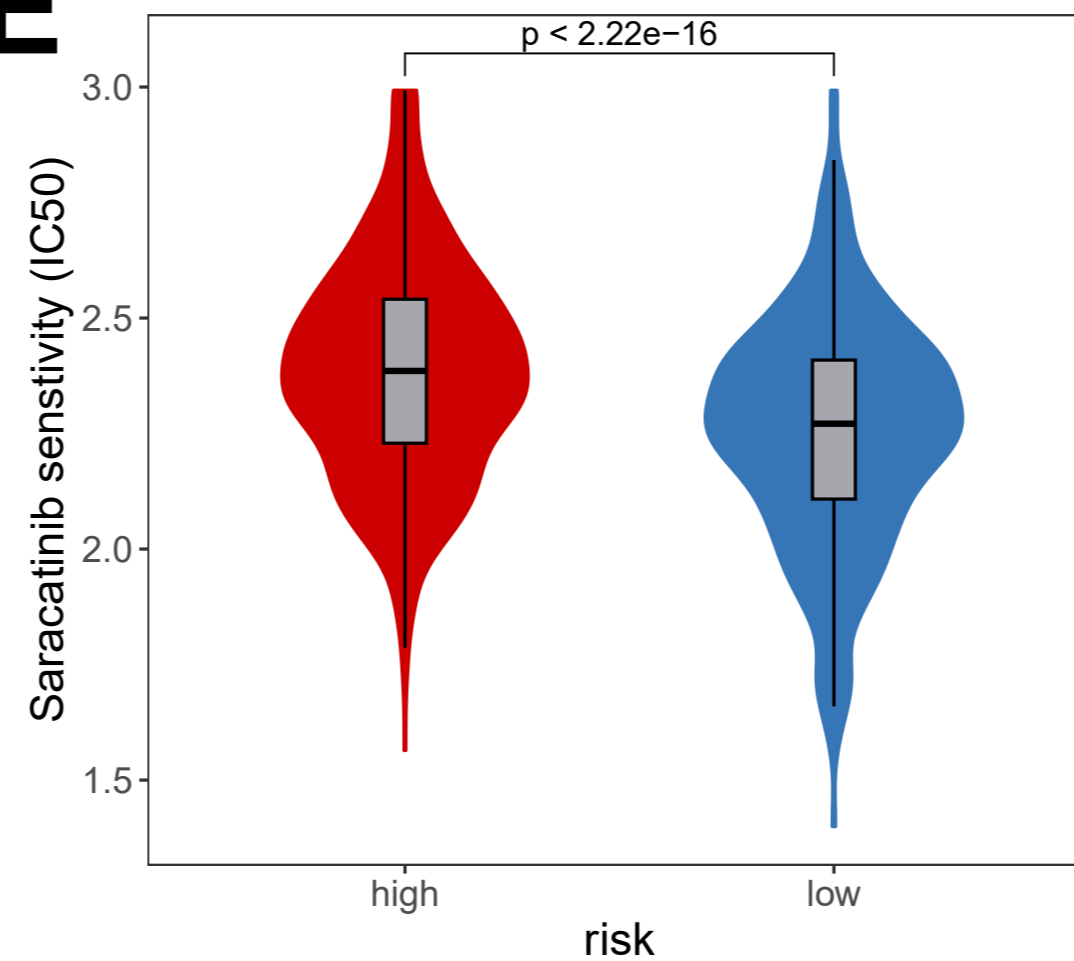**F**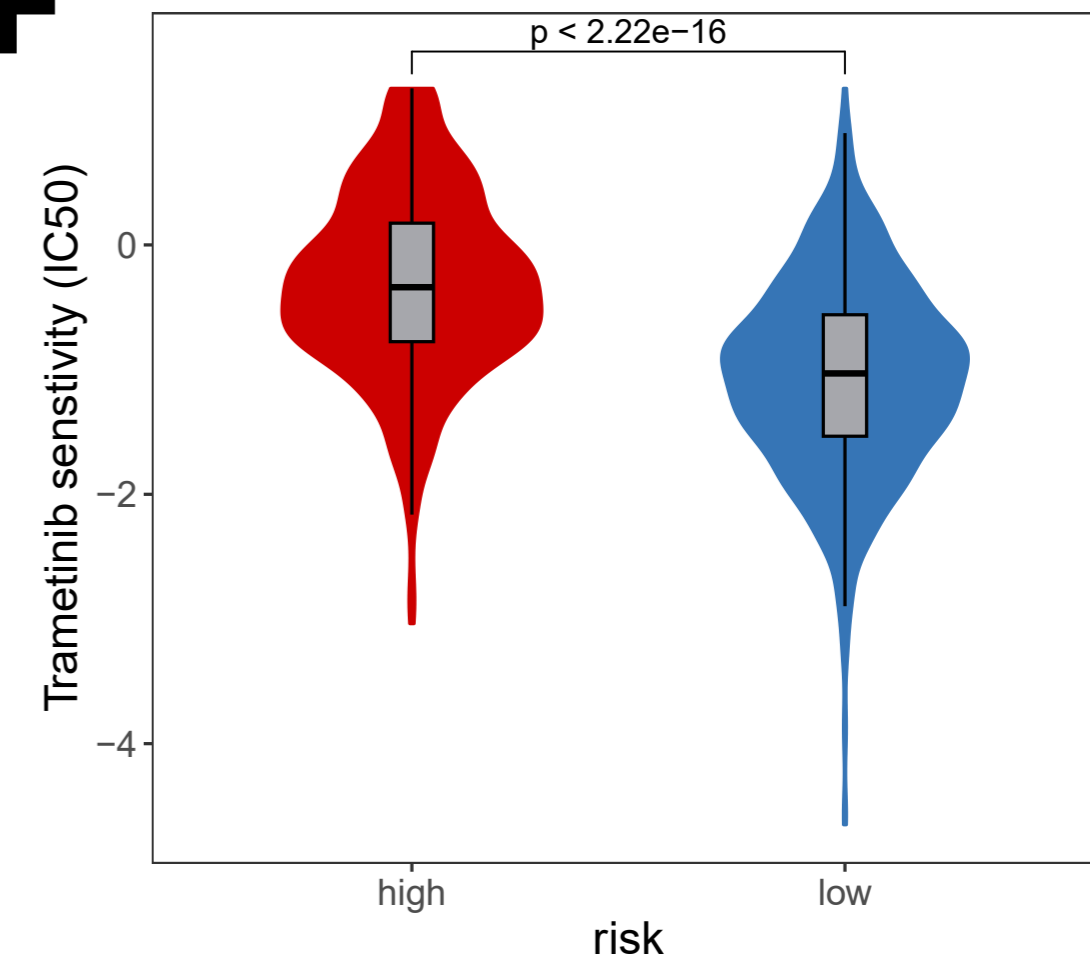

Supplement: Supplementary file 8 — Figure S7. Validation of FOXK2 expression, prediction of immunotherapy, and drug sensitivity testing. (A) Sensitivity testing of FOXK2 in dabrafenib; (B) sensitivity testing of FOXK2 in dasatinib; (C) sensitivity testing of FOXK2 in erlotinib; (D) sensitivity testing of FOXK2 in foretinib; (E) sensitivity testing of FOXK2 in saracatinib; (F) sensitivity testing of FOXK2 in trametinib. [file TCA-16-e15482-s007.pdf]

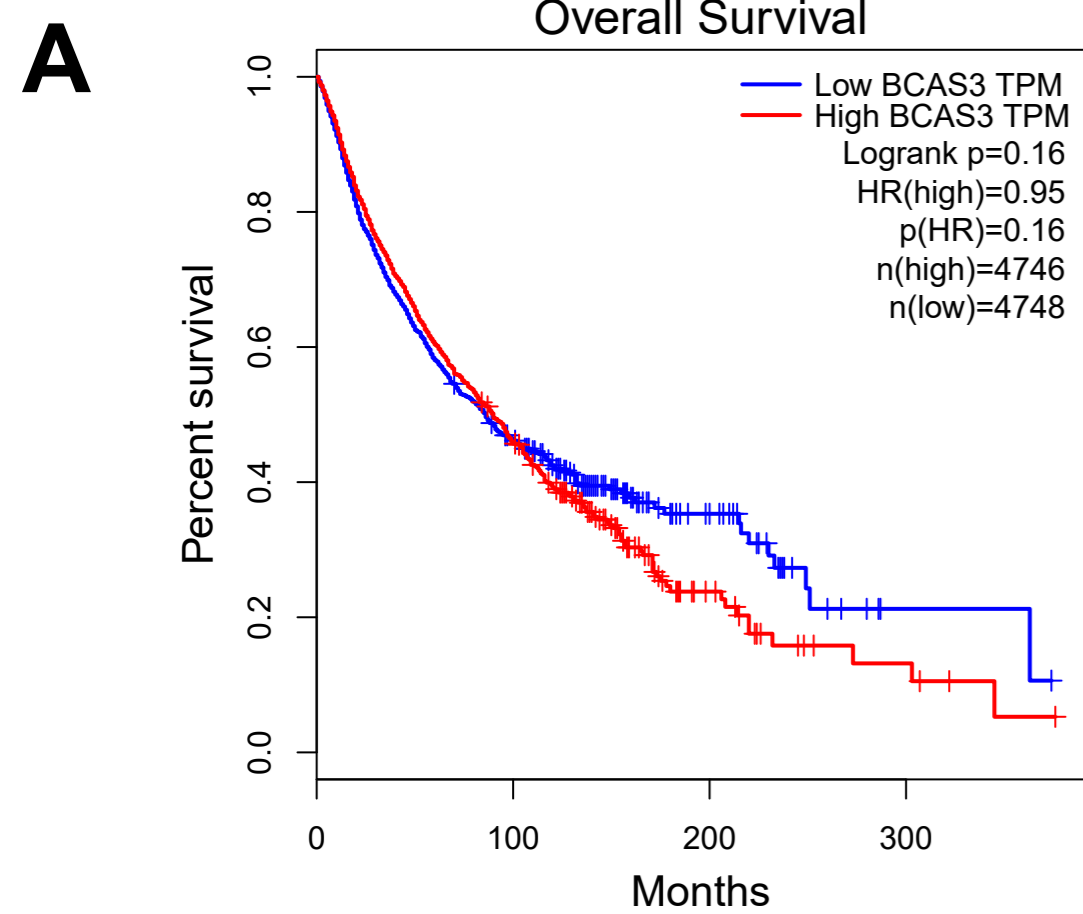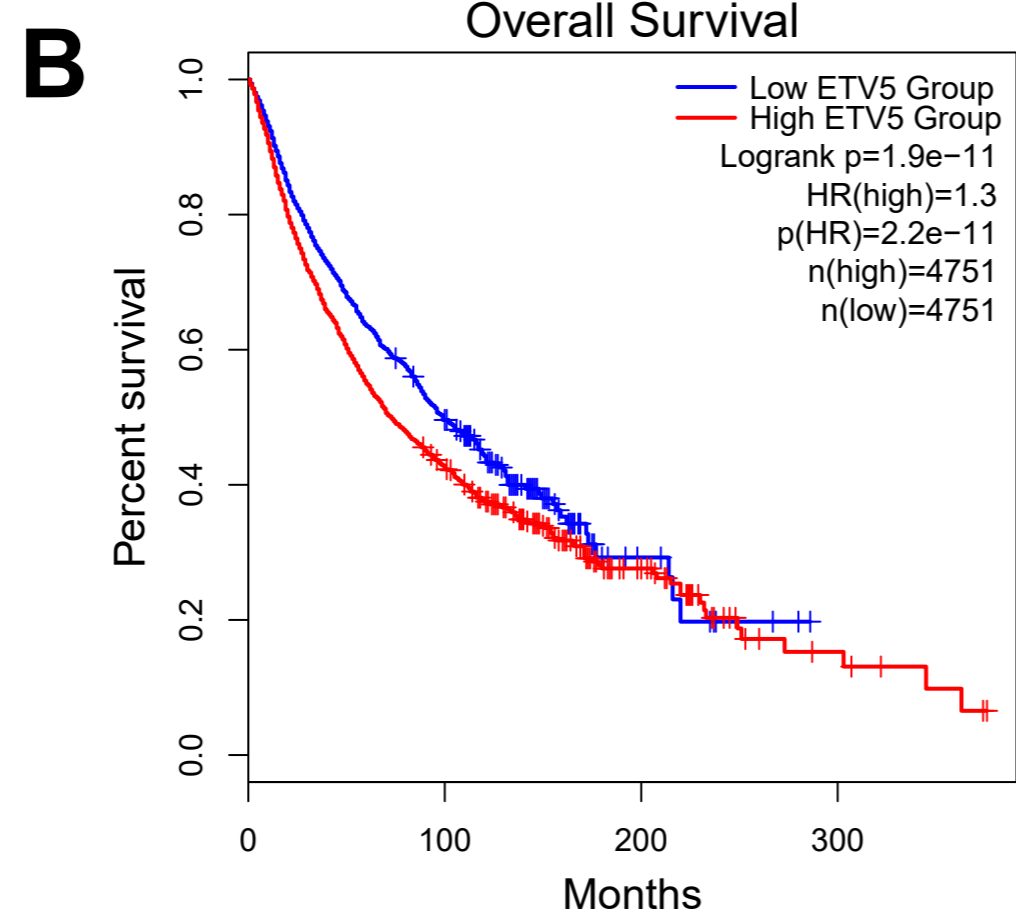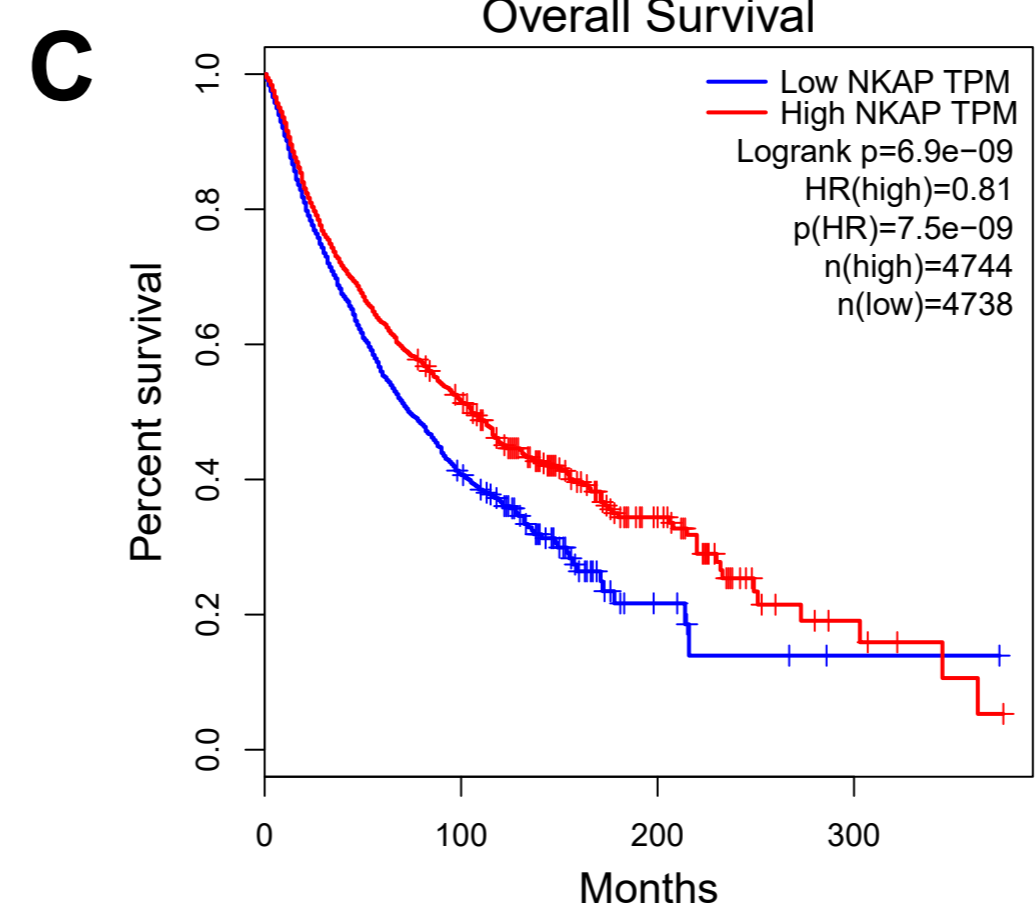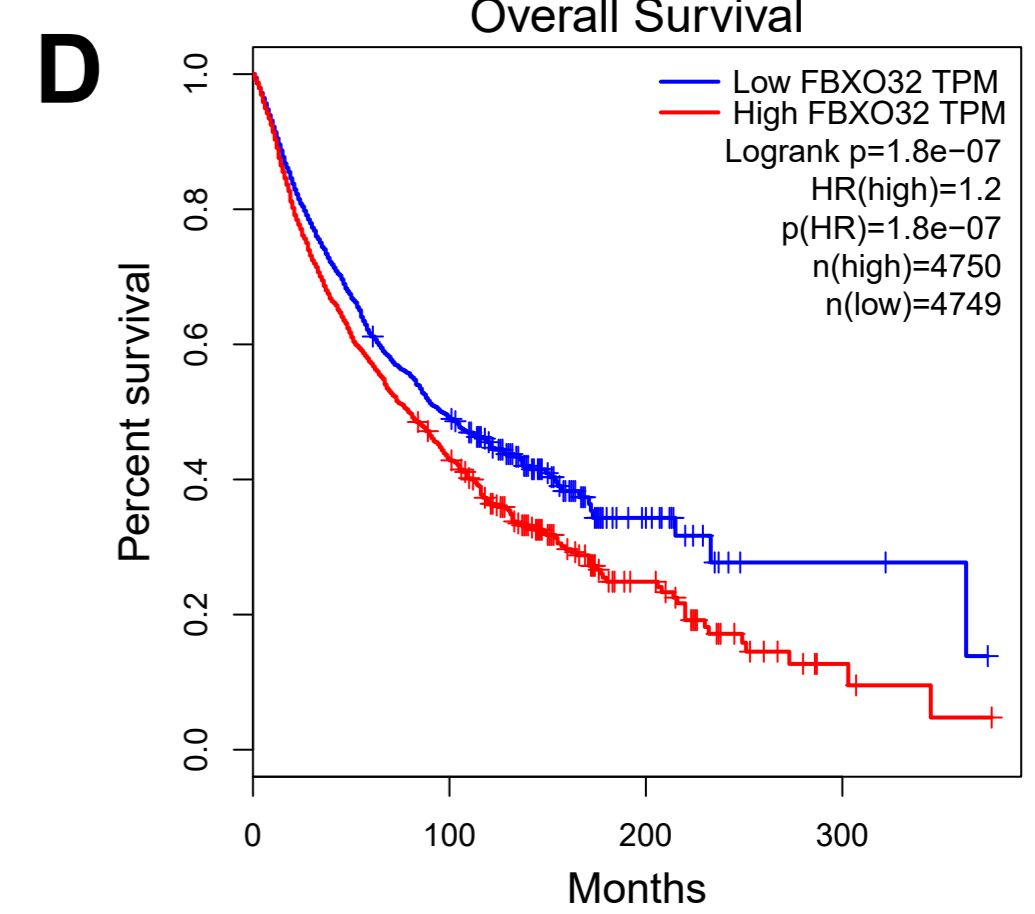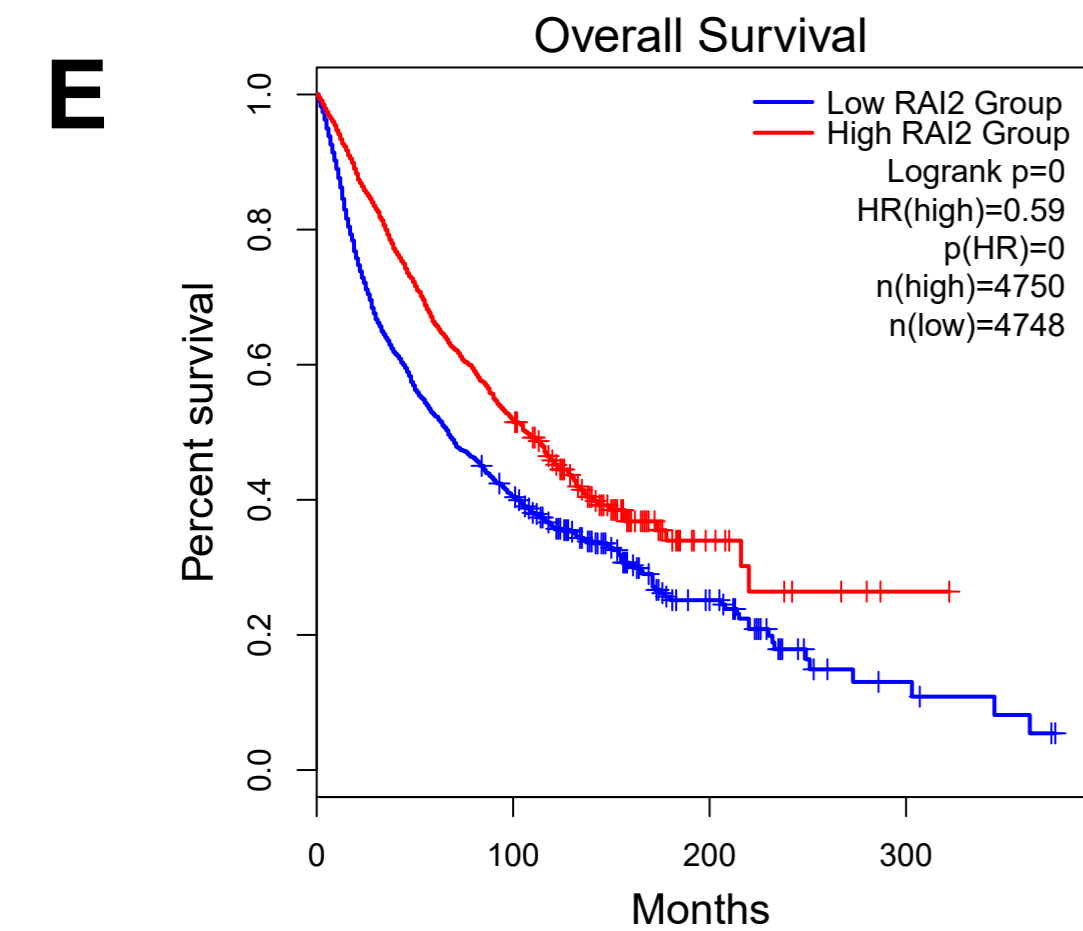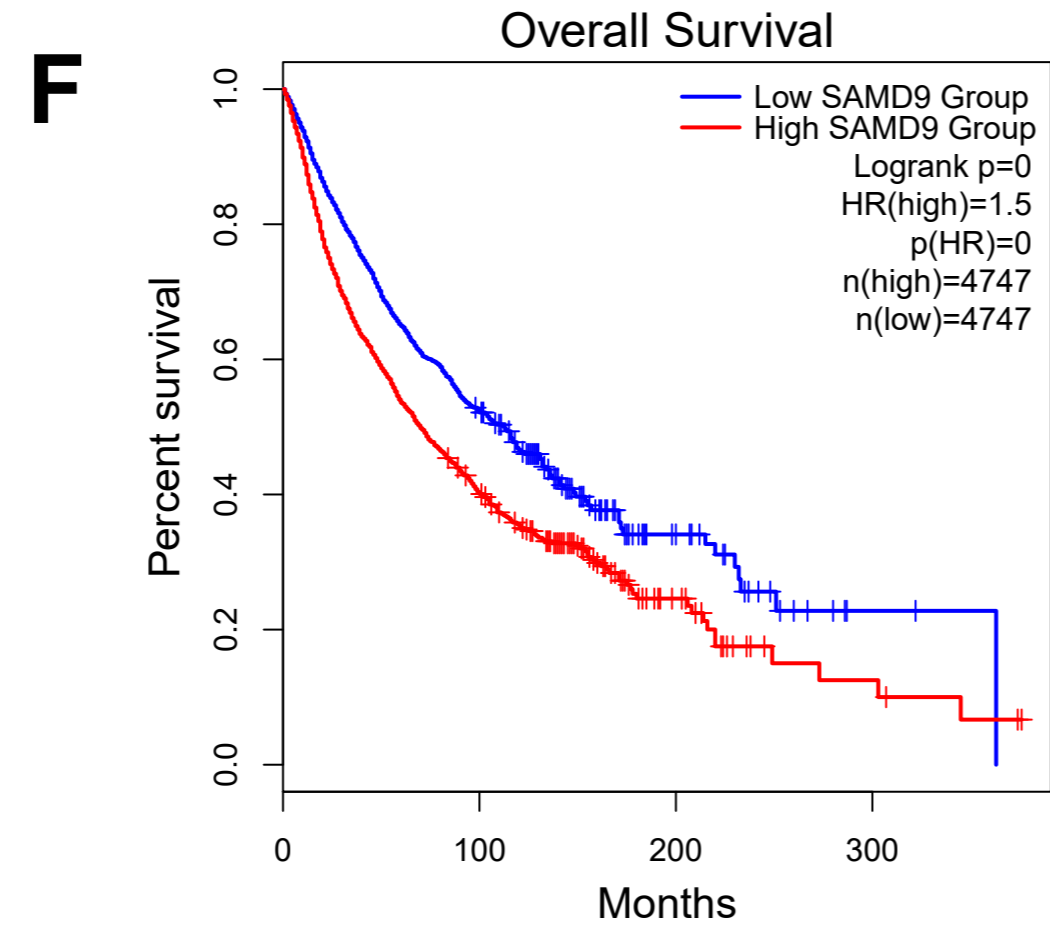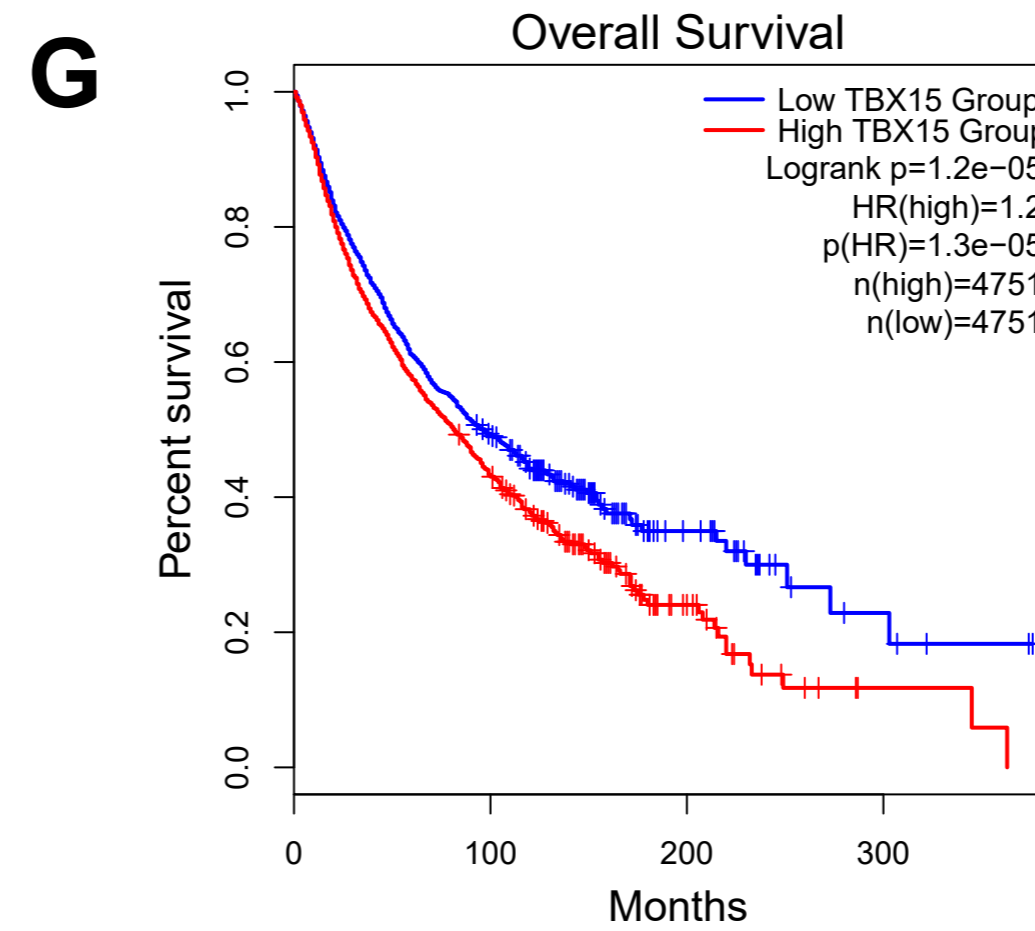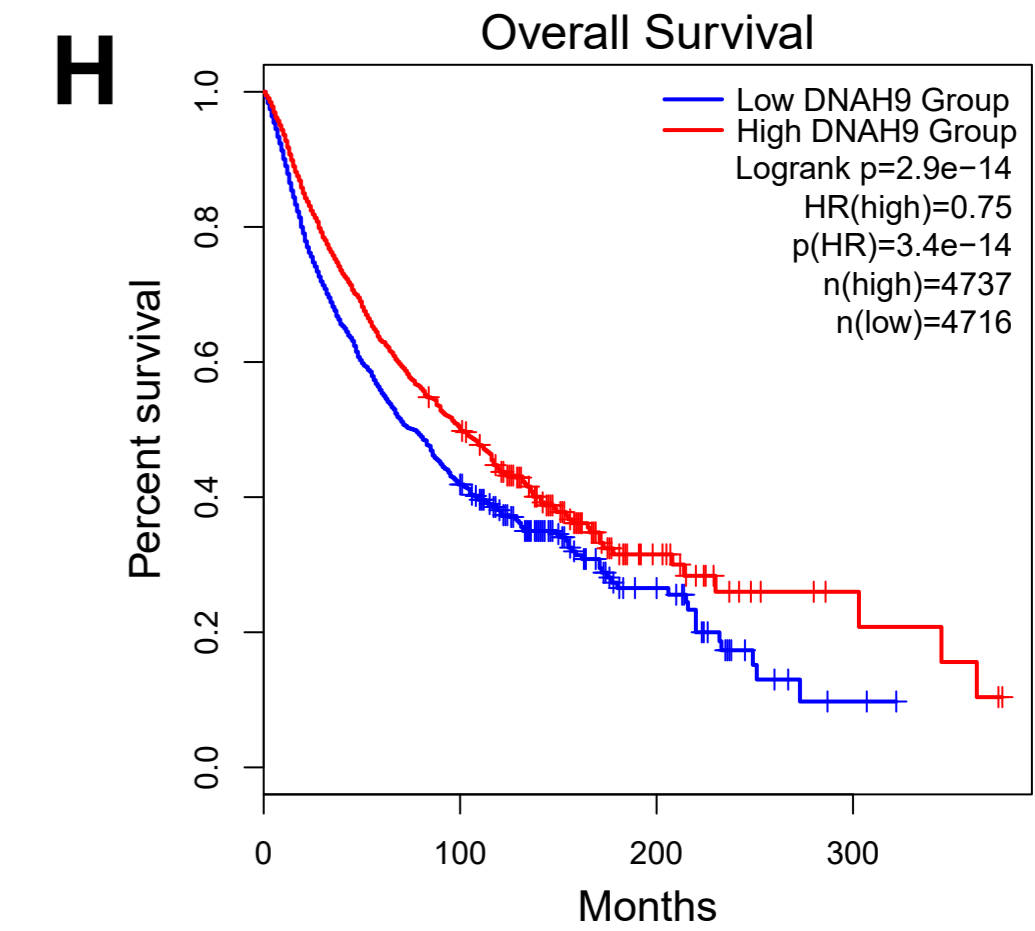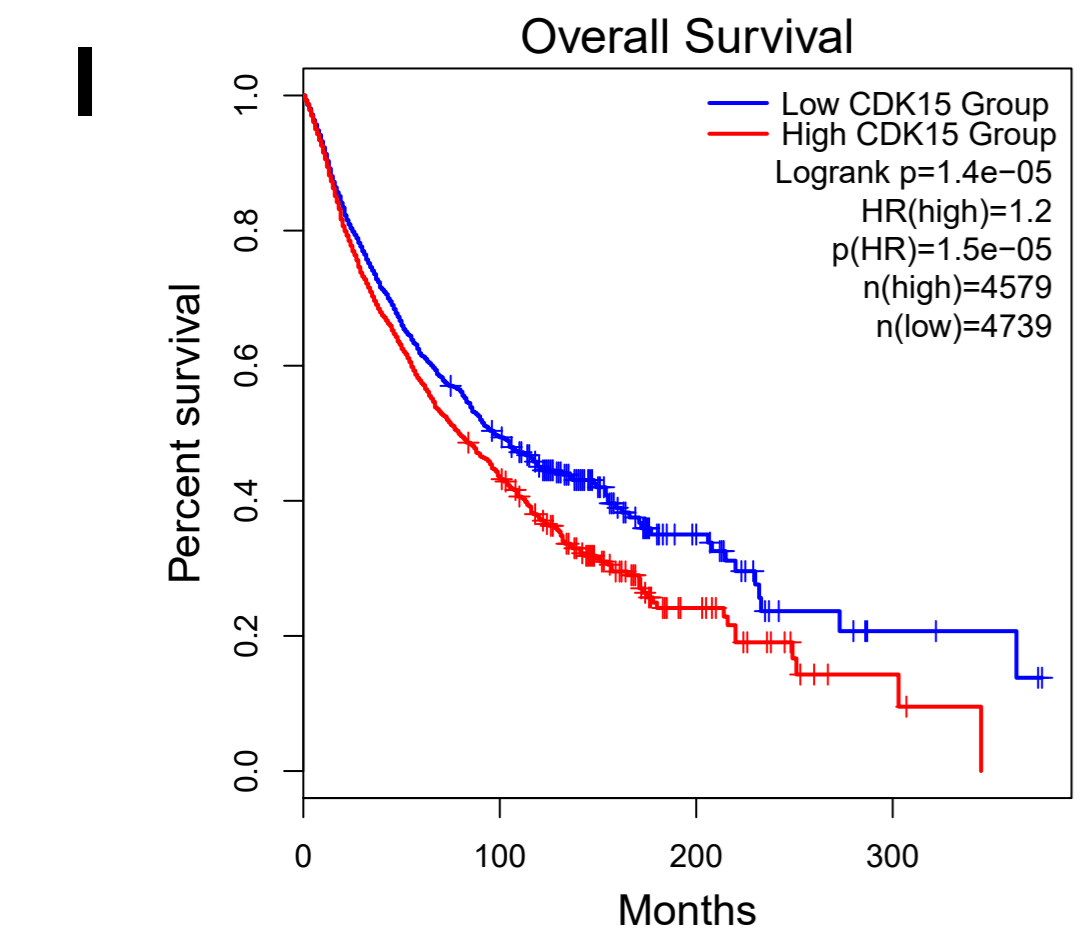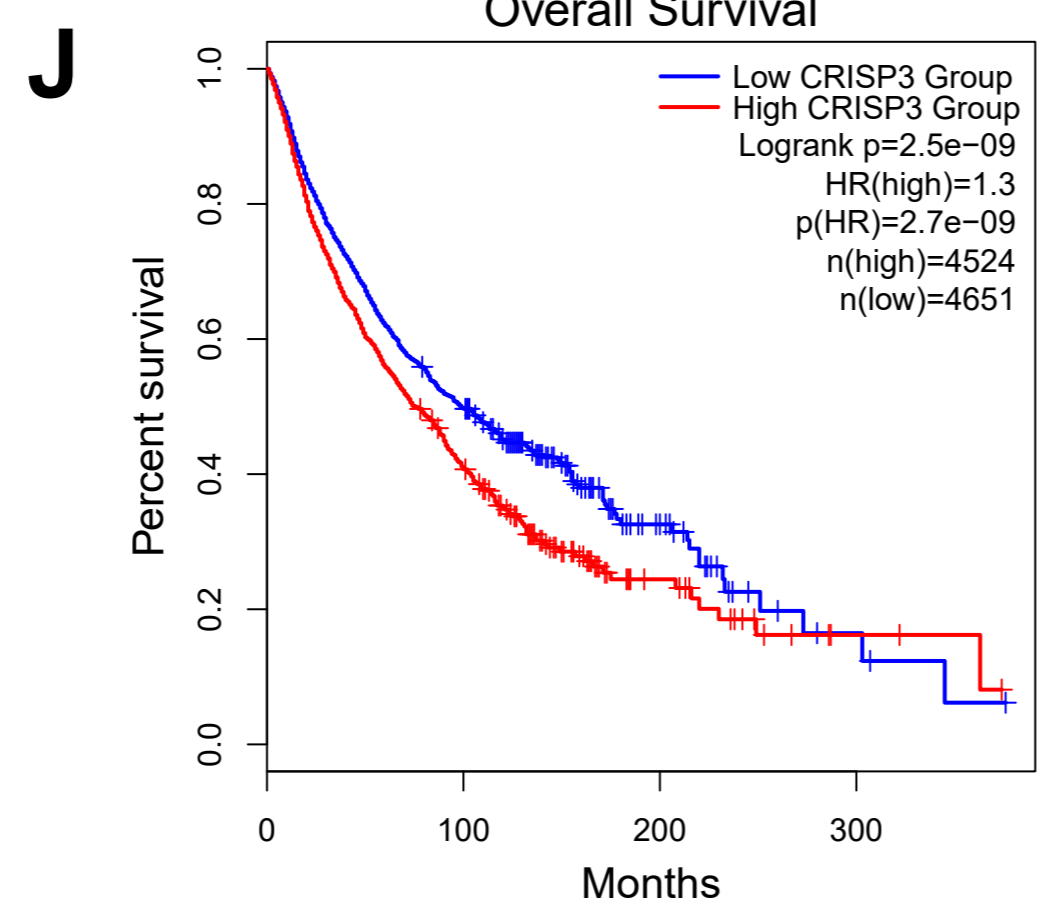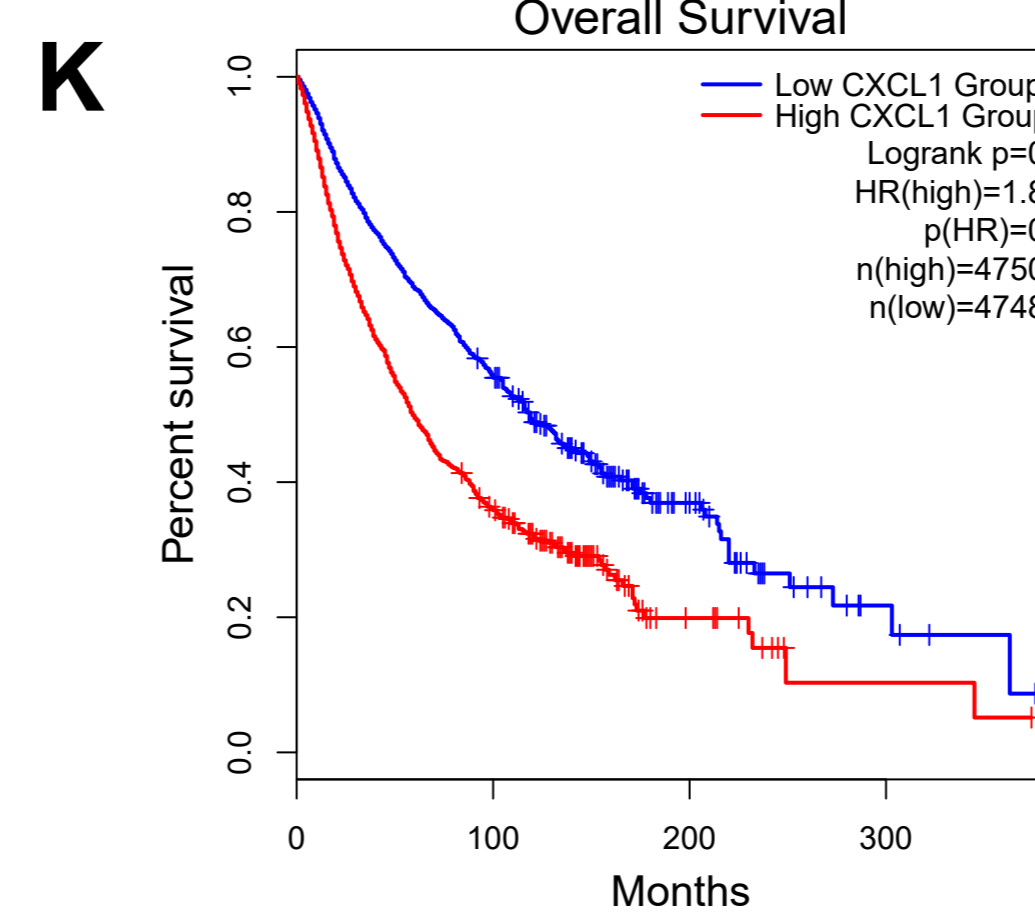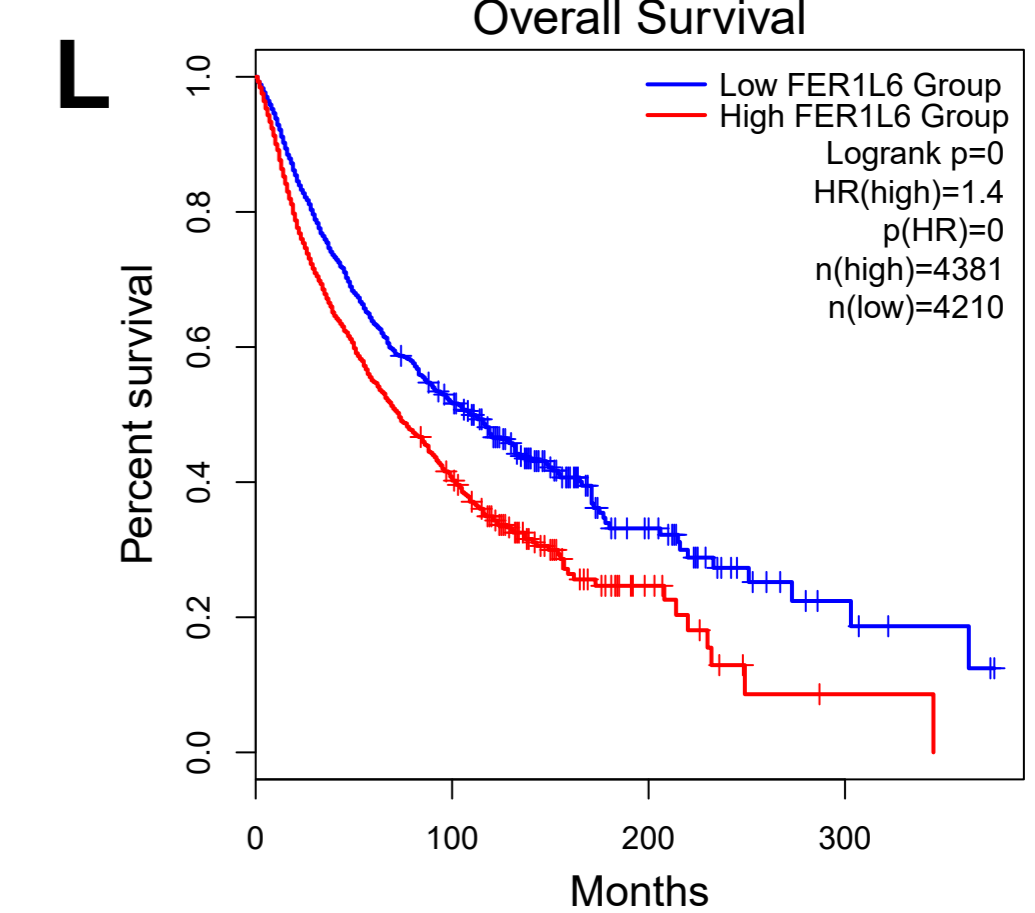

Supplement: Supplementary file 10 — Figure S9. OS of downstream genes of FOXK2. (A) The OS of BCAS3 in pan‐cancer; (B) the OS of ETV5 in pan‐cancer; (C) the OS of NKAP in pan‐cancer; (D) the OS of FBXO32 in pan‐cancer; (E) the OS of RAI2 in pan‐cancer; (F) the OS of SAMD9 in pan‐cancer; (G) the OS of TBX15 in pan‐cancer; (H) the OS of DNAH9 in pan‐cancer; (I) the OS of CDK15 in pan‐cancer; (J) the OS of CRISP3 in pan‐cancer; (K) the OS of CXCL1 in pan‐cancer; (L) the OS of FER1L6 in pan‐cancer. [file TCA-16-e15482-s010.pdf]

**A**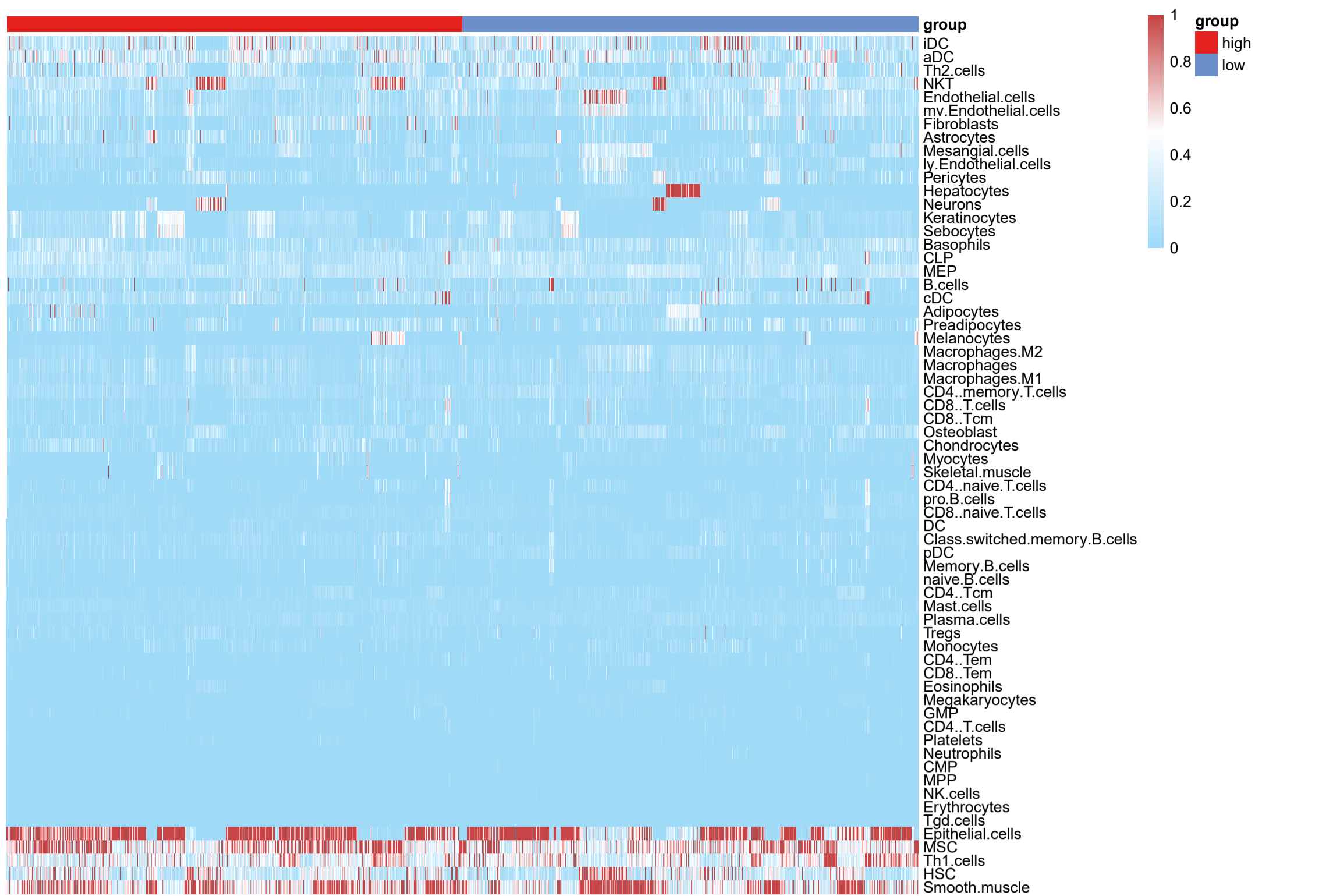**B**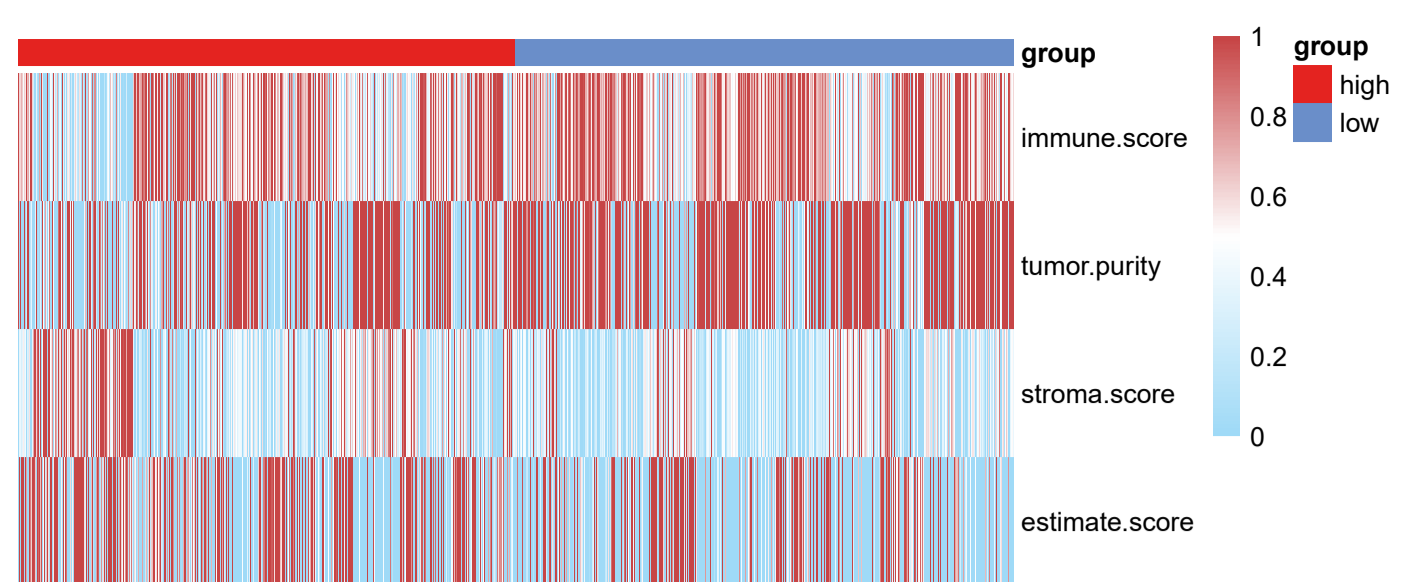**C**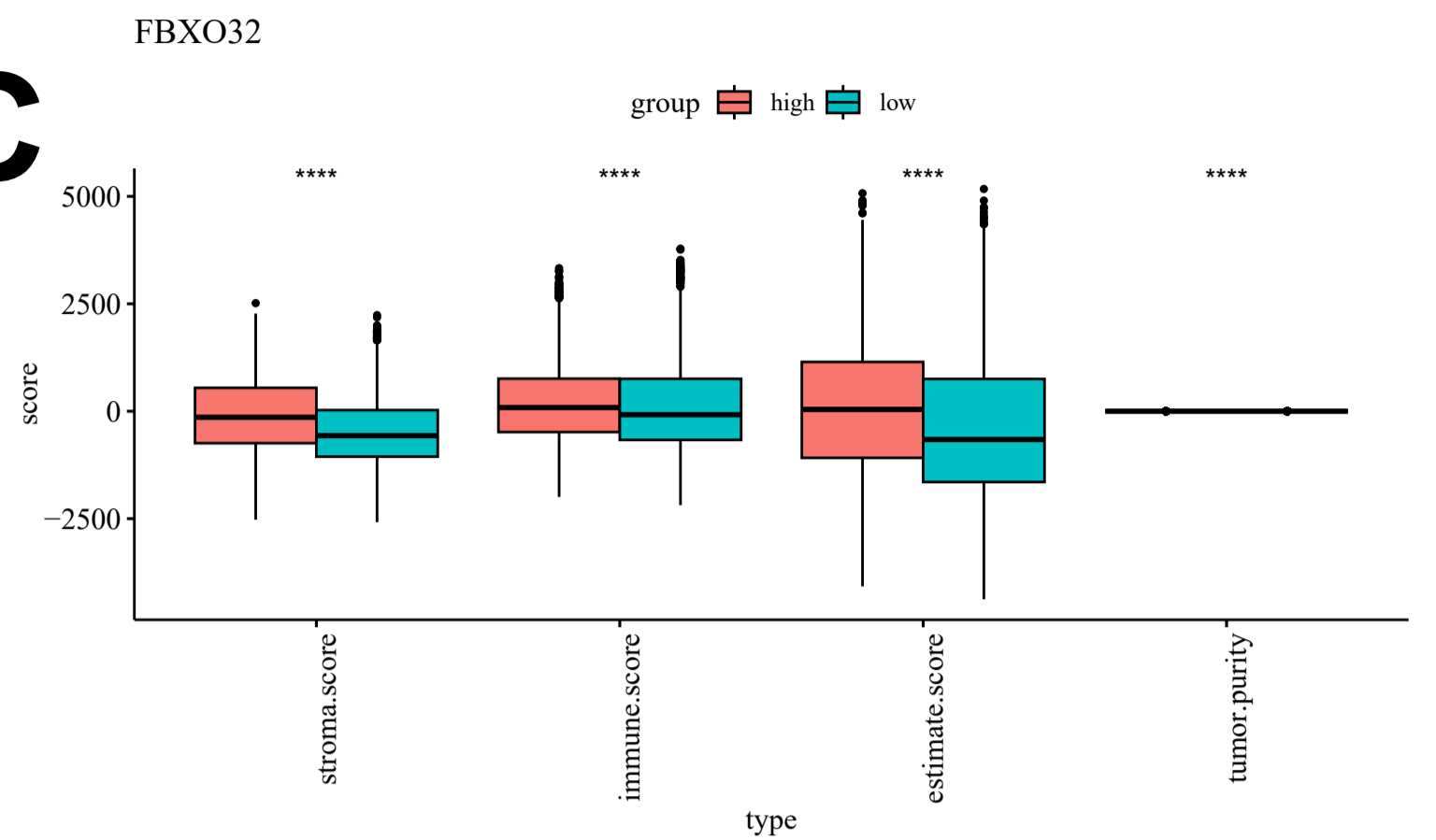**D**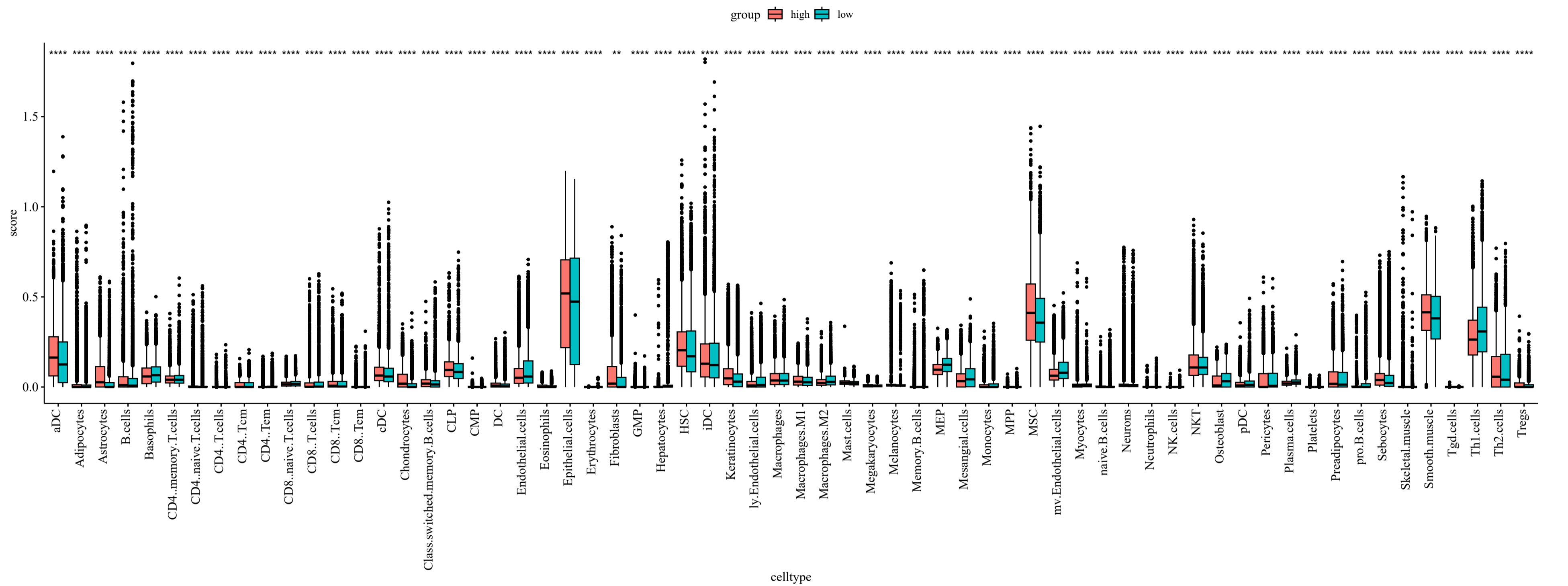**E**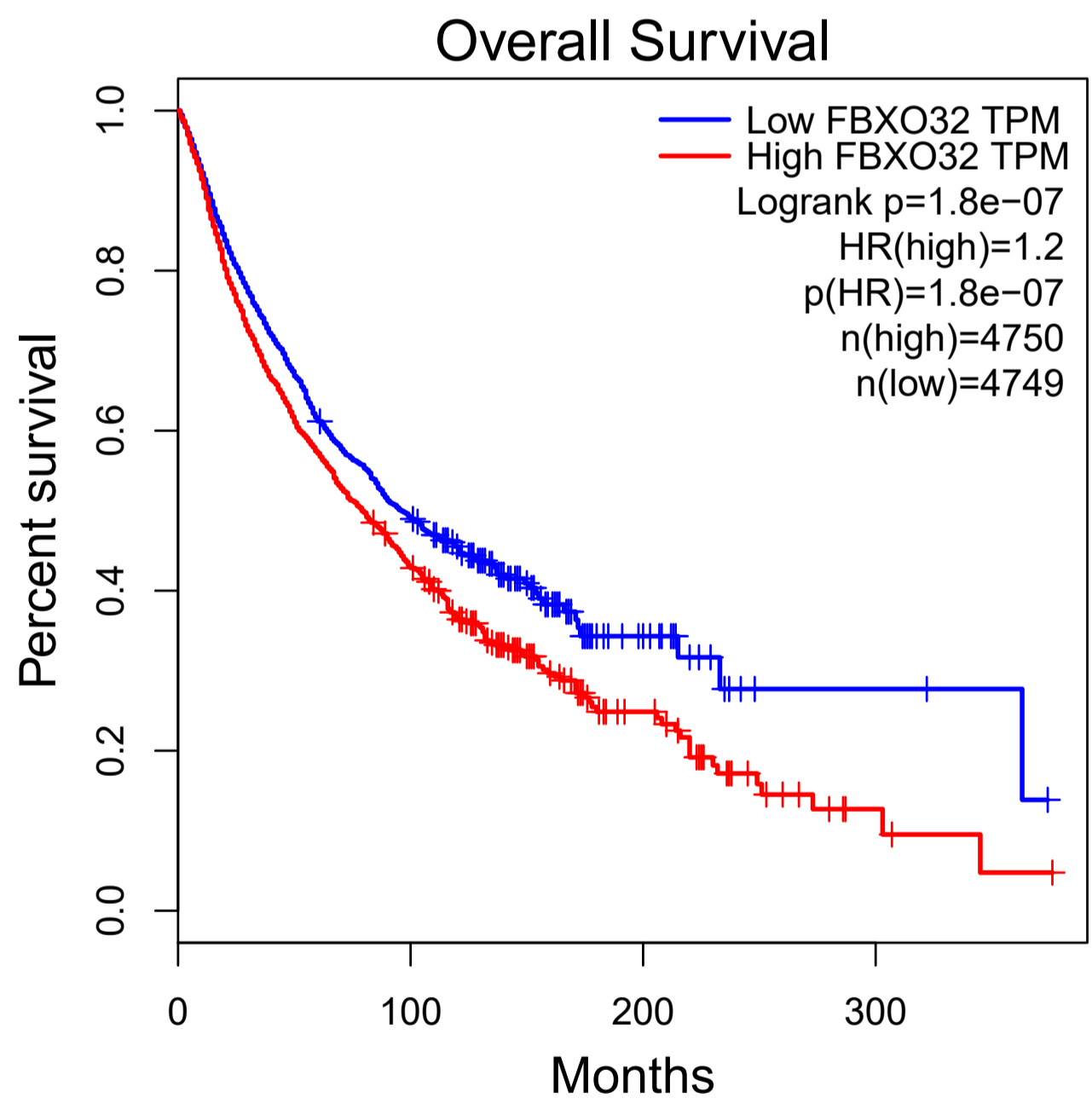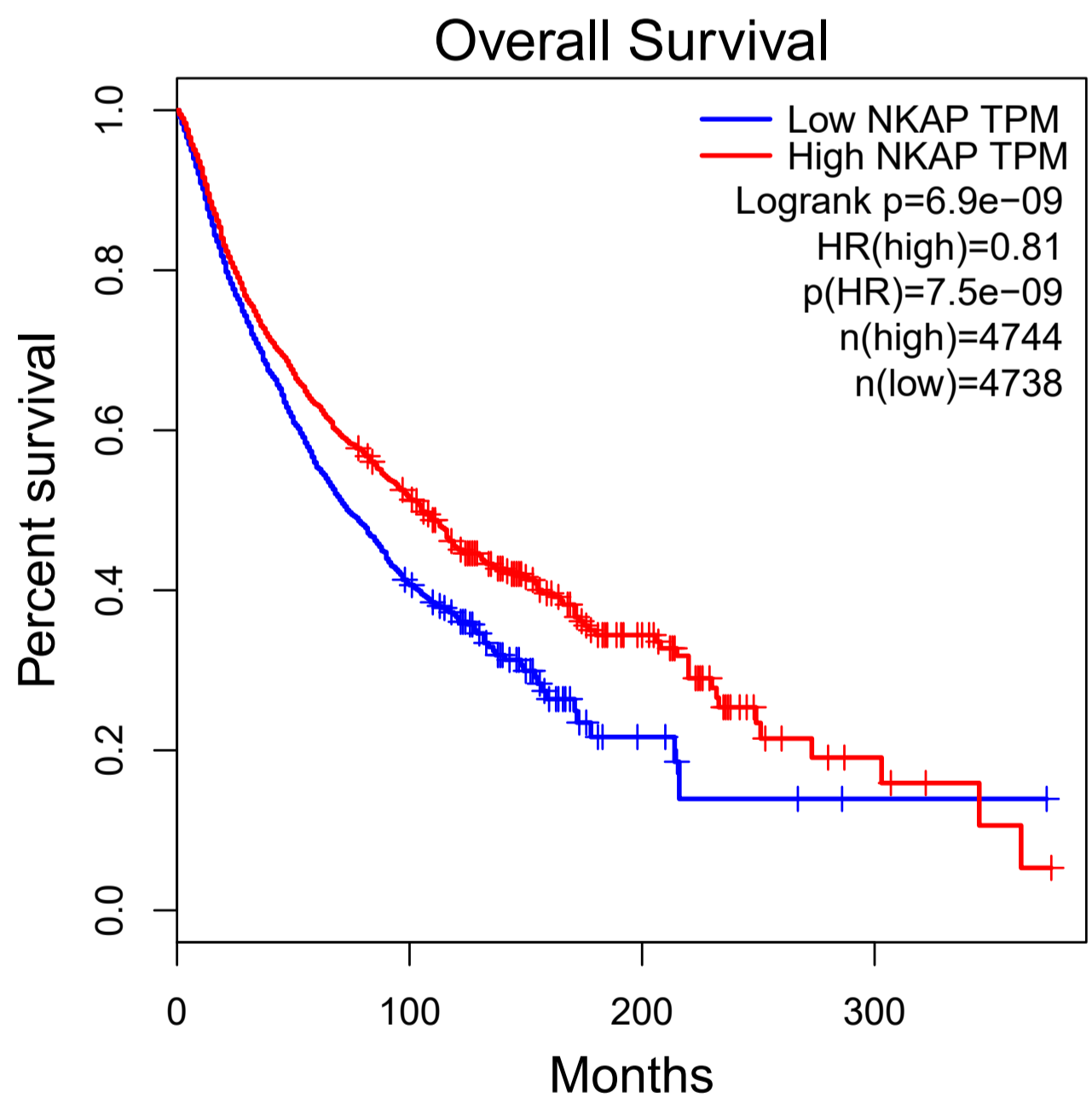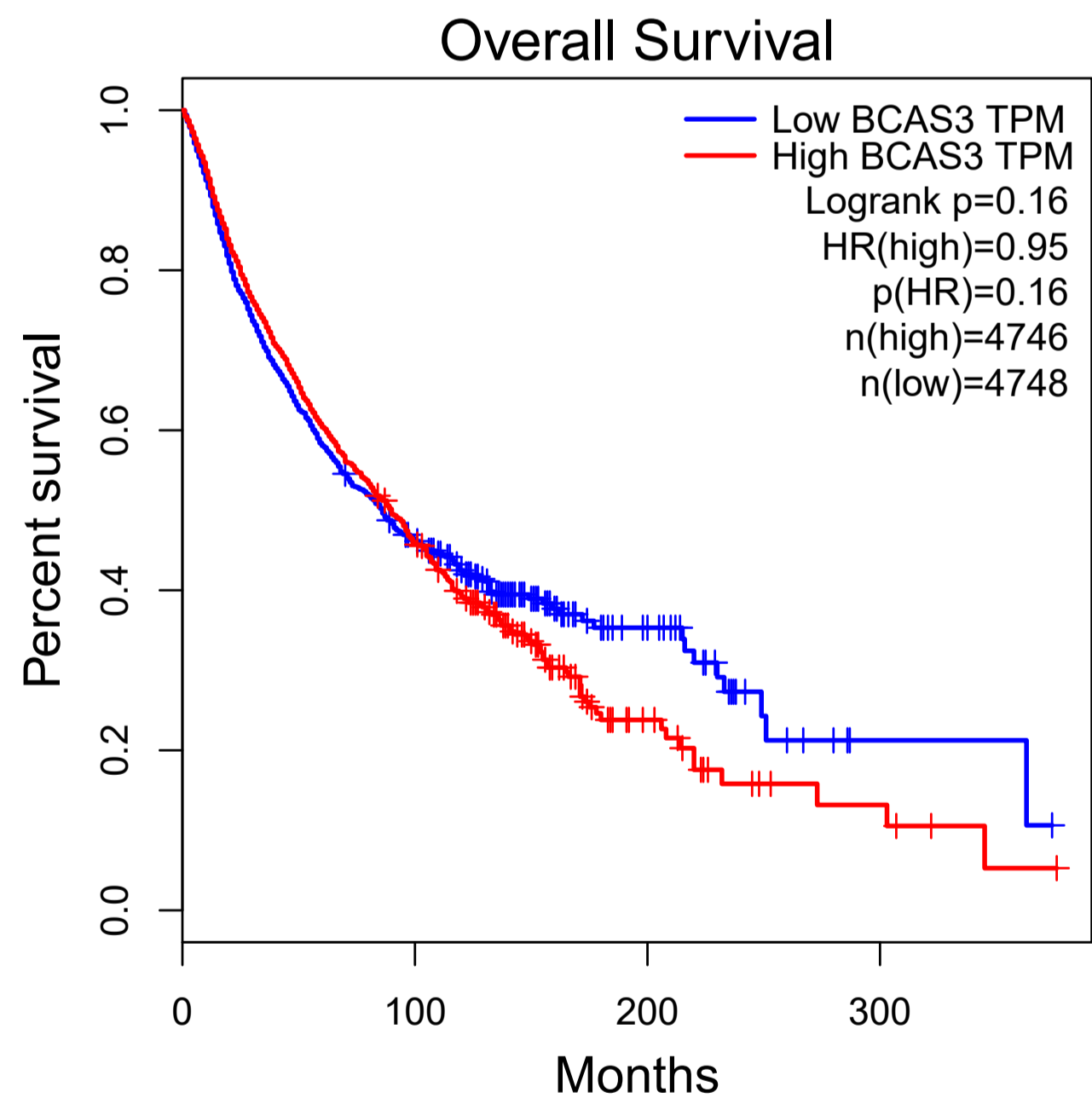**F**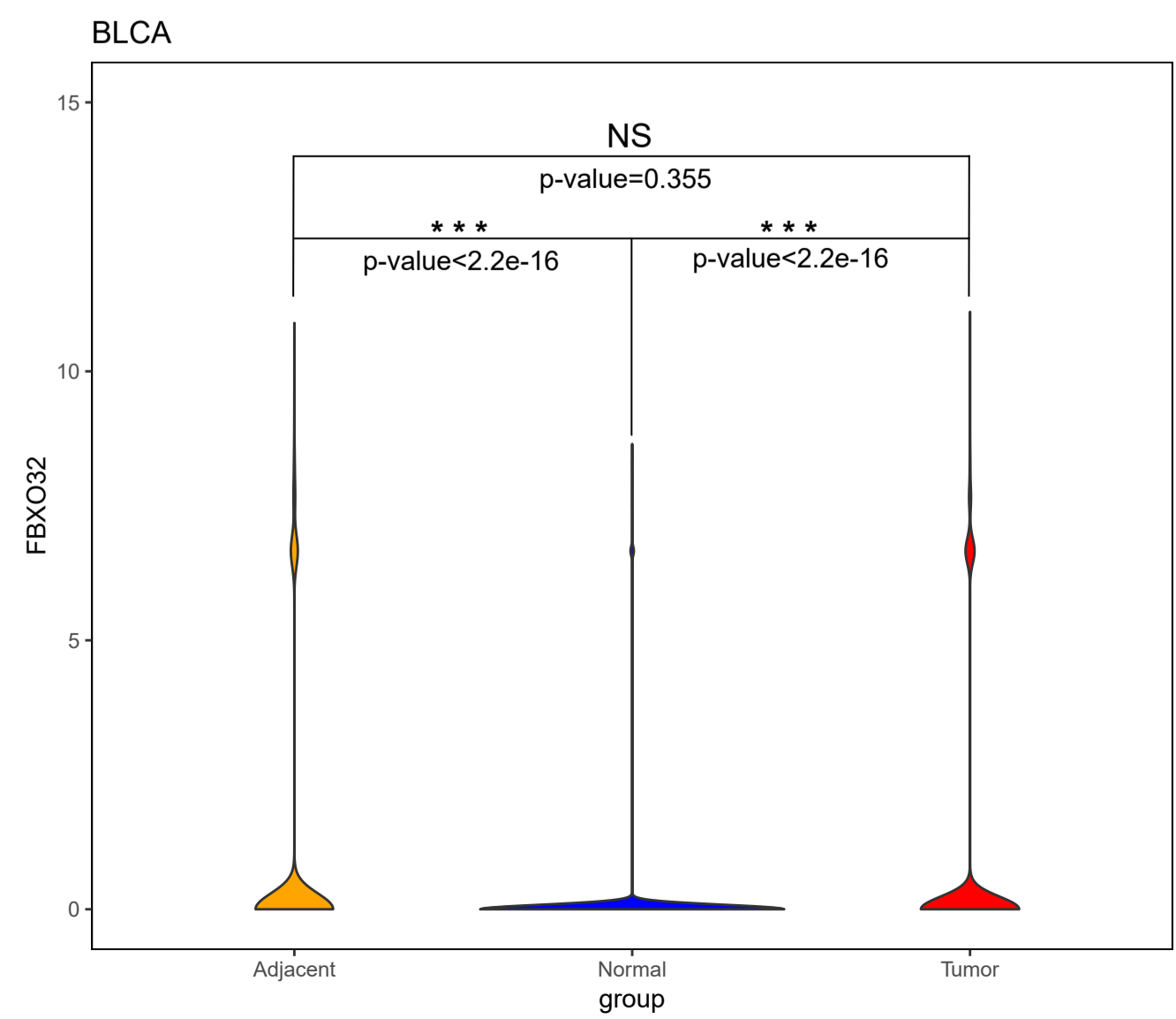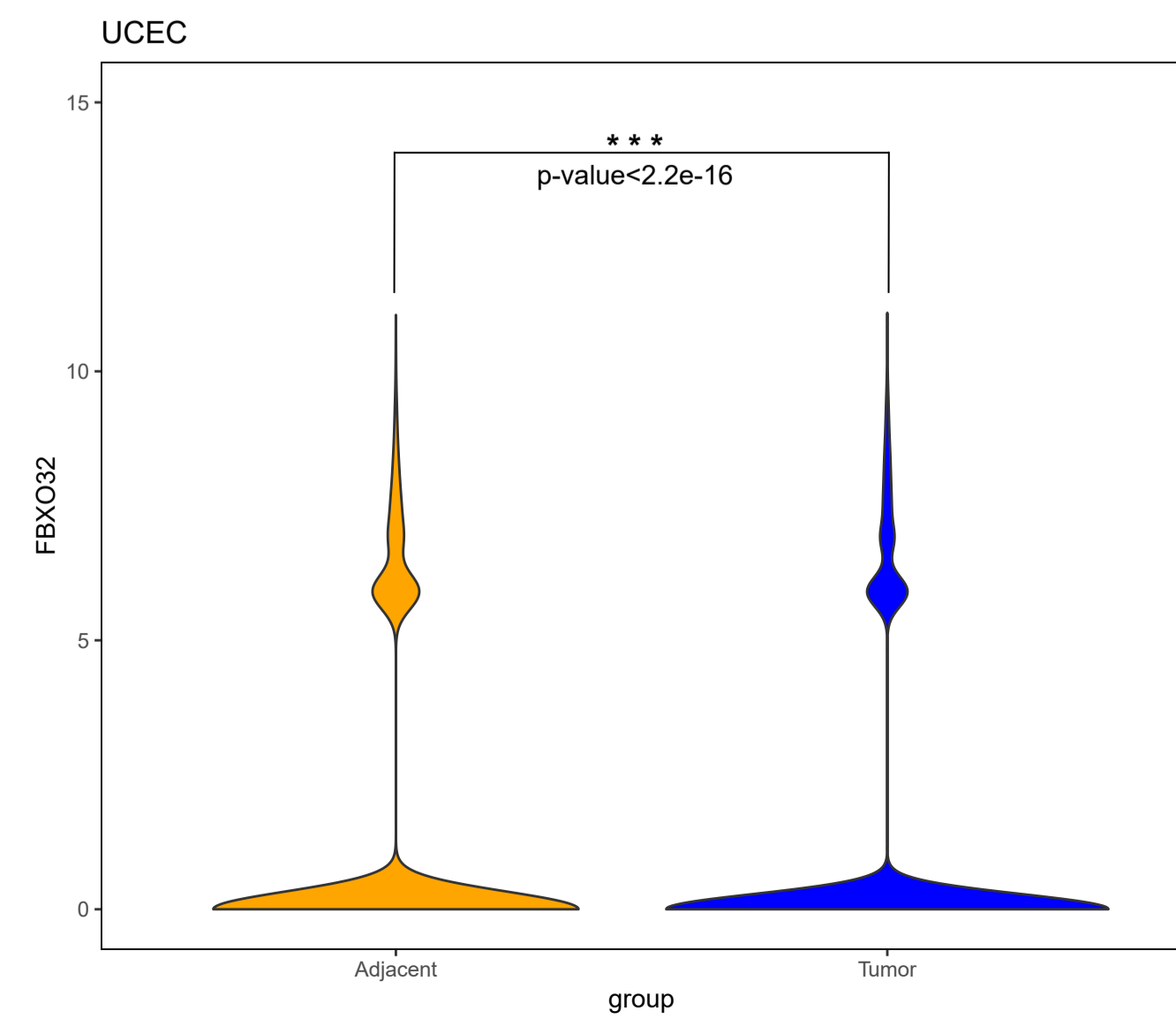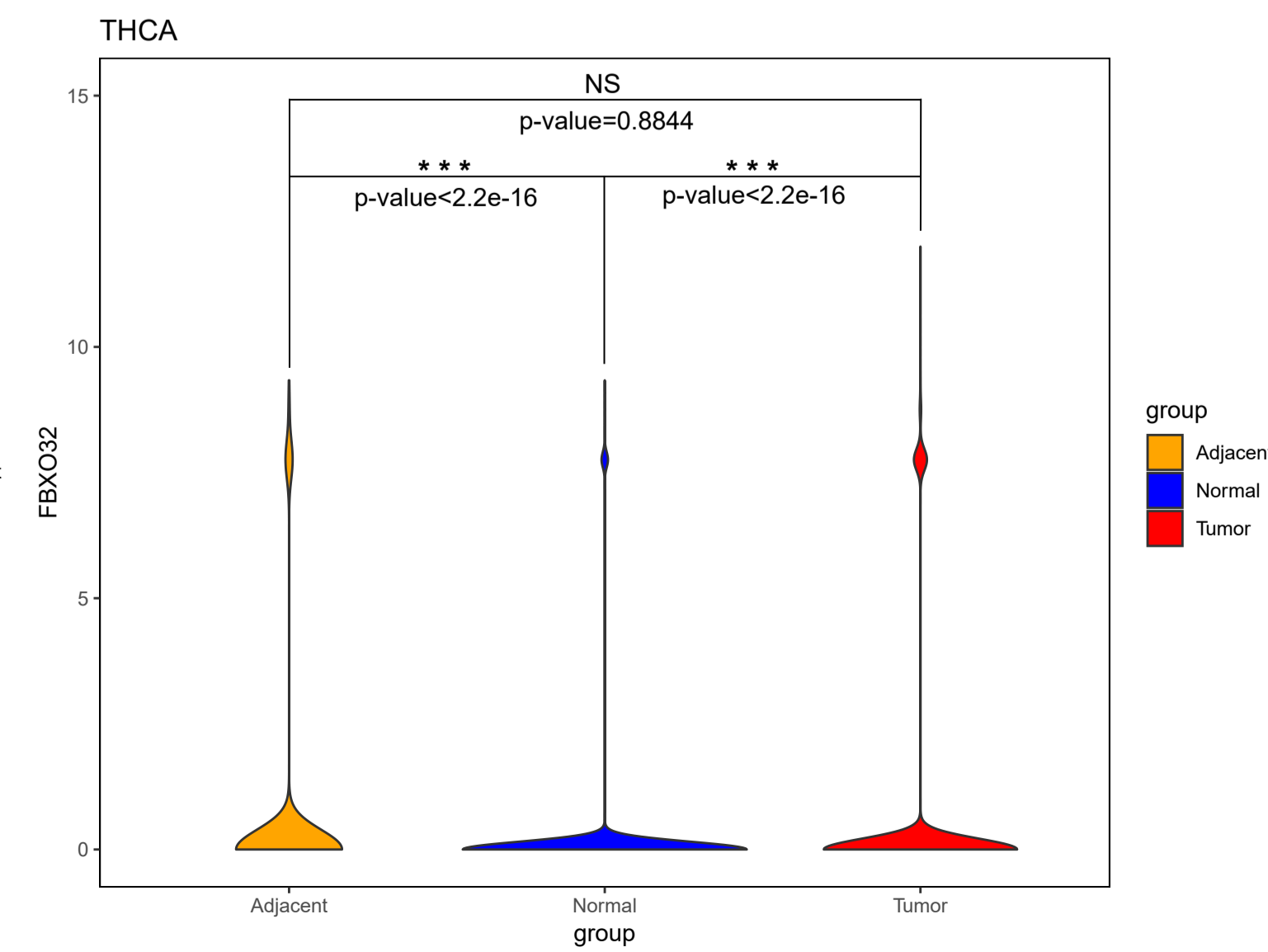

Supplement: Supplementary file 11 — Figure S10. Expression, enrichment analysis, and immune infiltration of FBXO32 in pan‐cancer. (A, D) Immune infiltration analysis of FBXO32 in pan‐cancer using xCell method; (B, C) immune infiltration analysis of FBXO32 in pan‐cancer using estimate method; (E) the OS of FBXO32, NKAP, and BCAS3 in pan‐cancer; (F) differential analysis of FBXO32 in bladder cancer, endometrial cancer, and thyroid cancer. [file TCA-16-e15482-s011.pdf]
