# Supplementary material for: The role of FOXK2–FBXO32 in breast cancer tumorigenesis: Insights into ribosome‐associated pathways
Source: Thorac Cancer. 2024 Nov 18;16(1):e15482. doi: 10.1111/1759-7714.15482 (PMC11729401; doi:10.1111/1759-7714.15482)
Supplement: Supplementary file 7 — Figure S6. Correlation between FOXK2 and immune checkpoint expression levels. Among 33 types of cancer, FOXK2 is correlated with nine immune checkpoints, with Pearson correlation coefficient on the vertical axis and − log10P. (A) VITCN1 on the horizontal axis; (B) ICOSLG; (C) CD276; (D) VEGFA; (E) ICAM1; (F) CD27; (G) IL2RA; (H) HMGB1; and (I) BTN3A1. [file TCA-16-e15482-s009.pdf]

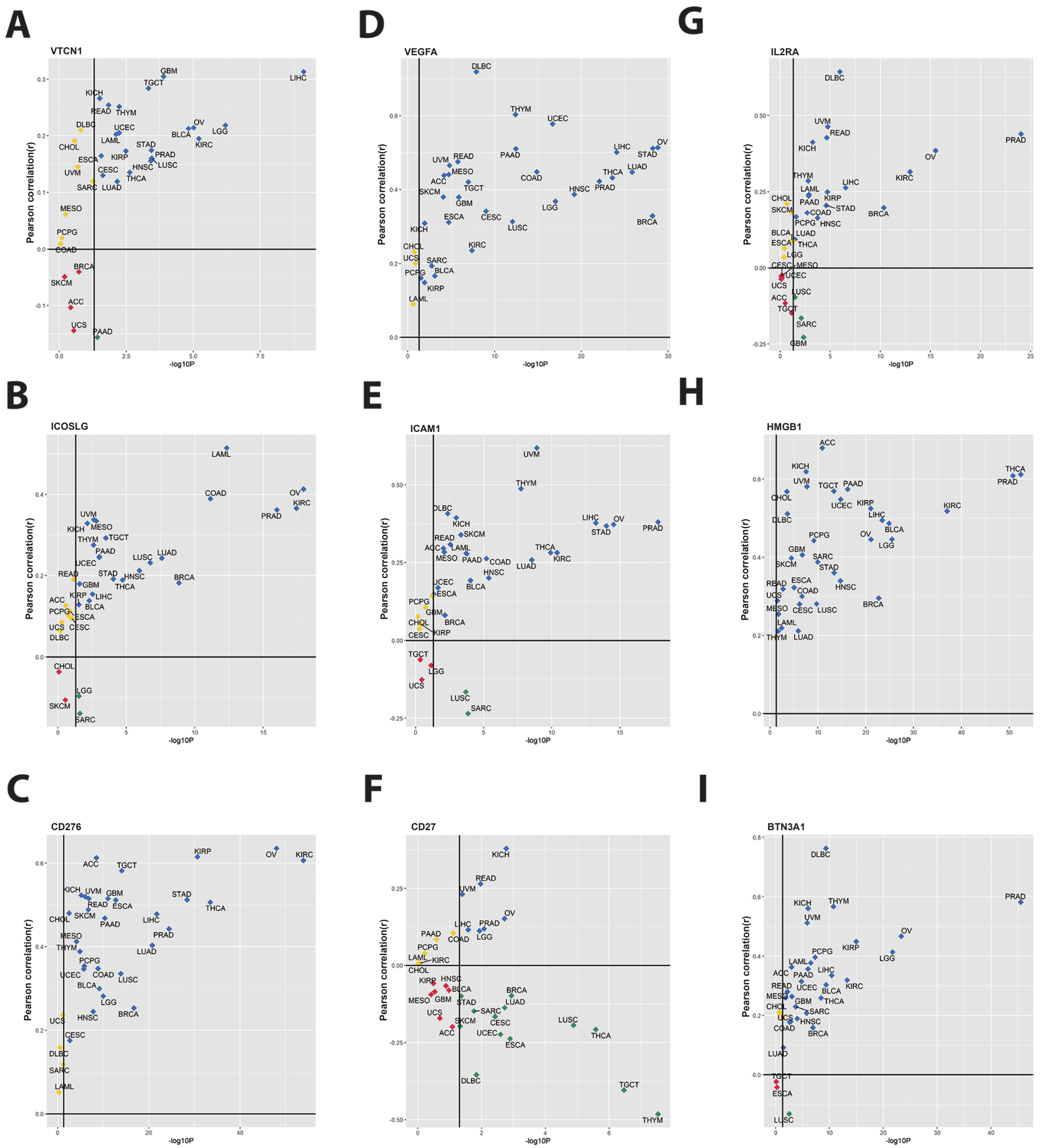

A
VTCN1
Pearson correlation(r)
-log10P
LIHC GBM TGCT KICH READ THYM DLBC UCEC CHOL LAML OV BLCA LGG KIRC STAD ESCA KIRP PRAD HNSC LUSC UVM CESC THCA SARC LUAD MESO PCPG COAD BRCA SKCM ACC UCS PAAD
B
ICOSLG
Pearson correlation(r)
-log10P
LAML COAD OV KIRC PRAD UVM KICH MESO THYM TGCT PAAD LUSC LUAD UCEC READ STAD HNSC GBM THCA BRCA ACC KIRP LIHC BLCA PCPG ESCA UCS CESC DLBC CHOL LGG SKCM SARC
C
CD276
Pearson correlation(r)
-log10P
KIRP OV KIRC ACC TGCT KICH UVM GBM STAD READ ESCA THCA CHOL SKCM PAAD LIHC PRAD MESO THYM LUAD PCPG UCEC COAD LUSC BLCA LGG UCS HNSC BRCA CESC DLBC SARC LAML
D
VEGFA
Pearson correlation(r)
-log10P
DLBC THYM UCEC PAAD LIHC STAD OV UVM READ MESO ACC TGCT SKCM GBM THCA LUAD COAD HNSC PRAD LGG ESCA CESC KICH LUSC BRCA KIRC CHOL UCS SARC PCPG BLCA KIRP LAML
E
ICAM1
Pearson correlation(r)
-log10P
UVM THYM DLBC KICH LIHC OV STAD PRAD SKCM READ ACC LAML MESO PAAD COAD THCA LUAD KIRC HNSC UCEC BLCA ESCA PCPG GBM CHOL BRCA CESC KIRP TGCT LGG UCS LUSC SARC
F
CD27
Pearson correlation(r)
-log10P
KICH READ UVM OV LIHC PRAD LGG PAAD COAD PCPG LAML KIRC CHOL KIRP HNSC BLCA MESO GBM STAD BRCA LUAD SARC UCS SKCM CESC ACC UCEC ESCA LUSC THCA DLBC TGCT THYM
G
IL2RA
Pearson correlation(r)
-log10P
DLBC UVM READ PRAD KICH OV KIRC THYM LIHC LAML KIRP CHOL PAAD STAD SKCM COAD BRCA PCPG HNSC BLCA LUAD ESCA THCA LGG CESC MESO UCEC UCS LUSC ACC TGCT SARC GBM
H
HMGB1
Pearson correlation(r)
-log10P
ACC THCA PRAD KICH UVM TGCT PAAD CHOL UCEC KIRP KIRC LIHC DLBC BLCA PCPG OV LGG GBM SARC SKCM STAD READ ESCA HNSC COAD UCS BRCA MESO CESC LUSC LAML THYM LUAD
I
BTN3A1
Pearson correlation(r)
-log10P
DLBC PRAD KICH THYM UVM OV KIRP PCPG LGG LAML LIHC ACC PAAD KIRC READ UCEC BLCA MESO GBM THCA CHOL SARC UCS HNSC COAD BRCA LUAD TGCT ESCA LUSC
