# Supplementary material for: The role of FOXK2–FBXO32 in breast cancer tumorigenesis: Insights into ribosome‐associated pathways
Source: Thorac Cancer. 2024 Nov 18;16(1):e15482. doi: 10.1111/1759-7714.15482 (PMC11729401; doi:10.1111/1759-7714.15482)
Supplement: Supplementary file 9 — Figure S8. Enrichment analysis of downstream genes of FOXK2 in K562 cell line. (A) Gene expression heatmap clustering analysis of normal and FOXK2 knockout groups in K562 cell line; (B) perform GO enrichment analysis on differentially expressed genes; (C) perform GSEA analysis on GO enrichment analysis; (D) Venn analysis showed upregulation in K562 cell lines; (E) perform KEGG enrichment analysis on differentially expressed genes; (F) perform GSEA analysis on KEGG enrichment analysis. [file TCA-16-e15482-s006.pdf]

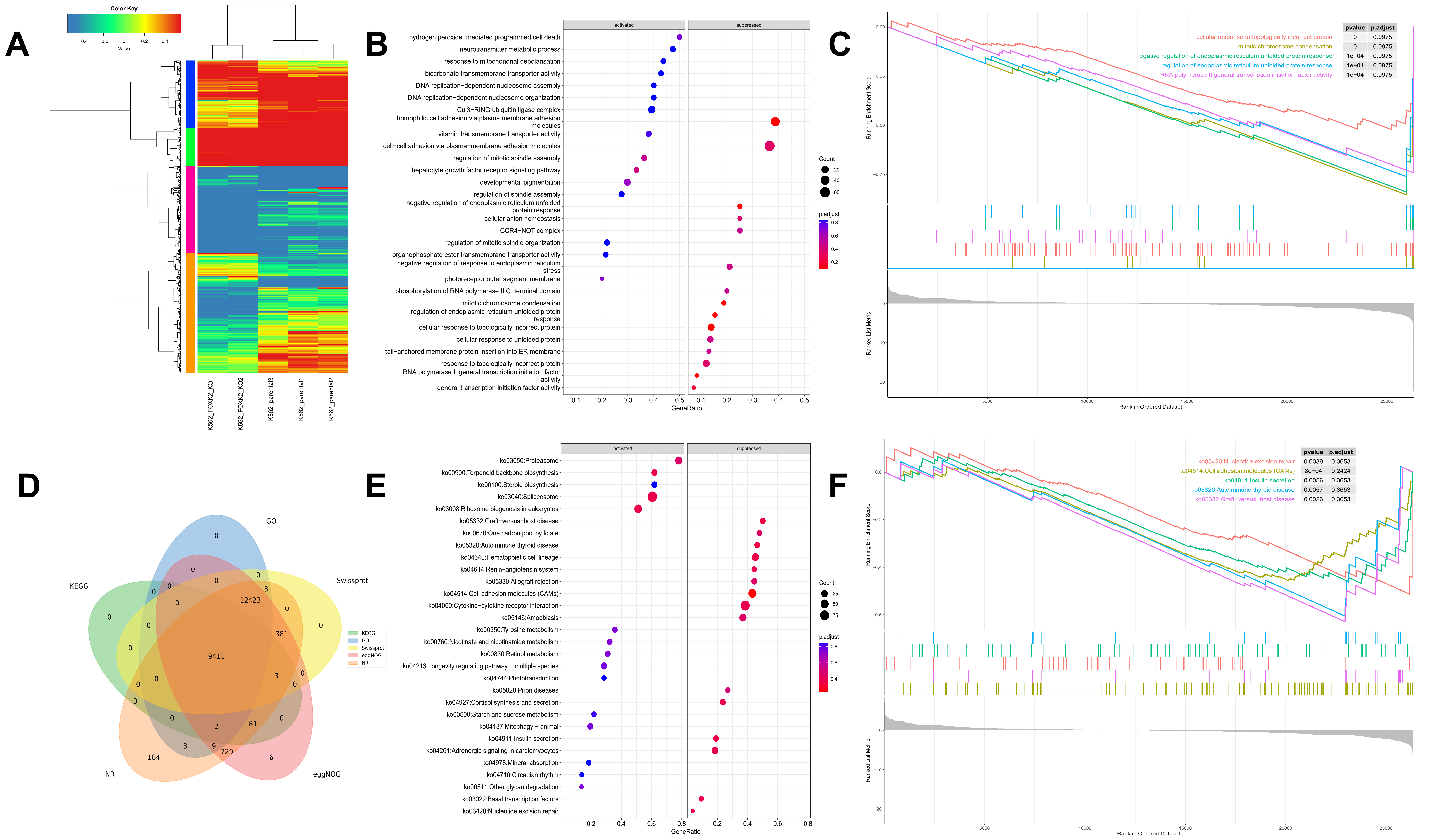

A
Color Key
-0.4 -0.2 0 0.2 0.4
Value
K562_FOXK2_KO1
K562_FOXK2_KO2
K562_parental3
K562_parental1
K562_parental2
B
activated
suppressed
hydrogen peroxide−mediated programmed cell death
neurotransmitter metabolic process
response to mitochondrial depolarisation
bicarbonate transmembrane transporter activity
DNA replication−dependent nucleosome assembly
DNA replication−dependent nucleosome organization
Cul3−RING ubiquitin ligase complex
homophilic cell adhesion via plasma membrane adhesion molecules
vitamin transmembrane transporter activity
cell−cell adhesion via plasma−membrane adhesion molecules
regulation of mitotic spindle assembly
hepatocyte growth factor receptor signaling pathway
developmental pigmentation
regulation of spindle assembly
negative regulation of endoplasmic reticulum unfolded protein response
cellular anion homeostasis
CCR4−NOT complex
regulation of mitotic spindle organization
organophosphate ester transmembrane transporter activity
negative regulation of response to endoplasmic reticulum stress
photoreceptor outer segment membrane
phosphorylation of RNA polymerase II C−terminal domain
mitotic chromosome condensation
regulation of endoplasmic reticulum unfolded protein response
cellular response to topologically incorrect protein
cellular response to unfolded protein
tail−anchored membrane protein insertion into ER membrane
response to topologically incorrect protein
RNA polymerase II general transcription initiation factor activity
general transcription initiation factor activity
GeneRatio
Count
20
40
60
p.adjust
0.8
0.6
0.4
0.2
C
Running Enrichment Score
Ranked List Metric
Rank in Ordered Dataset
pvalue p.adjust
cellular response to topologically incorrect protein 0 0.0975
mitotic chromosome condensation 0 0.0975
egative regulation of endoplasmic reticulum unfolded protein response 1e−04 0.0975
regulation of endoplasmic reticulum unfolded protein response 1e−04 0.0975
RNA polymerase II general transcription initiation factor activity 1e−04 0.0975
D
GO
KEGG
Swissprot
NR
eggNOG
12423
9411
381
184
729
81
KEGG
GO
Swissprot
eggNOG
NR
E
activated
suppressed
ko03050:Proteasome
ko00900:Terpenoid backbone biosynthesis
ko00100:Steroid biosynthesis
ko03040:Spliceosome
ko03008:Ribosome biogenesis in eukaryotes
ko05332:Graft−versus−host disease
ko00670:One carbon pool by folate
ko05320:Autoimmune thyroid disease
ko04640:Hematopoietic cell lineage
ko04614:Renin−angiotensin system
ko05330:Allograft rejection
ko04514:Cell adhesion molecules (CAMs)
ko04060:Cytokine−cytokine receptor interaction
ko05146:Amoebiasis
ko00350:Tyrosine metabolism
ko00760:Nicotinate and nicotinamide metabolism
ko00830:Retinol metabolism
ko04213:Longevity regulating pathway − multiple species
ko04744:Phototransduction
ko05020:Prion diseases
ko04927:Cortisol synthesis and secretion
ko00500:Starch and sucrose metabolism
ko04137:Mitophagy − animal
ko04911:Insulin secretion
ko04261:Adrenergic signaling in cardiomyocytes
ko04978:Mineral absorption
ko04710:Circadian rhythm
ko00511:Other glycan degradation
ko03022:Basal transcription factors
ko03420:Nucleotide excision repair
GeneRatio
Count
25
50
75
p.adjust
0.8
0.6
0.4
F
Running Enrichment Score
Ranked List Metric
Rank in Ordered Dataset
pvalue p.adjust
ko03420:Nucleotide excision repair 0.0039 0.3653
ko04514:Cell adhesion molecules (CAMs) 8e−04 0.2424
ko04911:Insulin secretion 0.0056 0.3653
ko05320:Autoimmune thyroid disease 0.0057 0.3653
ko05332:Graft−versus−host disease 0.0026 0.3653
